# Widespread Biosynthesis of Thiohistidine Dipeptides in Bacteria

**DOI:** 10.1021/jacs.6c03714

**Published:** 2026-10-01

**Authors:** Marissa L. Abbott, Jacob T. Leeman, Chase M. Kayrouz, Kendra A. Ireland, Katherine M. Davis, Mohammad R. Seyedsayamdost

**Affiliations:** † Department of Chemistry, 6740 Princeton University, Princeton, New Jersey 08544, United States; ‡ Department of Chemistry, 1371 Emory University, Atlanta, Georgia 30322, United States; § Department of Molecular Biology, Princeton University, Princeton, New Jersey 08544, United States

## Abstract

Natural products have been paramount in inspiring novel chemistry as well as providing therapeutic agents and chemical tools for biological applications. A recently emerging class of natural products, the thio/seleno-imidazoles (TSIs), contains C–S or C–Se bonds that are installed by nonheme iron sulfoxide and selenoxide synthases (NHISSs). These TSIs, which include ergothioneine, are ubiquitous, but little is known about the broader TSI chemical space or co-occurrence with other biosynthetic enzymes. Here, we use NHISSs as a bioinformatic hook to identify a widespread family of biosynthetic gene clusters that encode a combination of NHISS and ATP-grasp enzymes. This search strategy enabled the discovery of two 2-thiohistidine-containing dipeptide natural products, one of which we isolated from the original microbial host. Structural and biochemical analyses of the corresponding biosynthetic enzymes revealed a novel NHISS active site architecture and an ATP-grasp enzyme with unprecedented substrate selectivity for 2-thiohistidine. Together, these results expand the known enzymology and distribution of TSIs and establish 2-thiohistidine as a biosynthetically encoded building block, enabling future discovery and functional investigation into this new natural product family.

## Graphical abstract

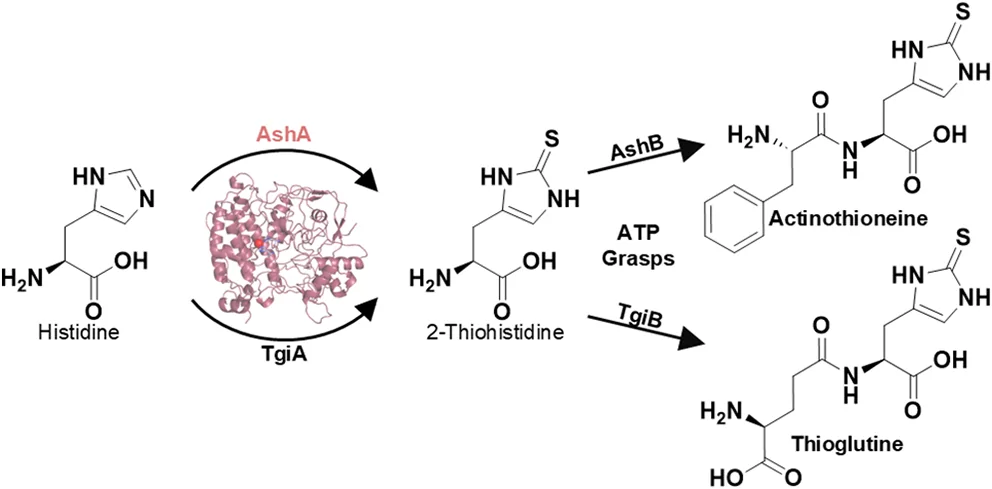

## Introduction

Natural product research has uncovered a wide array of chemical structures and biological functions and, until recently, was dominated by activity-guided discovery methods. The advent of DNA sequencing technologies and computational tools have enabled a bioinformatics-driven approach toward identifying biosynthetic gene clusters (BGCs) that encode new natural products and biosynthetic reactions.^1–3^ Thio/seleno-imidazoles (TSIs) represent one such class of natural products that have recently emerged through genome mining efforts.^4–8^ They feature 2- or 5-thio/selenohistidine moieties and the biosynthetic pathway of the most common member of this class, ergothioneine, is broadly distributed across the kingdoms of life (Figure 1).^9–14^ Ergothioneine has been associated with antioxidant and anti-inflammatory activities, and selective transporters capable of efficiently importing it have been identified in both microbial and human cells, underscoring its physiological significance.^15–18^ TSI-derived natural products, particularly those related to ergothioneine, are compelling targets for further discovery due to their biological relevance and still limited representative members.^8^

**Figure 1:**
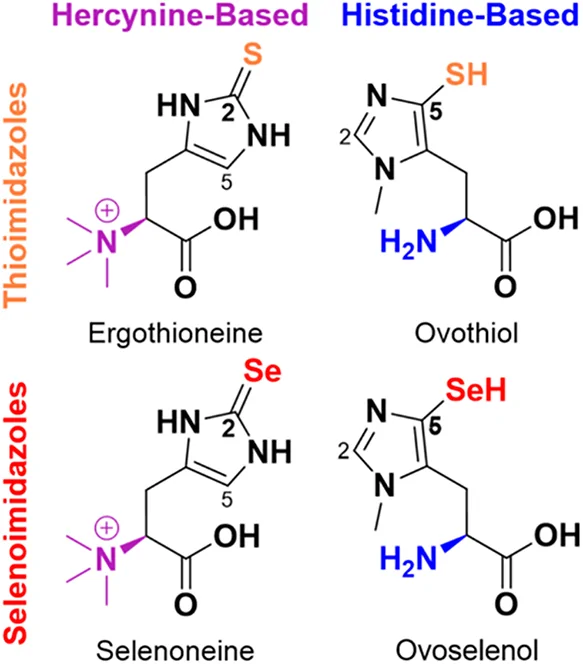
Known TSIs. Ergothioneine and selenoneine are hercynine-based with a 2-thio or 2-seleno substituent, respectively. Ovothiol and ovoselenol are histidine-based and carry a 5-thiol or 5-selenol substitution, respectively, along with side-chain *N*-methylation.

TSIs are biosynthesized by nonheme iron sulfoxide and selenoxide synthases (NHISSs), which catalyze the oxidative formation of C–S and C–Se bonds (Figures 1 and S1).^6,7,10,11^ NHISSs that produce ergothioneine have evolved independently at least four times from different thiol-utilizing precursors, highlighting the metabolic importance of this compound.^19^ In addition, we have previously demonstrated that NHISSs can synthesize two selenated TSIs, selenoneine and ovoselenol, which have significantly altered reduction potentials, making them superior antioxidants.^6–8,16,20^ Given their unique chemistry and broad distribution, NHISSs provide a powerful bioinformatic handle to uncover previously unidentified TSI-derived natural products.^8^ Apart from a recently discovered 5-thiohistidine acetylase, no other enzymes are known that modify these scaffolds outside of canonical TSI biosynthetic pathways.^21^ Moreover, natural products that contain these motifs have yet to be isolated. This knowledge gap suggests that the chemical space of TSI-derived compounds is ripe for further mining and could provide a rich source of new enzymatic reactions and natural products.

Herein, we use NHISSs as a bioinformatic hook to map protein families that are coencoded with these enzymes as part of a BGC. Among a number of modification enzymes, we were drawn to ATP-grasp enzymes that are found adjacent to NHISSs in over 100 unique instances. Bioinformatic organization of these clusters and their subsequent investigations revealed two novel and modified dipeptide natural products that incorporate 2-thiohistidine. The biosynthesis of these compounds, which we term actinothioneine and thioglutine, involves new reactions for both NHISS and ATP-grasp enzymes. An X-ray crystal structure of a previously uncharacterized NHISS involved in actinothioneine biogenesis provided insights into the structural basis of regioselectivity of thiol insertion, while reconstitution of the entire biosynthetic pathway for both natural products clarified the sequence of events involved in their production. Together, our findings suggest that TSIs are more widely incorporated into microbial natural products than previously recognized and that 2-thiohistidine may be utilized by other pathways as a biosynthetically encoded building block.

## Results and Discussion

### Bioinformatic Identification of NHISS–ATP-Grasp BGCs

We began with the hypothesis that some microbes may carry out further biosynthetic transformations on TSIs to furnish new natural products. To explore this possibility, we examined the genetic contexts of NHISSs in an unbiased fashion, given that genes involved in the same biosynthetic pathway are often clustered together on the producer’s chromosome. We first extracted 5-kb windows upstream and downstream of all NHISS genes within the NCBI database and tabulated the frequency of enzyme classes encoded within these regions. As expected, the most commonly co-occurring genes code for known TSI biosynthetic enzymes, with histidine trimethylase (*egtD*) representing by far the most common at a frequency of 69.9% (Table 1).^10^ Beyond *egtD*, we found a precipitous drop-off in frequency for the other known biosynthetic genes, including *egtC* (15.2%), *egtA* (12.1%), *senB* (8.1%), *senC* (6.3%), *egtE* (6.2%), and *ovoB* (4.4%) (Figure S1).^6,10,11^ Interestingly, only 30 gene families colocalize with NHISS genes at a frequency greater than 1%. Among these we found several classes of housekeeping genes (encoding transporters, regulators, etc.), and genes that link TSIs to interesting cellular functions (e.g., toxin/antitoxin systems or flavin storage proteins). The rather infrequent co-occurrence of biosynthetic genes suggests that the majority of TSI gene clusters function to produce one of the known TSIs.

**Table 1:** Frequency of Genes That Are Co-encoded with NHISSs^a^

CDD ID	Annotation	Frequency
egtB_TIGR03440/SenA/generic_methyltransferase	NHISS	100.0%
COG4301	EgtD	69.9%
TIGR03442	EgtC	15.2%
OpuBA/LolD/PhnC	ABC transporter ATPase	13.6%
EgtA_Cys_ligase	EgtA	12.1%
glycosyl_transferase_group_1	SenB	8.1%
PRK00943	SenC	6.3%
CsdA/pyridoxal-phosphate-dependent_transferase	EgtE	6.2%
COG2343	DUF427	5.7%
OpuBB/PBP2_AfProX_like	ABC transporter permease	5.4%
MalY	OvoB	4.4%
Lpd/PRK06370	Pyruvate dehydrogenase E3	4.0%
PhnE	ABC transporter permease	3.1%
ABC_peri_selen	Putative selenate transporter	3.1%
McrA	Endonuclease	2.9%
FabG	Dehydrogenase	2.4%
GGDEF	Diguanylate cyclase	2.0%
COG4295	Phosphatase	1.6%
PemK_toxin	Toxin/antitoxin component	1.6%
AcrR	Transcriptional regulator	1.6%
PRK03739	2-isopropylmalate synthase	1.5%
SrmB	Helicase	1.5%
PknH_C	Extracellular sensor domain	1.5%
PBP2_MalE	Periplasmic binding protein	1.4%
COG3360	Dodecin	1.3%
PlsC	Phospholipid acyltransferase	1.2%
STKc_PknB_like	Protein kinase	1.1%
HAD_PNKP-C	Phosphatase	1.0%
RacX	Amino acid racemase	0.8%
RimI	N-acetyltransferase	0.4%
LysX/AccC	ATP-grasp family	0.3%

a The conserved domain database (CDD) and bioinformatic annotations are shown.

We chose to focus on lower-frequency coencoded biosynthetic enzymes and noticed three families that could catalyze further modifications to TSIs; these code for racemases (0.8%), acyltransferases (0.4%), and ATP-grasp enzymes (0.3%, Table 1). We were especially intrigued by the presence of ATP-grasp enzymes, given that TSIs can be structurally classified as noncanonical amino acids. These enzymes play a prominent role in forming diverse peptide bonds in myriad natural product biosynthetic pathways to accommodate a wide variety of amino acid substrates.^22^ We found 100 unique instances of colocalized ATP-grasp genes, spanning at least six bacterial phyla (Figure 2). The encoded enzymes were found to be divergent, resulting in 16 distinct families grouped by sequence similarity network (SSN) analysis. Representative gene clusters for each subfamily are shown. The most abundant, subfamily 1, is encoded by *Amycolatopsis* spp. with a simple architecture consisting of an ATP-grasp, an NHISS, and a transporter. ATP-grasp subfamilies 2, 8, 11, and 13, though less abundant, share this simple arrangement, in some cases lacking the transporter. Intriguingly, several clusters encode two ATP-grasp enzymes (enzymes from subfamilies 3 and 7, 4 and 5, 5 and 12, and 5 and 9), while other clusters (containing ATP-grasp subfamilies 6, 10, 14, and 16) are adjacent to additional modification enzymes, such as a putative lyase, acyltransferase, or aminotransferase. None of these clusters have been linked to a natural product. We therefore set out to characterize their encoded functions.

**Figure 2:**
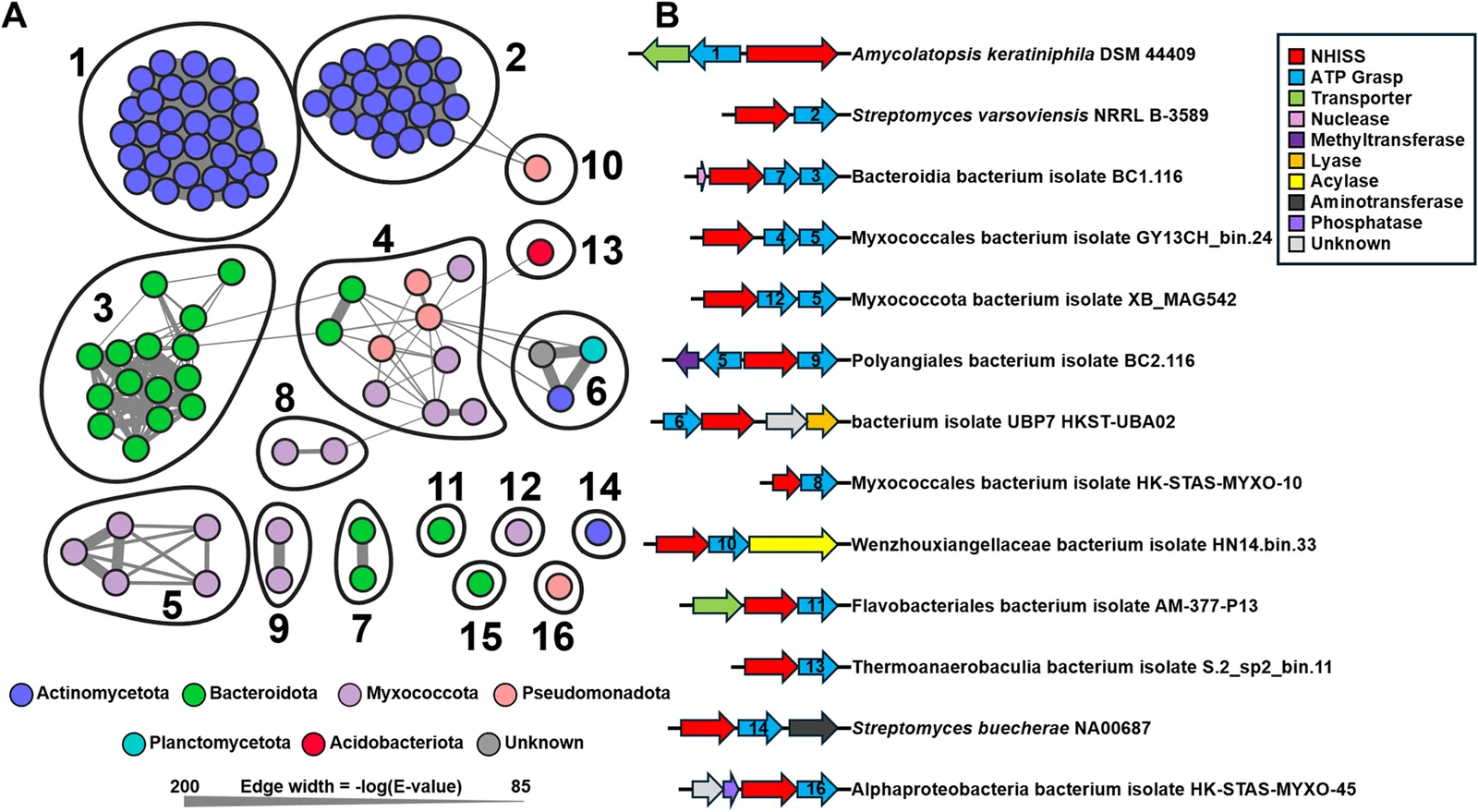
Bioinformatic detection of TSI–ATP-grasp BGCs. (A) SSN of ∼100 instances of ATP-grasp enzyme(s) that co-occur with NHISS genes, color-coded according to microbial phylum. (B) Representative clusters containing each ATP-grasp subfamily in panel (A). Note that the BGCs containing subfamilies 3–16, with the exception of 14, are from metagenome assembled genomes (MAGs).

### Actinothioneine, a Novel Thiohistidine Dipeptide

The most abundant subfamily consisted of a highly conserved three-gene cassette within *Amycolatopsis* species (subfamily 1, Figure 3A), which we have termed *ash*. The cluster encodes a dual-domain enzyme (AshA) with an *N-*terminal NHISS and a *C-*terminal methyltransferase as well as an ATP-grasp enzyme (AshB) and a transporter (AshC) from the “major facilitator superfamily” (MFS) class.^23^ The dual-domain AshA resembles OvoA, the NHISS involved in the C–S bond-forming step in ovothiol biosynthesis (Figures 1 and S1).^24,25^ The methyltransferase domain, however, is quite divergent from the rest of the class, and AshA carries a new active site motif relative to characterized NHISS enzymes. Interestingly, the methyltransferase appears in isolation in *Amycolatopsis sp*. BJA-103, which does not encode the *ash* cluster, suggesting its function is not required for biosynthesis of the target TSI (Figure S2).

**Figure 3:**
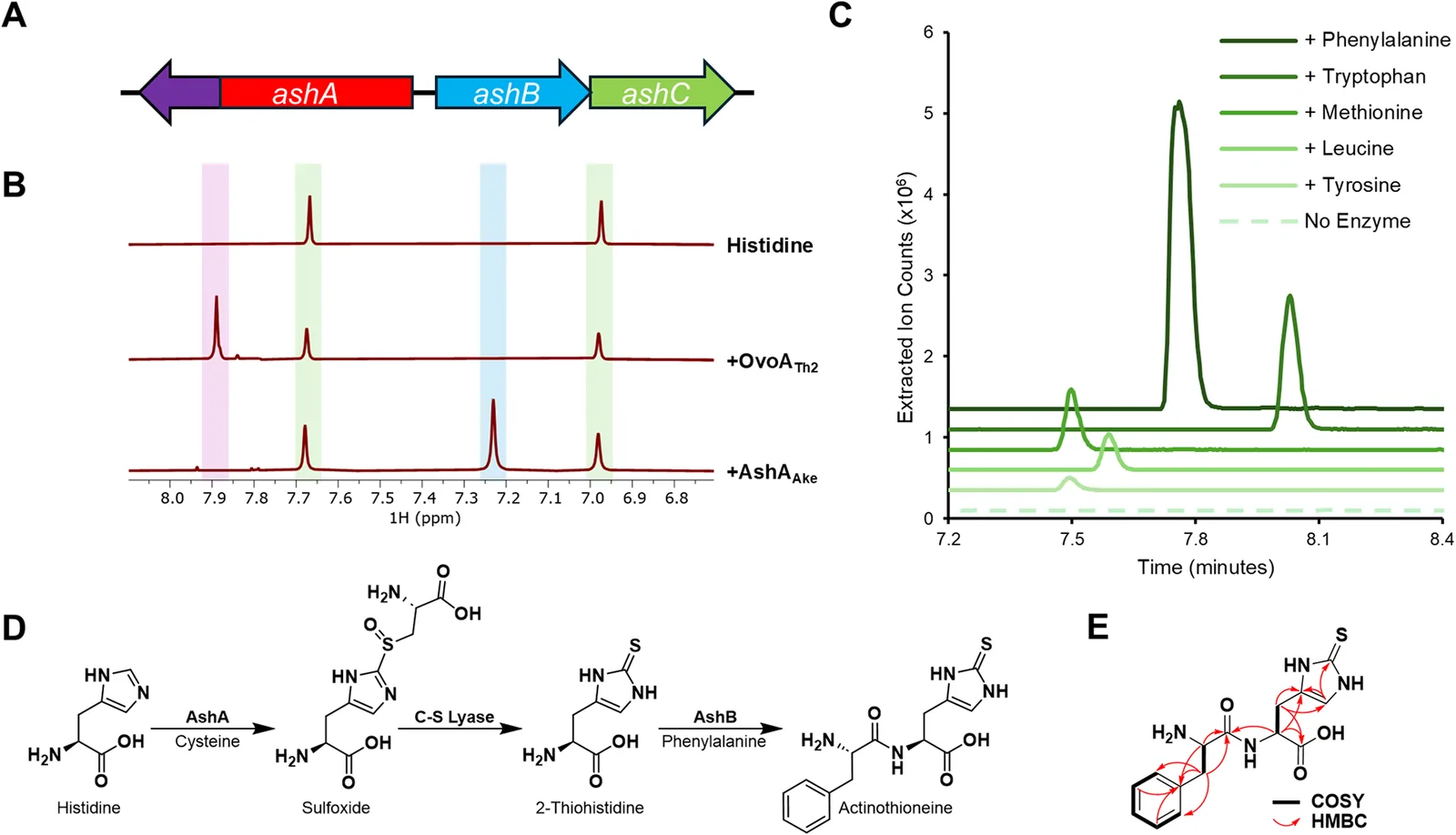
Characterization of the *ash* cluster from *Amycolatopsis keratiniphila*. (A) The *ash* gene cluster with AshA (NHISS), AshB (ATP-grasp) and AshC (transporter). (B) NMR analysis of the AshA reaction, showing linkage at the 2-carbon in the downfield ^1^H NMR region. Traces labeled Histidine, OvoA_Th2_, and AshA_Ake_ correspond to just histidine, the product from the canonical OvoA from *Hydrogenimonas thermophila* and AshA from *A. keratiniphila*, respectively. The peaks at 6.98 and 7.68 correspond to the 5- and 2-^1^H in the imidazole side chain. The peaks at 7.23 and 7.88 ppm correspond to the 5-^1^H of the ergothioneine sulfoxide precursor side chain and the 2-^1^H of the ovothiol sulfoxide precursor side chain, respectively (Figures S3 and S4). (C) Reaction of AshB with 2-thiohistidine, ATP, and all 20 canonical amino acids monitored by HPLC-MS, showing significant preference for Phe and to a lesser extent Trp. (D) Biosynthetic pathway for actinothioneine. The C–S lyase is encoded elsewhere in the genome. (E) Key NMR correlations used to solve the structure of actinothioneine.

To explore the product of this BGC, we reconstituted the pathway from *A. keratiniphila* *in vitro* with purified recombinant enzymes (Tables S1 and S2). We first focused on AshA, as NHISSs typically carry out early steps in the TSI biosynthetic pathways. Incubation of AshA with cysteine and either histidine or hercynine resulted only in the formation of histidine-linked cysteine sulfoxide, as determined by high-resolution mass spectrometry (HR-MS, [M + H]_obs_^+^ 291.0735, [M + H]_calc_^+^ 291.0758, Δppm = 7.9). To determine the regiochemistry of sulfoxide installation, the product was subjected to comparative NMR analysis with the products of OvoA and EgtB, which generate the sulfoxide precursors to 5- and 2-linked thioimidazoles, respectively (Figures 1 and S1).^10,24^ The chemical shift of the imidazole-^1^H in the sulfoxide product aligned with that of the EgtB product, indicating that the cysteine sulfoxide is installed at the 2-carbon of the imidazole ring (Figures 3B and S3,S4). This reaction would result in 2-thiohistidine, rather than 2-thiohercynine, after cleavage by a nonspecific C–S lyase, a new product for NHISS enzymes.

To investigate the function of AshB, we incubated it with 2-thiohistidine, ATP, and a mixture of all 20 canonical amino acids. The products were derivatized with monobromobimane (mBBr), a thiol- and selenol-selective reagent that adds a hydrophobic chromophore for enhanced detection and chromatography of the otherwise highly polar product.^6^ Excitingly, HR-MS analysis revealed that the ATP-grasp enzyme generates a dipeptide with 2-thiohistidine with the following preference: Phe > Trp > Met > Leu > Tyr (Figures 3C and S5). In-source fragmentation showed that 2-thiohistidine is the *C*-terminal residue (Figure S6), providing a rationale for histidine, rather than hercynine, as the substrate for AshA. Moreover, AshB did not accept 5-thiohistidine and in fact preferred unmodified histidine when given both (Figure S7). No methylation was observed, suggesting the methyltransferase domain is not active in this process. To definitively elucidate the structure of the dipeptide, we carried out the reaction of the ATP-grasp enzyme with Phe and 2-thiohistidine on a 50 mL scale, purified the peptide, and conducted thorough 1D/2D NMR analysis. The results corroborated production of the Phe–2-thiohistidine dipeptide, which we have termed actinothioneine (Figures 3D,E, S8 and Table S3), as the product of the two enzymes.

We explored cultures of *A. decaplanina*, two subspecies of *A. keratiniphila*, and *Streptomyces albus* transformed with the *ash* cluster, all grown in several media, but we were unable to detect actinothioneine, perhaps because the *ash* cluster is sparingly expressed under these growth conditions (Tables S2 and S4). We considered that actinothioneine may be further altered but did not observe any products with plausible modifications. Meanwhile, overexpression of *ashAB* in *E. coli* resulted in formation of actinothioneine, demonstrating the products of these enzymes *in vivo* and a preference of AshB for Phe only (Table S5 and Figure S9). This finding, moreover, suggests that the dipeptide is the product of the *ash* cluster and that a C–S lyase is borrowed from elsewhere in the cell to complete synthesis of this novel natural product (Figure 3D).

### X-ray Crystal Structure of AshA

Although AshA is phylogenetically closest to OvoA, it exhibits EgtB-like regioselectivity for sulfoxide insertion. We thus sought to define the structural basis of AshA’s unexpected reactivity by solving its X-ray crystal structure in complex with histidine, which we refined at 1.11 Å resolution (Protein Data Bank (PDB) accession code 9PHY, Figure 4, Table S6, and Figure S10). AshA’s overall architecture exhibits the canonical NHISS fold previously described by our group and others, consisting of a DinB-like *N*-terminal four-helix bundle and a formylglycine-generating enzyme (FGE)-like, loop-rich *C*-terminal domain (Figure 4A).^7,19,25–28^ The NHISS active site resides at the interface of these two domains, where a three-His facial triad coordinates the mononuclear iron cofactor. In addition to the NHISS domain, the *ashA* gene encodes a *C*-terminal methyltransferase domain. By our analysis, this domain appears to be a vestigial remnant from the NHISS family’s evolutionary transition from OvoA to AshA, as we were unable to determine its catalytic function. Notably, no electron density was observed for this domain, and it could not be accommodated within the crystal lattice, suggesting that a truncated version of the enzyme was crystallized.

**Figure 4:**
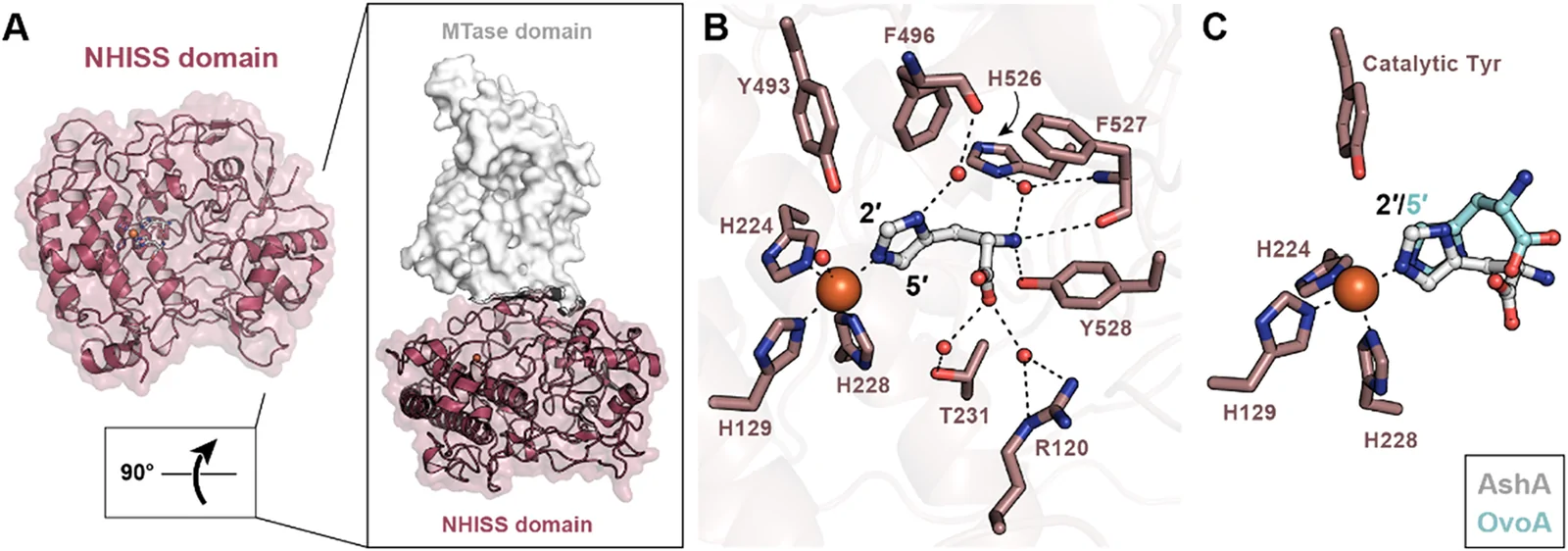
X-ray crystal structure of AshA. (A) Overall architecture of AshA. Although the electron density for this region is unresolved, AshA contains a vestigial, OvoA-like *C*-terminal methyltransferase domain. (B) Hydrogen-bonding interactions involved in histidine binding by AshA. (C) Comparison of the histidine orientation in the active sites of AshA (white) and OvoA (cyan). Rotation of the ligand backbone accounts for the observed regioselectivity differences, with bond formation occurring at the carbon positioned toward the catalytic Tyr residue.

Inspection of the active site in our crystallographic model reveals the structural basis for AshA’s unique 2-regioselectivity with histidine (Figure 4B). In contrast to the NHISS enzymes OvoA and OvsA, which catalyze C–S/Se bond formation at the 5-carbon of histidine, the active site of AshA reorients histidine such that the imidazole 2-carbon is positioned toward the catalytic Tyr residue (Y493, Figure 4C). This tyrosine has been shown by the Liu and Seebeck laboratories to be essential for sulfoxide synthase activity in NHISS enzymes, and structural analyses from our group have supported the idea that substrate positioning relative to this residue largely determines bond-forming regioselectivity in NHISSs.^7,26,29–32^ The substrate orientation in AshA more closely resembles that of hercynine binding in the NHISS enzymes EgtB and SenA, in which the ligand backbone is rotated downward. This binding mode is stabilized by a hydrogen bonding network (Figure 4B). Specifically, direct hydrogen bonds are formed between the substrate’s amino group and the hydroxyl of Y528 as well as the backbone carbonyl of F527. In addition, water-mediated hydrogen bonds are observed between: (i) the histidine imidazole π-nitrogen and backbone carbonyl of F496, (ii) the histidine carboxylate and side chains of R120 and T231, and (iii) the histidine amino group and H526/F527.

Visual assessment of these contacts suggests that the presence of carboxylate-binding residues R120 and T231 may influence biosynthetic activity at the His 2-carbon by helping orient the ligand backbone for the observed regioselectivity (Figure 4B). However, comparison of the active sites with NHISS members that act at the imidazole 5-carbon (OvoA and OvsA) indicates that this relationship is not absolute. OvoA retains these residues despite its native reactivity with the 5-carbon of histidine, although it has been shown to catalyze non-native 2-carbon sulfoxidation with methylated substrates.^33^ In contrast, OvsA, which inserts the C–Se bond exclusively at the 5-carbon, lacks these residues.^7^ Taken together, these comparisons suggest that while these carboxylate-binding contacts may be necessary to enable the reaction at the 2-carbon, they are not sufficient on their own, and additional structural determinants must contribute to the observed regioselectivity for each NHISS subfamily.

### Thioglutine, a Thiohistidine Natural Product Isolated from a Streptomycete

Motivated by the discovery of actinothioneine from the *ash* cluster as part of subfamily 1, we investigated additional biosynthetic clusters in the network, next focusing on subfamily 2 (Figure 2). This cluster, termed *tgi* for thioglutine, consists of genes coding for an NHISS (TgiA) and an ATP-grasp enzyme (TgiB) and occurs in 15 *Streptomyces* species (Figure 5A). TgiA from *Streptomyces varsoviensis* shows ∼30% sequence similarity to AshA, suggesting it too generates a 2-carbon-linked Cys-His sulfoxide, whereas TgiB is a previously uncharacterized protein from the RimK Pfam. It shares domain architecture with canonical polyglutamylases, such as those that modify ribosomal protein S6.^22,34^ However, low sequence similarity and gene context suggest a distinct substrate preference. Reaction of *S. varsoviensis* TgiA with His and Cys resulted in the formation of the anticipated 2-thiohistidine sulfoxide, as confirmed by ^1^H NMR spectral analysis of aromatic imidazole protons (Figure S3). Moreover, subsequent treatment of the product with a C–S lyase (CsdA) resulted in formation of 2-thiohistidine, as visualized by mBBr derivatization and HR-MS analysis ([M + H]_obs_^+^ 378.1231, [M + H]_calc_^+^ 378.1231, Δppm <0.1, Table S7). Next, reaction of TgiB from the same strain with 2-thiohistidine and all canonical amino acids in competition showed strong preference for Glu and resulted in the formation of Glu–2-thiohistidine dipeptide, a product that we have termed thioglutine (Figure 5B). The structure of this product was confirmed by HR-MS ([M + H]_obs_^+^ 507.1656, [M + H]_calc_^+^ 507.1656, Δppm <0.1) and subsequently by NMR (see below).

**Figure 5:**
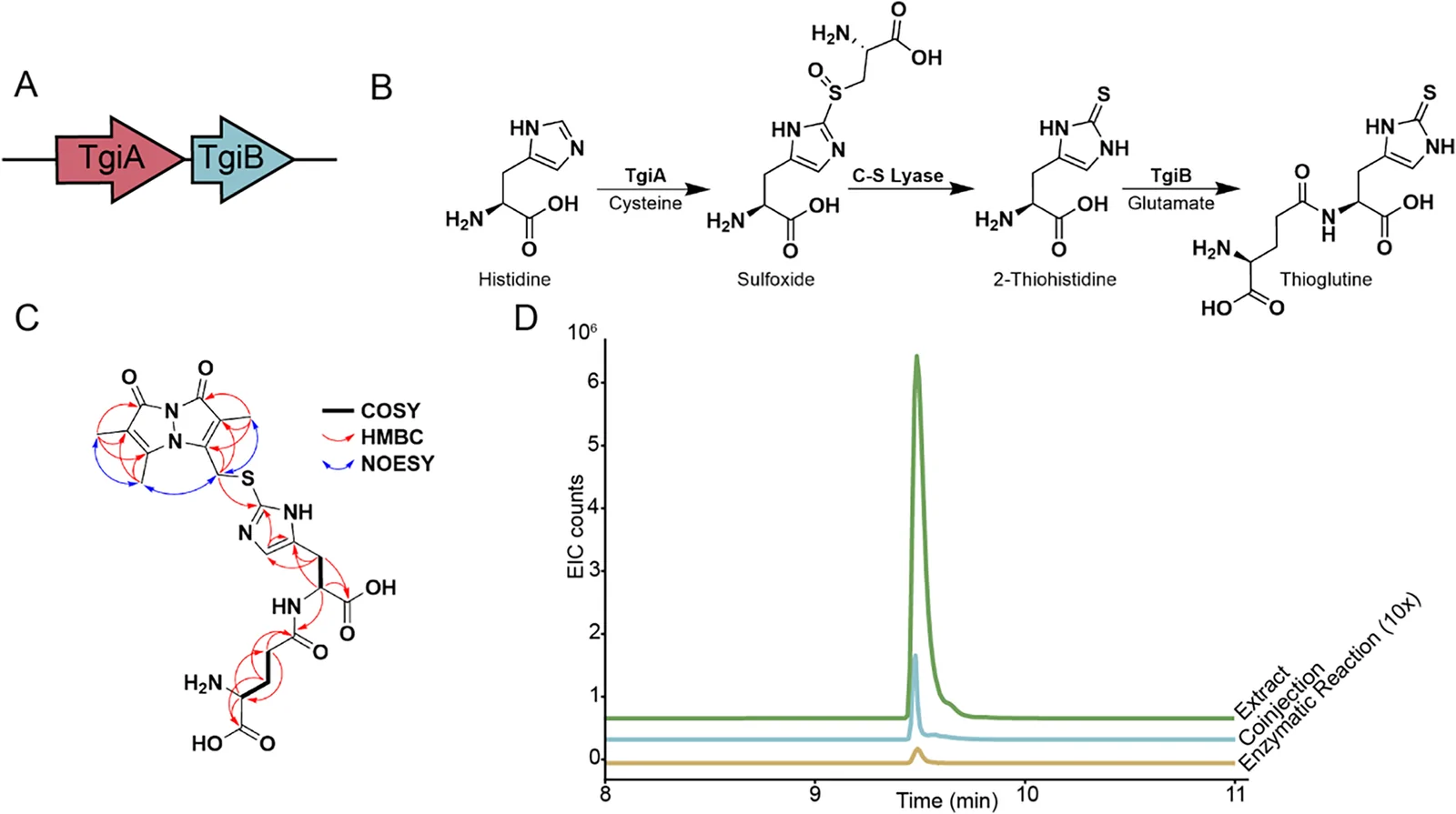
BGC, biosynthetic pathway, and structural elucidation of thioglutine. (A) The *tgi* BGC from *S. varsoviensis* encoding TgiA (NHISS) and TgiB (ATP-grasp) (B) Proposed biosynthetic pathway of thioglutine, showing the order of reactions catalyzed by enzymes encoded in the *tgi* cluster. Note that a C–S lyase is encoded elsewhere in the genome. (C) 1D/2D NMR correlations used for structural elucidation of mBBr-derived thioglutine. Note NMR correlations that indicate a γ-linked Glu residue. (D) HPLC–MS extracted ion chromatograms of authentic thioglutine from *S. varsoviensis* (“Extract”), thioglutine obtained from *in vitro* reactions with TgiAB and C–S lyase (“Enzymatic Reaction (10x)”) and coinjection of the two; the latter shows a single coeluting peak.

Having identified the *in vitro* product, we sought to identify the authentic natural product encoded by the *tgi* cluster. *S. varsoviensis* cultures were grown in ISP2 medium, harvested after 3 days, and derivatized with mBBr. HR-MS analysis of derivatized extracts revealed a new peak at *m*/*z* 507.1656, consistent with an mBBr-tagged Glu–2-thiohistidine dipeptide. To definitively assign its structure, we isolated thioglutine from an 11 L *S. varsoviensis* production culture, derivatized it with mBBr, and purified it to homogeneity. The mBBr-derivatized form of thioglutine was then characterized with 1D/2D NMR spectroscopy (Figure 5C, Table S8, Figure S11), which provided numerous long-range correlations demonstrating that the Glu residue is linked at the γ-carbonyl to 2-thiohistidine, thus establishing thioglutine as a novel Glu–2-thiohistidine dipeptide natural product and the first modified TSI isolated from the original microbial host. HR-MS analysis confirmed that the enzymatic product is identical to the extracted natural product, validating the proposed biosynthetic pathway (Figure 5D).

### Characterization of Additional NHISS-ATP Grasp Operons

We next tackled additional subfamilies from the SSN (Figure 2). While the NHISSs in these additional subfamilies are localized on the OvoA-like branch (Figure S12), the imidazole binding contacts closely resemble that of an EgtB, suggesting they all generate a C–S bond at the 2-carbon of the imidazole side chain. We selected an NHISS that clusters with subfamily 8, derived from a metagenome assembled genome (MAG) from a Myxococcales strain, as well as one from a MAG derived from Sandaracinaceae from subfamily 9 (Figure 6A). These two NHISSs were obtained via recombinant expression of an *N*-terminal His-tagged construct and purified by immobilized metal affinity chromatography (IMAC). Upon incubation with Cys and His, we observed formation of 2-thiohistidine sulfoxide in both cases, as determined by HR-MS analysis (Table S7). We obtained a better 2-thiohistidine sulfoxide yield (>3-fold) with the Myxococcales enzyme, which allowed us to confirm the location of C–S linkage by ^1^H NMR (Figures 6B and S3,S4). Moreover, reaction with a C–S lyase (CsdA) *in vitro* resulted in 2-thiohistidine formation in both cases as determined by HR-MS analysis (Table S7). We have termed these enzymes TgiA-II, as they diverge significantly from TgiA from *S. varsoviensis* and are also involved in thioglutine formation (Figure 5B, see below).

**Figure 6:**
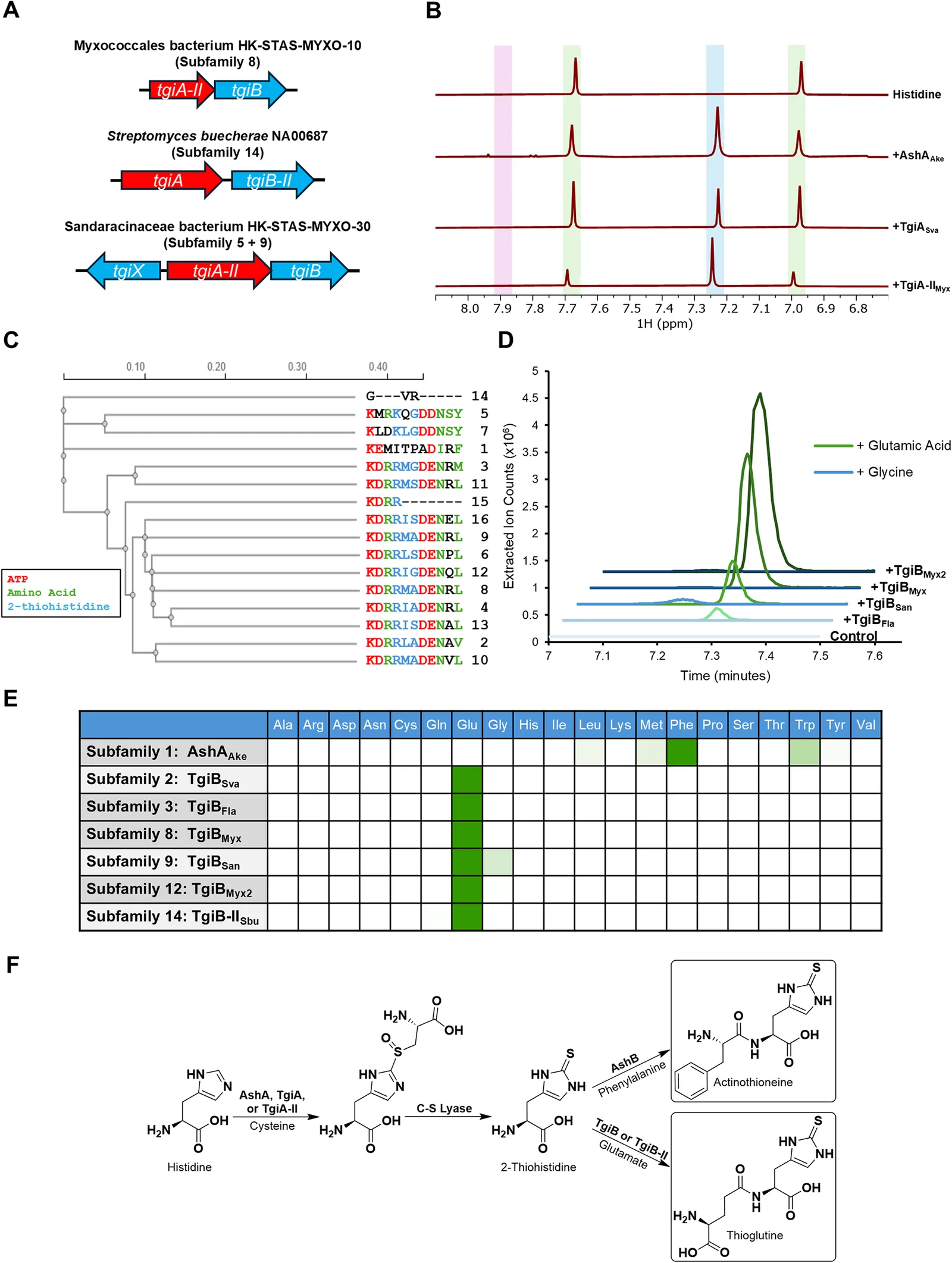
Characterization of additional ATP-grasps within the SSN. (A) Gene clusters chosen for further analysis. (B) ^1^H NMR analysis of TgiA-II reaction, focusing on aromatic protons, showing C–S bond at the 2-carbon of the imidazole side chain. The peaks associated with histidine’s side chain protons are highlighted in green; these correspond to the imidazole 5-^1^H (6.98 ppm) and 2-^1^H (7.68 ppm). The downfield ^1^H chemical shifts of the product of TgiA-II are identical to that of AshA and TgiA, highlighted in teal (7.23 ppm), which corresponds to the 5-^1^H of the sulfoxide side chain. The pink highlight at 7.9 ppm corresponds to the 2-^1^H in the 5-thiohistidine sulfoxide side chain, which carries a sulfoxide at the 5-carbon. Full structures of sulfoxides are shown in Figure S3. (C) Phylogenetic tree composed of a representative member of each ATP-grasp subfamily and its corresponding composite motif, demonstrating that these are fairly divergent. Residues in red align with ATP-binding residues. Those in blue likely coordinate the backbone of 2-thiohistidine, while residues in green are thought to coordinate the *C-*terminal amino acid. (D) Reaction of four additional ATP-grasp enzymes with all 20 canonical amino acids. All exhibit a significant preference for glutamate over glycine. (E) Heat map showing the preference of seven ATP-grasp enzymes when tested with all 20 canonical amino acids, again demonstrating a preference for Glu and a small degree of turnover with Gly (with the exception of AshA_Ake_). (F) Proposed biosynthetic pathway for the new TSIs in the SSN.

All other ATP-grasp enzymes in our network showed significant homology to TgiB, the only exception being the single enzyme in subfamily 14, termed TgiB-II (Figure 6A). This unique ATP-grasp appears in a *S. buecherae* adjacent to a TgiA. While all other ATP-grasps in the network are RimK-like, this particular enzyme from subfamily 14 is the only one that resembles YbdK, a glutamate-cysteine ligase.^35^ The composite motifs from the enzymes in subfamilies 5 and 7, both termed TgiX (for TgiB extra as they occur only with another ATP-grasp enzyme), showed differences in the substrate side chain binding motif (Figure 6C). We selected ATP-grasp enzymes from subfamilies 3, 4, 6, 7, 8, 9, 12, 14, and 16 for *in vitro* analysis (Figure 2). Unfortunately, the TgiX from subfamily 7 and TgiB from from subfamilies 4, 6, and 16 were not soluble in *E. coli* recombinant expression attempts or expressed poorly. We did, however, obtain soluble TgiB from subfamilies 3, 8, 9, 12, and 14 (Figure 6C). Reaction of these with 2-thiohistidine and all 20 canonical amino acids all resulted in formation of thioglutine (Figure 6D,E and S13). A small amount of the Gly–2-thiohistidine dipeptide was observed with the enzyme from subfamily 9. The yield was >4-fold lower than thioglutine (Figure 6E). Moreover, reaction of these enzymes with 5-thiohistidine with a mix of 20 amino acids did not result in any product formation, further highlighting the preference for these ATP-grasp enzymes for 2-thiohistidine (Figure S7). These results show that nearly half of the network is involved in thioglutine formation with subfamily 1, which generates actinothioneine, marking a key exception (Figure 6F). Unfortunately, subfamilies 3–17 are derived from MAG sequences, with 14 being the only exception; we were unable to acquire the bacterium encoding subfamily 14 (*Streptomyces buecherae*). The natural product from this host remains to be determined. Overall, the mature products from these pathways, notably those that encode two ATP-grasp enzymes, remain to be identified and provide incentive for future research with the goal of further expanding the TSI family of natural products.

## Conclusion

We set out to assess whether the TSI chemical space is wider than previously known. Using a simple bioinformatic approach wherein we searched for biosynthetic enzymes adjacent to an NHISS, we found hundreds of uncharacterized gene clusters that will form the basis for future discovery efforts. Among the racemases, acyltransferases, and ATP-grasp enzymes that co-occur with NHISSs, we focused on the latter group and generated an SSN of NHISS–ATP-grasps. The main finding from this work is that 2-thiohistidine is a genetically encoded building block that can be incorporated into dipeptides. Specifically, we find that the basic dual gene arrangement of an NHISS and an ATP-grasp enzyme gives rise to unusual dipeptides that incorporate Phe or Glu as the *N*-terminal and 2-thiohistidine as the *C*-terminal residue, resulting in actinothioneine and thioglutine, respectively, the latter of which contains a γ-carbonyl-linked Glu residue. There are several intriguing aspects about this new class of dipeptides. All approximately hundred arrangements of NHISS–ATP-grasps give rise to either actinothioneine, thioglutine, or thioglutine-based products with enzyme phylogenies ranging from very similar to significantly disparate, suggesting convergent functional evolution toward these natural products. In about half of the BGCs, these appear to be the final products, while several pathways may modify the dipeptides further. Notably, some BGCs encode two ATP-grasp enzymes and the function of the TgiX in these cases remains to be determined, providing fertile ground for future research. The ATP-grasps contain a previously uncharacterized motif, consistent with recognition of a new residue, 2-thiohistidine, for this enzyme family. The discovery of these enzymes should aid future biocatalytic applications in the synthesis of alternative products and the design of enzymes that incorporate analogs of 2-thiohistidine. These observations also motivate studies to determine the biological functions of actinothioneine and thioglutine. Interestingly, the concept of 2-thiohistidine-containing short peptides has been explored in a synthetic context, where they were found to display unexpectedly strong antioxidant, radical scavenging, and copper binding properties.^36^ These results provide an excellent starting point for functional assays of actinothioneine and thioglutine.

To date, enzyme-catalyzed TSI-tailoring reactions have been exceedingly rare. To the best of our knowledge, the only other example is found within the lincomycin biosynthetic pathway, wherein the glycosyltransferase LmbT catalyzes nucleophilic thiol addition of ergothioneine into the anomeric position of a GDP-sugar.^37,38^ However, the ergothioneine moiety is immediately displaced by mycothiol in the next step of the pathway, releasing the TSI in an unmodified form. Therefore, our discovery of ATP-grasp-associated NHISS enzymes represents a new biosynthetic paradigm in which the TSI moiety remains a key structural feature in the final products.

We explored the structural basis for regioselectivity of sulfoxide formation at the 2-carbon in this work. Interestingly, the NHISS enzymes in our network all occur on the branch dominated by enzymes that drive chemistry at the imidazole 5-carbon; however, all newly identified NHISSs generate a sulfoxide at the 2-carbon. The basis for regioselectivity lies in an alternative binding mode of histidine, which orients the 2-carbon of the side chain toward the key active site Tyr residue. These results are in line with the previously proposed role for this conserved Tyr residue in determining the regiochemistry of carbon-sulfoxide bond formation.^13,14^ Aside from exploring TSI building blocks in other biosynthetic pathways, e.g., in nonribosomal peptides, future efforts will also focus on the exact mechanism of sulfoxide and selenoxide formation by NHISS enzymes.

## Supplementary Material



## Notes

Complete materials and methods; gene and protein sequences of the enzymes studied herein; bacterial strains, primers, and plasmids used/generated; HR-MS and 1D/2D NMR spectra of natural products and biosynthetic intermediates and compounds characterized in this work; HPLC-MS and NMR spectral data for the reactions of ATP-grasp and NHISS enzymes; X-ray crystallographic analysis of AshA and refinement statistics; and bioinformatic analyses (PDF)

## ⊥

The Rockefeller University, New York, New York 10065, United States

## #

Perelman School of Medicine, University of Pennsylvania, Philadelphia, Pennsylvania 19104, United States
