# Supplementary material for: Widespread Biosynthesis of Thiohistidine Dipeptides in Bacteria
Source: J Am Chem Soc. 2026 Oct 1;148(40):42939–48. doi: 10.1021/jacs.6c03714 (PMC13674229; doi:10.1021/jacs.6c03714)
Supplement: Supplementary file 1 [file ja6c03714_si_001.pdf]

Supplementary Information for

Widespread Biosynthesis of Thiohistidine Dipeptides in Bacteria

Marissa L. Abbott,[1] Jacob T. Leeman,[1] Chase M. Kayrouz,[1] Kendra A. Ireland,[2] Katherine M. Davis,[2] and Mohammad R. Seyedsayamdost[1,3,*]

[1]Department of Chemistry, Princeton University, Princeton, NJ 08544, United States

[2]Department of Chemistry, Emory University, Atlanta, GA 30322, United States

[3]Department of Molecular Biology, Princeton University, Princeton, NJ 08544, United States

[*]Email: mrseyed@princeton.edu

MATERIALS AND METHODS

Materials. All reagents used were purchased from Sigma-Aldrich or ThermoFisher unless otherwise indicated. Molecular Biology and cloning reagents and strains were purchased from New England Biolabs. Primers were purchased from Azenta Genewiz. Codon-optimized gene blocks were purchased from Twist Bioscience.

General Experimental Procedures. High-performance liquid chromatography (HPLC)-coupled high resolution mass spectrometry (HR-MS) was conducted on either an Agilent 6540 or an Agilent 6546 quadruple time of flight (QtoF) system. These systems consist of an autosampler, a diode array detector, a JetStream electrospray ionizer and a 6540 or 6546 QtoF. Compounds were purified on Agilent 1260 HPLC systems equipped with an autosampler, a diode array detector, and a fraction collector. HR-MS data were analyzed through Agilent MassHunter Qualitative Analysis 10.0. NMR spectra were taken on a Bruker Avance 500 MHz spectrometer with a TCI cryoprobe or a Bruker Avance III 800 MHz NMR operated with Bruker TopSpin software. Spectra were analyzed with Mestrelab Research's MNova software.

Bacterial Strains and Culture. *Streptomyces varsoviensis* was maintained on ISP2 media at 250 RPM at 30°C. *Streptomyces albus* TK64 was maintained on SFM agar and a combination of TSB and ISP4 broth at 250 RPM and 30 °C. All *Amycolatopsis* strains were maintained on ISP2 agar and a combination of ISP2 and ISP4 broth at 200 RPM and 30 °C. *Escherichia coli* were maintained on LB media at 200 RPM and 37°C.

Plasmid Construction for Protein Expression. Synthetic DNA fragments (Twist Biosciences) were obtained with overhangs to facilitate assembly of plasmids. pET28b(+) and pET15b(+) were linearized using restriction enzymes NdeI and XhoI (NEB), incubated with DNA fragments, and assembled with HiFi DNA Assembly Master Mix according to the manufacturer's instructions. Plasmids were transformed into chemically competent *E. coli* DH5α via heat shock and plated on LB agar plates containing 50 mg/L kanamycin for pET28b(+) or 100 mg/L ampicillin for pET15b(+). Colonies were used to inoculate 5 mL of overnight cultures. Plasmids were recovered using the Qiagen Miniprep Kit. Upon confirmation by Sanger sequencing (Azenta Genewiz), plasmids were transformed into chemically competent *E. coli* BL21 (DE3) cells.

Protein Expression and Purification. Large-scale production cultures were inoculated with 1% overnight culture with 50 mg/L kanamycin or 100 mg/L ampicillin and grown at 37 °C/200 RPM. Protein expression was induced at OD_{600} of 0.5–0.6 after cooling to 18 °C with a concentration of 0.2 mM isopropylthiogalactoside (IPTG). Cells were pelleted at 10,000 x g and stored at -80 °C until protein purification.

All purification steps were carried out at 4 °C. Cell pellets were resuspended in 5 mL lysis buffer (25 mM Tris, 500 mM NaCl, 10 mM imidazole, 10% glycerol, pH 8) per gram of cell pellet supplemented with DNase, lysozyme, and 25 µL 100 mM phenylmethylsulfonylfluoride, which was prepared as a stock in isopropanol, per gram pellet. Cells were stirred on ice for 20 minutes before being sonicated thrice (15 second on/off cycle for a total of 2 minutes sonication time, 30% power, ~ 150 W). Cell lysate was clarified by centrifugation at 12,000 x g for 75 min. The lysate was loaded onto a 7 mL Ni-NTA column and washed with 40 mL of wash buffer (25 mM Tris, 500 mM NaCl, 30 mM

imidazole, 10% glycerol, pH 8). Protein was then eluted with 40 mL of elution buffer (25 mM Tris, 500 mM NaCl, 300 mM imidazole, 10% glycerol, pH 8) and desalted on a 75 mL Sephadex G-25 column into storage buffer (50 mM Tris, 150 mM NaCl, pH 8). The purified protein was concentrated using a 15 mL Amicon® Ultra Centrifugal Filter. Final concentrations were determined using absorbance at 280 nm, with the extinction coefficient calculated by Expasy, before flash-freezing the samples and storing at -80 °C.

Bioinformatics. A database of over 5,000 OvoA-like NHISS enzymes was aligned using command-line MAFFT software.[1] Sequences were ordered with command-line FastTree[2] and imported into FigTree (https://tree.bio.ed.ac.uk/software/figtree/) for visualization.

***In Vitro* Assays for NHISS Activity.** Enzyme activity assays were conducted on a 700 µL scale in Reaction Buffer (50 mM Tris, pH 8, 4 mM dithiothreitol (DTT)) supplemented with 2 mM sodium ascorbate, 1 mM cysteine, and 1 mM of either histidine or hercynine. Reactions were initiated with the addition of 10 µM purified NHISS. After 7 hours, reactions were quenched with 30 kDa Amicon® spin filters. 600 µL were lyophilized and redissolved in 120 µL D_2O. Samples were centrifuged and transferred to 2.5 mm NMR tubes, where ^{1}H-NMR spectra were recorded (typically 128 scans).

***In Vitro* Assays for ATP-Grasp Selectivity.** Substrate competition reactions were carried out on a 100 µL scale in Reaction Buffer (see previous paragraph) with 2 mM adenosine triphosphate (ATP), 5 mM magnesium sulfate, and 1 mM 2- or 5-thiohistidine. Each canonical amino acid was supplied at a final concentration of 0.5 mM. The enzyme was added last (at a final concentration of 10 µM) to initiate the reaction. After 2.5 h of incubation at room temperature, reactions were quenched with 10 kDa Amicon® spin filters. The products were derivatized for 0.5 h with an equal volume of 10 mM monobromobimane (mBBr) in acetonitrile (ACN). Samples were filtered and analyzed through UPLC-QtoF-MS with a Synergi Hydro-RP Column (Phenomenex, 250 × 4.6 mm, 4 µm) and a mobile phase consisting of water and ACN, each containing 0.1% formic acid. Samples were eluted with 5% ACN for 3 min followed by a gradient from 5–95% ACN over 9 min. Data were analyzed through Agilent MassHunter Qualitative Analysis 10.0. Assays with $TgiB_{Sva}$ were carried out with identical conditions, except that the reaction was allowed to proceed for 24 h.

The selectivity of AshB for leucine was confirmed through 100 µL assays in Reaction Buffer containing 2 mM ATP, 5 mM magnesium sulfate, and 1 mM 2-thiohistidine. Reactions were initiated with addition of 1 mM isoleucine or leucine. After 75 min, the reactions were quenched with 10 kDa Amicon® spin filters. Products were derivatized for 0.5 h with an equal volume of 10 mM mBBr in ACN. Samples were filtered and analyzed through UPLC-QtoF-MS with a Synergi Hydro-RP Column (Phenomenex, 250 × 4.6 mm, 4 µm) and a mobile phase consisting of water and ACN, each containing 0.1% formic acid. Samples were eluted with 5% ACN for 3 min followed by a gradient of 5–95% ACN over 9 min. Data were analyzed through Agilent MassHunter Qualitative Analysis 10.0 by extracting the ion count at m/z = 491.2071.

Enzymatic Synthesis and Characterization of Actinothioneine. Actinothioneine was enzymatically synthesized on a 60 mL scale in Reaction Buffer with 5 mM

magnesium sulfate, 2 mM ATP, and 1 mM of both 2-thiohistidine and phenylalanine. The reaction was initiated by adding AshB dropwise to a final concentration of 10 μM and left to react overnight. The reaction was quenched by enzyme removal with Amicon® 10 kDa spin filters (15 mL capacity). The resulting solution was lyophilized, then dissolved into 10 mL of 20% methanol in Milli-Q water supplemented with 0.25% formic acid. This was then fractionated with preparatory HPLC on a Luna Omega Polar column (Phenomenex, 250 × 10 mm, 5 μm) at a flow rate of 15 mL/min with a mobile phase consisting of water and ACN, each containing 0.1% formic acid. The sample was eluted with 5% ACN for 3 min followed by a gradient to 100% ACN over 8 min. Fractions containing actinothioneine were flash frozen and lyophilized. The resulting powder was dissolved into 6 mL water and subjected to semipreparatory HPLC on a Luna Omega Polar column (Phenomenex, 150 × 21.2 mm, 5 μm) using an isocratic elution of 2% ACN in water (+0.1% formic acid) for 30 min. The pure fractions, as assessed by NMR, were collected and combined, yielding 6 mg of material (at >95% purity). The compound structure was determined via ^{1}H, COSY, HSQC, and HMBC NMR spectra. Future enzymatic syntheses of actinothioneine should consider using tris(2-carboxylethyl)phosphine as a reductant in place of DTT to simplify the purification process.

Heterologous Expression of *ash* in *E. coli* and Coinjection with Actinothioneine Standard. pET28b(+)_6xHis-AshA$_{Ake}$, pET15b(+)_6xHis-AshB$_{Ake}$, and pGro7 were cotransformed into chemically competent *E. coli* BL21 (DE3). A control strain was transformed with empty pET28b(+), empty pET15b(+), and pGro7. Colonies were used to inoculate a 5 mL starter culture supplemented with 50 mg/L kanamycin, 25 mg/L chloramphenicol, and 100 mg/L ampicillin. After overnight growth, the starter was used to create a 1% inoculum for duplicate 25 mL cultures. These cultures were supplemented with the aforementioned concentrations of antibiotics as well as 2 g/L arabinose to induce GroEL/GroES expression. After 2.5 h of growth at 37°C, cultures were iced and induced with 0.2 mM IPTG. After 21 h of expression, the cells were pelleted by centrifugation for 5 min at 4,000 x g. The pellets were extracted with 150 μL of 10 mM mBBr in ACN and sonicated for 20 min. The samples were filtered and analyzed by UPLC-QToF-MS with a Synergi Hydro-RP Column (Phenomenex, 250 × 4.6 mm, 4 μm) and isocratic elution with 5% ACN in water (+0.1% formic acid) followed by a gradient of 5–95% ACN over 9 min. Data were analyzed through Agilent MassHunter Qualitative Analysis 10.0 by extracting the ion counts of all compounds associated with the *ash* pathway.

A standard of mBBr–actinothioneine was generated by mixing equal volumes of 10 mM actinothioneine in water and 10 mM mBBr in ACN. The resulting mixture was filtered and diluted by a factor of 1200 to match the counts in the *E. coli* expression system. A 1:1 mixture was then generated and all three samples subjected to the same UPLC-QToF-MS method described above. Data were analyzed through Agilent MassHunter Qualitative Analysis 10.0 by extracting the ion count at m/z = 525.1915.

Crystallization of AshA. Crystals of AshA were grown using the sitting drop vapor diffusion method at room temperature. To obtain crystals bound to histidine, 2 molar eq. of $(NH_4)_2Fe(SO_4)_2$ and 30 mM histidine were added to 6 mg/mL AshA, which was then mixed 1:1 with a precipitant solution of 0.1 M Tris pH 7, 0.2 M lithium sulfate monohydrate,

2 M ammonium sulfate, and 3% ethylene glycol. Polyhedral crystals appeared after two months. For harvesting, crystals were looped and briefly transferred into cryoprotectant before flash freezing in liquid N_2. Cryoprotectant was comprised of the precipitant with 20% (v/v) ethylene glycol and 30 mM histidine.

X-ray Data Collection and Processing. Crystals were maintained at 100 K to minimize X-ray-induced damage during data collection. The data were collected sequentially ($\Delta\varphi$ = 0.1°) with an incident wavelength of 0.9202 Å at beamline 17-ID-1 of the National Synchrotron Light Source II using an Eiger 9M (Dectris) detector. Data were indexed, integrated, and scaled with XDS before merging with AIMLESS.[3,4] The structure was solved via molecular replacement with Phaser, using the AlphaFold3-predicted structure as the search model after removing all residues with per-residue confidence scores (pLDDT) below 90%.[5] Model building was conducted in Coot,[6] the structure refined in Phenix[7], and model quality assessed using Molprobity.[8] Data processing and refinement statistics can be found in **Table S6**. The structure is in the orthorhombic P 2_1 2_1 2_1 space group and contains one molecule in the asymmetric unit. Figures depicting the structures were generated with PyMOL.[9]

Isolation of Thioglutine from *Streptomyces varsoviensis* Through mBBr Derivatization. *Streptomyces varsoviensis* was cultivated in ISP2 medium (11 L total) at 30°C with shaking at 250 RPM. After 7 days, the cultures were pelleted at 8000 x g for 5 min, yielding an 87 g cell pellet. The supernatant was discarded and the pellet resuspended in 220 mL of methanol. The suspension was sonicated in a bath sonicator for 30 min, then pelleted at 10,000 x g for 10 min. The supernatant was collected, and the extraction was repeated twice more with fresh methanol. The pooled extracts were concentrated by rotary evaporation, yielding a yellow oil, which was then frozen and lyophilized to afford 314 mg of crude extract.

The crude powder was redissolved in 12 mL of 1:1 water/methanol, and 16 mg of mBBr was added. The mixture was completely dissolved and sonicated for 20 minutes. Fractionation was performed using preparative HPLC with a Luna Omega Polar column (Phenomenex, 250 × 10 mm, 5 µm) with isocratic elution (5% ACN in water, 0.1% formic acid). Fractions containing the target compound were pooled and lyophilized.

The material was redissolved in 1:1 water/methanol and subjected to further fractionation using a Synergi Hydro-RP column (Phenomenex, 250 × 4.6 mm, 4 µm) with the same 5% isocratic gradient described above. Fractions containing thioglutine were pooled and lyophilized, yielding 3 mg of semi-pure material, which was dissolved in D_2O and characterized by NOESY, COSY, HSQC, and HMBC NMR spectroscopy. The sample was then lyophilized and further purified by HPLC using a Luna Phenyl-Hexyl column (Phenomenex, 250 × 4.6 mm, 5 µm) with isocratic elution consisting of 5% ACN in water, 0.1% formic acid followed by an 8% isocratic elution to yield <1 mg of pure material, which was characterized by ^{1}H NMR spectroscopy.

Coding DNA Sequences and Amino Acid Sequences of Recombinant Proteins

<u>6xHis-*ashA*$_{\mathbf{Ake}}$ CDS (from *Amycolatopsis keratiniphila* subsp. *keratiniphila*, synthesized, codon optimized)</u>

ATGGGCAGCAGCCATCATCATCATCATCACAGCAGCGGCCTGGTGCCGCGCGGC
AGCCATATGACTTCTACCTCTACTTTAGAACCTTGGGAACGAAATGCGGTCAAGCA
TGCCGATGAGAGACTTAACGGTGCACGCGATCCTCAGTGGTGGTATACCGGCGTA
CATCCGGTACCGGGACGCTGTCCCGGAGTTGCAGAGTCCGGTGAGATTCGTGCTC
TGCCTCTGCCTGATTTAAGCGTCTGCTCCCGGGAAGAGGTTCTGGATTACTTTGAC
AATGCGTGGACCCTGCATGAAGTGTTATTTGCGGGTCTTAAGGGTGCAGAAGCCTT
TTACAGACCGCCCGATCATAATCTGAGACATCCAATGATTTTTTATTATGGTCATAC
TACTGCACTCTACGTTAACAAGTTGCGCGTTAGTGGGCTGCTGGACGCCCCGATT
GACGCATATTTAGAACATGTCCTGGAAATGGGGGTTGATGAGAACAGTTGGGACG
ACATGTCTAAAAATGACATGGTGTGGCCGTCCGTTTCTGAAGTGAAAGATTATCGC
GCAGAGGTTTACCGGACTGTGGTAGGGATCGTAGAGCGTACCCCGTTTGCCCCAG
ATCTTGGGCGCCCAGTTACAATGGACGATCAGGCGTGGGCCCTGCTCTTAGCAAT
CGAGCATGATCGCATACATCTGGAAACTTCTAGTGTACTGATTCGCGAAATGCGCC
CGTCCCTTGTAGAGCGCCCTGCCGCTTGGCCAGCGCCATCACCTTTAAGAAGAAC
CAGTGAAACCGGGAAGCCGGAACCTGGCAGTGTACCGGCCAACGATACTGTGAC
AGTTCCTGGACGTACCGCCCGACTGGGCAAACCGCAGTCTTTTCCGTCATATGGA
TGGGATAGTGCGTACGGTAGTCGTCGTCAGGAACTGCCCGACTTCGAAGCGGCC
CGCTGTTTGGTTTCTAATGGCGAATTCCATGGCTTTGTTGCGGATGGAGGATATCG
TCGTCCGGAATTATGGTCTGCAGAGGGGGAAGCATGGCGTAGATTTCGTAATACA
AAGTGGCCACGTTTCTGGGTCCCGACTGGTCCCACAGGTCTGCATGCATTCCGTC
TGCGTACTCTCTTCGATGAGATAGATATGCCTTGGGATTGGCCGGTAGTTGTAAAT
TATCATGAAGCGAAAGCTTTTGCCGCCTGGCGTTCGGAAAAAGATGGTCGCGAAT
ATCGTTTACCTACTGAGGCGGAATATTTCTTACTTCGCGATCTTCCCGAGCGTCCG
GGCGTGGCAGATGACGTCGTAATGCGCCTGGACGGCGCGCAGCTGCGTGAAACC
GGGATTAACCTGGGCCTTGCGTGGGGGTCCGAAGGTCCCGTGGATCACTCCCCG
ACCACGTCACAGGGGTTACATGACGCTGGTGGGAATCTGTGGGCATGGTCAGAAG
ACACCTTCAACCCATTGGATGGGTTTACTCCGCATCCTTATTATGAAGATTTTTCGG
TACCTAGTTATGGGGGACAACACCAACTGATTCTTGGAGGCGCCTACATTTCGACA
GGGGAGCTGGGATCTATATGGGCACGTCACTTCTACCGCCCGCATTTTCTGCAAC
AAGCCGGCATTCGCCTGGTGGTGGCTCCAGAGAGTACCGAGGATGGTGCAGCAA
GAACAGAATTAGATAGAACGCTGCTGTCTCAATATGGTACCGCGGAGCAGGTCTTT
GGCCGCGCAGGTCATCCCTTGGCCGCAACCGTGGGTCATCCAGATCGCCTGGCC
CGTAGATTTTTTGAAGCCGCAGATGAAGCCGGGGTGGAGCTTCGTCGCGTCGCCG
ACCTGGCAACCGCTGCAGGAGGCTTAGCCTTCTCTCTCGCTGGTCGACCAATTGA
AGTTGTAGGCTTGGATGATCGTCCGGCCTTTATTGCGGCCGCTGATCGGTTGGCT
GAAGGATGGGACGTAGGCTATCGAGTACCGGGTCGAGGGGAACCCCATGCCAGA
CGCCCAAGCATAGCGCCCGGCGGTGGCGTTAGCTTCACCCTTGCCGAAGCTAGA
AGACCCCCAGCAGCCCTGGGTACCTTCGATGGAGTTGTCGTTGCCGATGCTTTTG
ATCGGCTCGGGGCGACCGGATATCTGCGTCCGCTGGCTGGCTCTGAAAATTTTTT
GCGTGCAGGGGGACTTTTATTAGTAGCCCTTCCGCGGGCGTCAGCCGCGGAACT

GGCAAATGTCCTGGGGGAAGTATTTGAATTGCTCAGTGAAGACACCGAACCAAGC
CTTACAAATGCGGATGGTCACCGTTATGAAATTACAGACCTGGAGGTTTCCGTGTG
GAGAAAACGGTAA

6xHis-AshA$_{Ake}$ protein sequence

NCBI Identifier: WP_063272911.1

MGSSHHHHHHSSGLVPRGSHMTSTSTLEPWERNAVKHADERLNGARDPQWWYTGV
HPVPGRCPGVAESGEIRALPLPDLSVCSREEVLDYFDNAWTLHEVLFAGLKGAEAFYR
PPDHNLRHPMIFYYGHTTALYVNKLRVSGLLDAPIDAYLEHVLEMGVDENSWDDMSKN
DMVWPSVSEVKDYRAEVYRTVVGIVERTPFAPDLGRPVTMDDQAWALLLAIEHDRIHL
ETSSVLIREMRPSLVERPAAWPAPSPLRRTSETGKPEPGSVPANDTVTVPGRTARLGK
PQSFPSYGWDSAYGSRRQELPDFEAARCLVSNGEFHGFVADGGYRRPELWSAEGEA
WRRFRNTKWPRFWVPTGPTGLHAFRLRTLFDEIDMPWDWPVVVNYHEAKAFAAWRS
EKDGREYRLPTEAEYFLLRDLPERPGVADDVVMRLDGAQLRETGINLGLAWGSEGPV
DHSPTTSQGLHDAGGNLWAWSEDTFNPLDGFTPHPYYEDFSVPSYGGQHQLILGGA
YISTGELGSIWARHFYRPHFLQQAGIRLVVAPESTEDGAARTELDRTLLSQYGTAEQVF
GRAGHPLAATVGHPDRLARRFFEAADEAGVELRRVADLATAAGGLAFSLAGRPIEVVG
LDDRPAFIAAADRLAEGWDVGYRVPGRGEPHARRPSIAPGGGVSFTLAEARRPPAAL
GTFDGVVVADAFDRLGATGYLRPLAGSENFLRAGGLLLVALPRASAAELANVLGEVFE
LLSEDTEPSLTNADGHRYEITDLEVSVWRKR

6xHis-*ashB*$_{Ake}$ CDS (from *Amycolatopsis keratiniphila* subsp. *keratiniphila*, synthesized, codon optimized)

ATGGGCAGCAGCCATCATCATCATCATCACAGCAGCGGCCTGGTGCCGCGCGGC
AGCCATATGTCCACAAAACCTTGCGTGGTGTTGGTTGATACCTATGAAACGGCTGA
CCATGGATTACCACAAACAGCTCGCCATATCGCACCTGCCTTTTTGGCACTGGGAG
CGGAATGCGTACGAGTGCAATCTGCCCCAGACGCACCAGGTCAATATCGAAAATC
CAGTCCGACCATGTCAAACTACCGAGAGAATATTGTACATGATGGTGATTTGGCTA
AAACCCTCGATGCCTTAGCTGTACATCGCCCTGTGGCAGTGGTAGCTGGTGGTGA
GTATGCGGTGGCGTTTACCGATGAGCTGTCCGAGGCACTTGGTGTTCCGAGTAAT
GGGACTGCACTTTCACTTGCTCGTCGTGATAAAGCCGCTATGGGTGAGGCAGTTG
CTGCGGCGGGATTACCGGTCGCCCGTCAGTTGAGTACTTCCGACGAGGCAGAATT
AGCGGCATGGCATCAAGAAGTTGGTGGCCGTATCGTGGTTAAACCAAACCGCTCC
GCCGGGGGCGATGGTGTTTCTTTTTGCGACACCCCCGAACGTAGCGTCGAAGCCC
TGCGCACCATCACCGGTCGTCAGAACGTGTTTTCAGAAGACAATGATTTAGTAATT
GCTCAAGAATATCTTGCGGGTACAGAGTATATGATAAATACCGTCAGCATGGATGG
TCTTCATCAGGTGTGCGACATATGGAAAACGAGCCGGTTTGCGATTAATGGCGTGT
CCGACCTGCTGGGAGCATGTCAGATTGTGCCGCGGAGAGGTCCTGGACATGACG
AACTGGTTAACTACGCGTTCGCGGTGCTGGATGCCCTGGGTATTCGCCACGGTGC
CGCGCACGTCGAGATCAAACGGACGCCTCAGGGGCCTAGACTGATTGAGGTGGG
CTCGCGGCTTTCAGGGGGAGATTTGCCTCACTTGGCTCGTTTGGCTATTGGTGAG
TCACAGCTCGATTGGATGGCGGATGCCGTTCTGCGTCCGGAACGTTTTAGAGAGC

GGTGGGGTGAGGACTATAGAATTCGACGTCATTACGCATGGTCAGCCCTTCTGAG
CCCCGTGGAAGGCACACTTCGTCGTTATACCGTTCTTGATGAAATCGAACGCTTAG
AATCATTCCATGAGATGTTCGTACTGACCCAGCCAGGTAAACCCTTGAGACGTACA
GTTGATGATACAACATTCCCGGTTGTCGTTACCTTTATGCACGAAATTGAAGAGTAT
GTTCTGCGAGATCTTGGAACCCTGAGATATCTGGACGGAGTCGGGTGTTATGAGTT
GGAACCTTAA

6xHis-AshB$_{Ake}$ protein sequence
NCBI Identifier: WP_063272941.1
MGSSHHHHHHSSGLVPRGSHMSTKPCVVLVDTYETADHGLPQTARHIAPAFLALGAE
CVRVQSAPDAPGQYRKSSPTMSNYRENIVHDGDLAKTLDALAVHRPVAVVAGGEYAV
AFTDELSEALGVPSNGTALSLARRDKAAMGEAVAAAGLPVARQLSTSDEAELAAWHQ
EVGGRIVVKPNRSAGGDGVSFCDTPERSVEALRTITGRQNVFSEDNDLVIAQEYLAGT
EYMINTVSMDGLHQVCDIWKTSRFAINGVSDLLGACQIVPRRGPGHDELVNYAFAVLD
ALGIRHGAAHVEIKRTPQGPRLIEVGSRLSGGDLPHLARLAIGESQLDWMADAVLRPE
RFRERWGEDYRIRRHYAWSALLSPVEGTLRRYTVLDEIERLESFHEMFVLTQPGKPLR
RTVDDTTFPVVVTFMHEIEEYVLRDLGTLRYLDGVGCYELEP

6xHis-*csdA$_{Nos}$* CDS (from *Nostoc sp.* PCC 7120, synthesized, codon optimized)
ATGGGCAGCAGCCATCATCATCATCATCACAGCAGCGGCCTGGTGCCGCGCGGC
AGCCATATGACTTCCATCTCGACTCTGCAGACCCGGCTGCATTCCCATCGAGAACA
GTTCCCTGCTCTGGCGAATAAAACATATTTTAATTACGGTGGTCAAGGTCCCATGC
CGCAGGGCGCCATGGATATTATAACACAGAGTCTGACCTATATACAGCAGATCGG
GCCGTTTGGTACTGAGGCCGGTAACTGGATCACGCGCCAGACCCAAGCGGTTCG
CGAGGCCATTGCCAGCGAGCTGAATGCATCTTCTGATACTATAACGTTTACTGATA
ATGTCACTGTTGGGTGTAACATTGCCCTGTGGGGAATTCCTTGGCAGGCAGGGGA
TCATATTTTACTTTCGGATTGTGAACATCCGGGCGTGATTGCGGCGACCCAGGAAA
TTTCCCGCAGATTTGGTGTTGAAGTTACAACGTGCCCGTTAAAATCTACACTGAAC
GAGGGTGAACCGACAACGATTATCGCGCAGCATCTGCGAAAAAATACAAGACTTG
TGGTCCTTTCACATGTCTTTTGGAACACAGGTCAGGTTTTACCGTTGGACAAGATT
GTGGAAGTGTGTCGAAACAATAATTCTCTGTTACTTGTCGATGCGGCGCAGTCAGT
CGGTGCTCTGCCCCTGAATCTTACGGAATTGGGCGTAGATTTCTATGCATTTACCG
GACACAAATGGCTGTGCGGGCCCGAGGGGGTTGGTGGTCTCTATGTTAGACCTGA
AGTCCGTGAGTCGCTGAATCCAACCTTCATTGGTTTGTACGGAATCATTGTAGATA
ATCAGGCCCAGCCTGTATCTTGGAAGCCCGATGGTCGCAGATATGAAGTCAGTAC
ATTAGCTTATCCCTTATACTTAGGGCTCAAAGAAGCTATTGCCACACATCAGCGTTG
GGGTACTCCACAGGAACGTTACGCCCAGATTTGTCATAATTCCGAATACCTTTGGC
GGTCCCTGGCTGCGATTCCCAATGTGAAATGCTTACGAGATACCCCTCCTGAAACC
GGTATAGTATCATTTCAGCTGCATCAAACACCGTCGCTTAAGCTCGTACAGTTCCT
GGACTCGCAAAAGATCCTGACGCGTACTATTGCAGATCCTTCGTGCATCCGAGTAA

CCGTGCACTATCTCACCCTCGAAAGCGAAATTGATCAGTTAGTTGAAGCGATCGCT
AGATTCTCCGTTTAA

6xHis-CsdA$_{Nos}$ protein sequence

NCBI Identifier: WP_010997361.1

MGSSHHHHHHSSGLVPRGSHMTSISTLQTRLHSHREQFPALANKTYFNYGGQGPMP
QGAMDIITQSLTYIQQIGPFGTEAGNWITRQTQAVREAIASELNASSDTITFTDNVTVGC
NIALWGIPWQAGDHILLSDCEHPGVIAATQEISRRFGVEVTTCPLKSTLNEGEPTTIIAQ
HLRKNTRLVVLSHVFWNTGQVLPLDKIVEVCRNNNSLLLVDAAQSVGALPLNLTELGV
DFYAFTGHKWLCGPEGVGGLYVRPEVRESLNPTFIGLYGIIVDNQAQPVSWKPDGRR
YEVSTLAYPLYLGLKEAIATHQRWGTPQERYAQICHNSEYLWRSLAAIPNVKCLRDTPP
ETGIVSFQLHQTPSLKLVQFLDSQKILTRTIADPSCIRVTVHYLTLESEIDQLVEAIARFSV

6xHis-*tgiA*$_{Sva}$ CDS (from *Streptomyces varsoviensis*, synthesized, codon optimized)

ATGGGCAGCAGCCATCATCATCATCATCACAGCAGCGGCCTGGTGCCGCGCGGC
AGCCATATGGTGAACGCTTCTTCCGCCGCCCGGCCCGTAGCCCCGGCGCCTTTG
GGTGGCGGCTGGTCGAGGGACCGGCTCGCGCGCTACTTCGTGGACACGTGGGAT
ACGTACGAAACCCTCTTTCGCGGAGTCGCCGACTCGAAGGAGGCGTTCGGCGTC
CGCGTGCATCCGTTCAGGCTGCCTCTCGTCTTCTATCTCGGGCACACCGCGTCGT
TCTACATCCAGAAGCTCAAACTGGTCGGGATGGTGGACGGGGGCGGGGTCTGCG
AACTCGACGAGCGGATGGAGCAGGGGGTCTCCCCCGAGGACCCCGACGAATTGG
CCCGGCTCACCGAGTGGCCCGACCCGAATGATGTGTGGTCCTACCGGAAGACGG
TCCGCGACATCGTGTTGCGGACGATCGAGACCGTACCGCTGCCGGCGGTTCTCG
CCCCGACCTCGCCGCTGCGGGCCCTGCTCATGGGCATCGAGCACGAGAACATTC
ACCTGCACACGTCCCTCCCCCTCATTCGTCTGTTGCCGCCCAGGCTGAAAGGCAC
GCCCCAGGGCTGGCCCGCCATGGGAACGCAACGGGACGGAGGCGCGGTGGTGG
CGGAGCCCGGCAGGGATTACGTGCTCTGCCGGGGGCGGTTCGGTCGTGTTCGGGG
ACGATCAGCCGGAAACCGGCCGGTTCCGGTGGGACAACGAATACGGCCGCCGCG
CGGCGACGGTCGACCCCTTCGAGGTCGCGCCGCACCCGGTGACCAACGGCCAGT
TCCTGGAATTCGTCAGAGCCGGCGGCTATGAAGAGCCCGGCCTGTGGACGACGC
ACGGTGCGCGGCTGTATCTGGAGCGGGTGAGCGGAGGCCACCCCCATTCCTGGC
TGCCGGGGGCCGGTCCCGGCGGCTACCGGTATCGCGGCATCTTCGAGGACACGG
AAATGCCGTGGGACTGGCCGGTGGAGGTCAGCAGGCACGAGGCCGTGGCCTACG
CGAACTGGGCCGGCGCCTCACTCATCTCCGAGGAACAGTTCCACGCCCTCCTGGA
CCAGGAATTCGGCGGAGTGAGCCGGGTGGCGGCCATGCTCGACAAATTCGACGT
GGGCCTTCGCCGTGGCAGCCCGCGCCCGGTGCGCGGTCAGGGCGCGTTCCTCG
GCCGTAGCGGAATCGACTTCGTCGGGAACGTCGCGCTGTGGCTGGAGGACGATT
TCACAGCGTTGGAGCGGGGCTCCGATGACGCCGAGAGCTCCTTCCGCGTCGACC
CGCTGTATCCGGATTTCTCGGAACCGTGGTTCGGCCCAGAGACCGGCATGCTCAC

CGGCGCCAGTTACGCGGCGCGCGGCCACATGGCCGATGTCGGCGTCATGCGGG
ATTTCATGCAGAACCACATGGACCAGCTCGCCGGCATTCTCCTCACCAGGCCCGC
CCGATGA

<u>6xHis-TgiA$_{Sva}$ protein sequence</u>

NCBI Identifier: WP_078645114.1

MGSSHHHHHHSSGLVPRGSHMVNASSAARPVAPAPLGGGWSRDRLARYFVDTWDT
YETLFRGVADSKEAFGVRVHPFRLPLVFYLGHTASFYIQKLKLVGMVDGGGVCELDER
MEQGVSPEDPDELARLTEWPDPNDVWSYRKTVRDIVLRTIETVPLPAVLAPTSPLRALL
MGIEHENIHLHTSLPLIRLLPPRLKGTPQGWPAMGTQRDGGAVVAEPGRDYVLCRGG
SVVFGDDQPETGRFRWDNEYGRRAATVDPFEVAPHPVTNGQFLEFVRAGGYEEPGL
WTTHGARLYLERVSGGHPHSWLPGAGPGGYRYRGIFEDTEMPWDWPVEVSRHEAV
AYANWAGASLISEEQFHALLDQEFGGVSRVAAMLDKFDVGLRRGSPRPVRGQGAFLG
RSGIDFVGNVALWLEDDFTALERGSDDAESSFRVDPLYPDFSEPWFGPETGMLTGAS
YAARGHMADVGVMRDFMQNHMDQLAGILLTRPAR

<u>6xHis-*tgiB*$_{Sva}$ CDS (from *Streptomyces varsoviensis*, synthesized, codon optimized)</u>

ATGGGCAGCAGCCATCATCATCATCATCACAGCAGCGGCCTGGTGCCGCGCGGC
AGCCATATGCGGCAGGTGCATCTGATGCGCGCCGCACAGGAGTTAGGAATAGAAG
TGCATGACCGTATGGCTGAACTTGGTATCGATTTGGTAGTTTACCGCCACGGTACC
AAATCCATTCCGGTATTAGAAGGCCGTTATGATGACCAACTGCGCGCACGTGATTT
CTTTCTCACCGAAGACAAGCACGCAACTAAACAGCTGCTGCGTCGTGCAGGATTTA
CGACTCCAAAAGGCTGTGCCTTTGCATTCGATTGTGGACCTGAGGACACAGCCGA
GGGCATGGACGAAGGAATCTACGGAGAAGAAGCCGAGAAACTTGCTGAGGAGGC
CGAAGAAGCGGAAGAGGCGAAGGGACCAGAGAATCCTGAAGACCCTGAAGACGG
AAAGGACGGCGAAGATGGGGAATCGTCCTATGGTCGTGCACTTGCCTTTGCAGCT
GGCGCCCTGGCGGAATTAGATGGAGACCCTGTTGTAGTGAAGCCACGTGCCGGC
ATGCATGGTGATGCGGTCCGCATGGGGCTGGATTCGGCGGAAGCTGTAGCCGCC
CACATGAACACCTGGCGTCATCGTTTCCGAGATTGGATTGTAGAGGAAAGAGTGG
ACGGCCGCGACTTGCGCATTCAACTGATTGGAGGTGAATTGGTCGCAGCGTGCGT
CCGTGAACCAGCATCGGTGATAGGTGATGGTCAAACCCCTCTTGCAGAGCTGATA
AAAAAACACGATGCCGAGATTCGAGCTCTGAATCCTTCTAATCGTCTCACTGTGGA
TTCTGAGACGGAAGCGTTGTTGCATCGGCAAGGTCTGGAACCGGGCGACGCTCC
CGCGGCAGGCCGCGTTGTACGGCTGAAGAATACGGCAAATCTGGCAAAAGGTGG
CCGAGCAGTGGATGTAAGCGATTCTCTGCACCCGGGATTTCACGCCTGGGCCTCG
GCCATTTCACAACTTTTTGCAACGGATATTCTTGCCATCGATGCGATCTGTGCAGC
CCCGCACCGTCCGCCGGAGGGGAATGCTGCAATACTGGAGATTAATACAGCACCC
GAATGGGTACATCATACTTTTTCTGAACATCGCACACATGATATTGCTGCTCGTGTG
CTGCGACATTGGTTTGGATTGTGA

6xHis-TgiB$_{Sva}$ protein sequence

NCBI Identifier: WP_030886666.1

MGSSHHHHHHSSGLVPRGSHMRQVHLMRAAQELGIEVHDRMAELGIDLVVYRHGTK
SIPVLEGRYDDQLRARDFFLTEDKHATKQLLRRAGFTTPKGCAFAFDCGPEDTAEGMD
EGIYGEEAEKLAEEAEEAEEAKGPENPEDPEDGKDGEDGESSYGRALAFAAGALAEL
DGDPVVVKPRAGMHGDAVRMGLDSAEAVAAHMNTWRHRFRDWIVEERVDGRDLRI
QLIGGELVAACVREPASVIGDGQTPLAELIKKHDAEIRALNPSNRLTVDSETEALLHRQG
LEPGDAPAAGRVVRLKNTANLAKGGRAVDVSDSLHPGFHAWASAISQLFATDILAIDAI
CAAPHRPPEGNAAILEINTAPEWVHHTFSEHRTHDIAARVLRHWFGL

6xHis-*tgiA-II*$_{Myx}$ CDS (from Myxococcales bacterium, synthesized, codon optimized)

ATGGGCAGCAGCCATCATCATCATCATCACAGCAGCGGCCTGGTGCCGCGCGGC
AGCCATATGGACGTGATGCTGCCTCCGAGCCTGGCCGCCGATCGAGCCACGCTG
CTGGCCTACTTCGAGGAATCTTATCGTCTGTGTGACTGGCTGTTTTCGAGCGTCGC
CGAAGGCGCTCTTTATCAGCAGCCCGATCCTTTGAGACATCCACTGATATTTTATG
TTGGGCATCCGGCTGCTTTTTATATCAACAAGTTACGTCTTGCAGGCCTGCTGGAC
GCACCTGTGGACGCCGAGCTGGAGGTCCTGTTTGCAAAAGGTGTGGATCCACATG
CACCGGACGAACTGGACCGACGTGCCACTTGGCCTGCGTTGGATGTGGTGCGCG
CATACCGCGCTCGCGTGTTCGATGTTGTACGCGCAGTGATTGAAGCAACCCCGCT
GCCTCCGGTTGTTACATGGGATCACCCACTGTGGGCTCTTCATATGGCATGCGAA
CATGACCGTATACATTTCGAGACTAGCTCAGTACTGATTCGGCAGTTACCGGTTTC
ACAAGTGAGAGCGCCAGCTGGCTGGGCAGACGGACCGGTTGGCGGTGAACCGCC
GGCGCTGGCGTGGCGCTCGGTCCCTCGTCGCGCTGTTCAGTTAGGGCGGCCTCC
AGTGTCAGACGCCTATGGCTGGGGCAACGAATATGGAACTCTTGAAGTGGACGTT
CCACCTTTTCTGGCAACAACAACCCTGGTGTCCAACGCCGATTATGCGCGCTTTGT
CGCAGATGGCGGATACCGTAGACCTGAGCTGTGGACGAAAGCGGGCTGGGCCAG
CCTGGGGGGCGCAACTCATCCGCGTTTTTGGGTACCTGAAGCTGGTGGAGGGTTC
AGTTACAGAGCACAGTTCGATGTTCGCCCGTTGCCGCGGGCTTGGCCCGCAGAG
GTAAATGGTCATGAAGCAGCTGCCTACGCCCGTTGGGCTGGGGCAAGACTTCCCA
CAGAGCAGGAATGGGCGGCGCTCGCGGCAGACGCACCAACCCACGATGCAGATG
TTATCGGAGTGGCGGGATATAACCTGAATCTGCGTCATGGCAGCTCTGTACCGGT
GGATCATGGAGTTGCTACCCCGCTCGGTTTTCATGATGTCCATGGTAATGTCTGGC
AGTGGCTTGCGGATGATTTTGCGCCACTTCCCGGTTTTAGAGCACATCCTCTGTAT
CCGGATTTCTCTTTACCTTATTTCGATGACCAACATGCAACACTGCGCGGTGGTAG
CTGGGCAACCACCGGGACCGGTGCATCGCGCTATTACCGTCTTTGGTTTCGCCGG
CACTTTGCTCAGCACGCCGGTTTTAGACTGGCCTAA

6xHis-TgiA-II$_{Myx}$ protein sequence

NCBI Identifier: MCA9526552.1

MGSSHHHHHHSSGLVPRGSHMDVMLPPSLAADRATLLAYFEESYRLCDWLFSSVAE
GALYQQPDPLRHPLIFYVGHPAAFYINKLRLAGLLDAPVDAELEVLFAKGVDPHAPDEL
DRRATWPALDVVRAYRARVFDVVRAVIEATPLPPVVTWDHPLWALHMACEHDRIHFE
TSSVLIRQLPVSQVRAPAGWADGPVGGEPPALAWRSVPRRAVQLGRPPVSDAYGWG
NEYGTLEVDVPPFLATTTLVSNADYARFVADGGYRRPELWTKAGWASLGGATHPRF
WVPEAGGGFSYRAQFDVRPLPRAWPAEVNGHEAAAYARWAGARLPTEQEWAALAA
DAPTHDADVIGVAGYNLNLRHGSSVPVDHGVATPLGFHDVHGNVWQWLADDFAPLP
GFRAHPLYPDFSLPYFDDQHATLRGGSWATTGTGASRYYRLWFRRHFAQHAGFRLA

6xHis-*tgiA-II*$_{San}$ CDS (from Sandaracinaceae bacterium, synthesized, codon optimized)*

ATGGGCAGCAGCCATCATCATCATCATCACAGCAGCGGCCTGGTGCCGCGCGGC
AGCCATATGGAGGACATCATTGCCGATGAGGTTCGTGCGCATGCGCAGCGGGGT
CCTATGCAGAGACGTCGGGAGCCGCTTCCTCCCGATCTTAGACATGTTTGCCGTG
ACTCCGCACGCGCTTATTTTGAGGCGTCATGGAGTCTGAACGAACTTCTGTTTCGT
ACAATTACACACGAACGTGCTTTTTTCACCCAACCGGATCCCCTCCGGCAGCCTCT
TGTCTTCTACTTAGGCCATACGGCGGCGTTCTATGTTAATAAACTGCGCCTTGCTG
GCCTGGCGTCTCACGGGCTTGATGCCCATCTGGATGCATTATTTGCCCGCGGGGT
GGATCCGGAGTCTGCTGACGAATTAGATGGACAGAGTGCCTGGCCATCAGTTGAT
AGTGTTTGGGCGTACCGTCGCGCTGCAAGAGAACAGGTGCTGGCAGCAATCGAA
GCCTTACCGTGGGAGGCCCCAATTACAGAAGAGCGACCAGAATGGGCCGTCCTG
ATGGGTATCGAACACGCTCGTATACACTTCGAAACCTCCTCGATGCTGCTTCGCCA
AGCTCCGGTGGAACATCTCACGCGGCCCCGCGGTTGGCGTTATGCGGATGATGC
GCCAGTGCCAGCCGAAAGATTTGTGCATGTCCCCGCAGGAGATACCGCAGTGGG
CCGTCCGCGCGGCGGTCGCCTCTTTGGATGGGATAACGAATATGGGCACGATACT
CGCGAGGTGCCGGCCTTTGATGTTGGAGCGGACCTCGTCACGAATGCGCAATTTT
TGGCGTTTGTTGATGCTGGGGGTTATCGGGATGAGAGACTCTGGAGCCCAGCTGG
TTGGGCTTGGCGGAGCGCACATGGAGTGGAGGGGCCTCGTTTCTGGTTGAGAGA
GGGTACCCGTCCGCCCCGTTACCGGGCGATGTTTGATGAGTTGCCTCTGCCGTTA
TCATGGCCGGCCGAAGTAAATGCCATTGAAGCGGATGCTTATTGCCGTTGGCGGG
ATGGAGTACGTCTTCCGACCGAAGCTGAATGGTGTCGCATGTTGGATGTTACCGA
GGGAGCGCGCGACGACGAGGAAGGAGCCCATTTCCATCTGCATTTCGAGCTGGG
CTCTCCC*T*GGCCAGTAGGGGCTACCTGTCGTGACCGAGGTCGCGCACTCCATGAT
GTTCGCGGTAATGTATGGCAGTGGTTGTCCGATGATTTCCATCCGCTGCCAGGTTT
TCGAACGCACCCGCTCTATGATGACTTCAGTGTTCCCTATTTTGATGCAGAACATG
CTATGCTCCGCGGCGGAGCCTGGGCCACGACTGGAACCGGGGCATCCGTGCATT
ATAGATTATGGTTCCGTCGGCACTTCTATCAACATGCCGGTTTTAGACTGGTTCGAT
GCGAGTAA

*C1099T mutation with altered base italicized

6xHis-TgiA-II$_{San}$ protein sequence*

NCBI Identifier: MCB9659646.1

MGSSHHHHHHSSGLVPRGSHMEDIIADEVRAHAQRGPMQRRREPLPPDLRHVCRDS
ARAYFEASWSLNELLFRTITHERAFFTQPDPLRQPLVFYLGHTAAFYVNKLRLAGLASH
GLDAHLDALFARGVDPESADELDGQSAWPSVDSVWAYRRAAREQVLAAIEALPWEAP
ITEERPEWAVLMGIEHARIHFETSSMLLRQAPVEHLTRPRGWRYADDAPVPAERFVHV
PAGDTAVGRPRGGRLFGWDNEYGHDTREVPAFDVGADLVTNAQFLAFVDAGGYRDE
RLWSPAGWAWRSAHGVEGPRFWLREGTRPPRYRAMFDELPLPLSWPAEVNAIEADA
YCRWRDGVRLPTEAEWCRMLDVTEGARDDEEGAHFHLHFELGSP*W*PVGATCRDRG
RALHDVRGNVWQWLSDDFHPLPGFRTHPLYDDFSVPYFDAEHAMLRGGAWATTGTG
ASVHYRLWFRRHFYQHAGFRLVRCE

*R367W mutation with altered amino acid italicized.

6xHis-*tgiB*$_{Myx}$ CDS (from Myxococcales bacterium, synthesized, codon optimized)

ATGGGCAGCAGCCATCATCATCATCATCACAGCAGCGGCCTGGTGCCGCGCGGC
AGCCATATGGAGCGAGATGCGCACCAACAAGCCTTACTCGATGCAGCTCTTCGGC
TTGGTGTCGAAGTCGAGTTACTTCCCCTTGCTTGGGGAATAGACGCCGTACGGTAT
GGCGGCGTACTCGTTTTAAAGGGCCGCGTCATACCGTCCCTTTCTCACCAAGCAT
CTCTGTTGATCGATAACAAATTAGCGACTAAGGCCCTTTTAGCTGAAGCTGGTCTG
CCTACGCCAGCAGGCGCTCCATTGACCGGTCTCCTCGACGTGGATCTGCCCGTAT
TACAAGCGCTTTTAGCACAGGGTCCGATCGTAGTAAAGCCCGTGGCTGGCACACA
TGGTCGAGGAGTTCTTCTCGATCCCCCAAGCGCCGAAGCGGCGGCACGTCATGC
GGCCCACCTTGCTGAACCTGCATTGGCGGAAGCATTAGTCGCAGGCGCGGATCTG
CGTCTCCATGCTCTTGGCGGGCGGGTAGTTGCGGCATGTGTTCGAACACCGCCCA
GTGTCACGGGAGACGGTCACACGCCAATTGCAGCGTTAATAGAGGCCTTAGACGC
CGAAGTAAGACGCCCGAACCCGCAAAACCGTGCGGTGTTAGATGCCCACGTAATA
GATGTACTGGCCGAGCAGAGATTGACCCCAACCGACGTGCCCGCAGATGGGCAG
CTCGTACGCTTAGGAGCCCTGGCCAATATGGCTCGTGGAGCGCGAGCAACCGAT
GTGACTGACGCTTTGCACCCACGTTGGCACGCTTGGGTAGAGAGAGCGGGAGCC
GTATTTGGGCTGGACGTTTTCGCCGTCGATGCAATGACTCCTGACCCTTCTGCTGA
TCCTGCAGAAGTTGGCGGGTCAATCCTTGAGTTAAACGCCCGCGCTGAATGGTTA
CACCATACGTTCTCCGCAGGCCGGTGTCACGACATCGGCGCCTTATTACTGCACC
ACCTGGACGTCGTTCCGTGA

6xHis-TgiB$_{Myx}$ protein sequence

NCBI Identifier: MCB9549178.1

MGSSHHHHHHSSGLVPRGSHMERDAHQQALLDAALRLGVEVELLPLAWGIDAVRYG
GVLVLKGRVIPSLSHQASLLIDNKLATKALLAEAGLPTPAGAPLTGLLDVDLPVLQALLA
QGPIVVKPVAGTHGRGVLLDPPSAEAAARHAAHLAEPALAEALVAGADLRLHALGGRV
VAACVRTPPSVTGDGHTPIAALIEALDAEVRRPNPQNRAVLDAHVIDVLAEQRLTPTDV
PADGQLVRLGALANMARGARATDVTDALHPRWHAWVERAGAVFGLDVFAVDAMTPD
PSADPAEVGGSILELNARAEWLHHTFSAGRCHDIGALLLHHLDVVP

6xHis-*tgiB*Fla CDS (from Flavobacteriales bacterium, synthesized, codon optimized)

ATGGGCAGCAGCCATCATCATCATCATCACAGCAGCGGCCTGGTGCCGCGCGGC
AGCCATATGCCAAAGGAAGATAAGGTTGTGGAAGGTACCGCCCAGCACCAATTAA
TCGTAGGAGAAGCCCAGAAACGCGGGTTCGAGGTAGAAGAGAAAGCGTGCTCTG
AGGGTAAGAGATTGACGATTCTCAAGAAGGGCAACCATCAGGAGAAGATCTACTC
AGGCATCCCCGAGAGTTGGGTCAATGATAATACTGCCGAGATATGCGACCAAAAG
CAACTGACTAAGCAACTGTTCCAGGAAATTGAGATTCCTACACCGATTTCTTTTCGT
TTCCAACTGGCGGAAGATCTGGACGAGGAAGTCTTTCGCACCGGCAAGAATTACA
TCTGCAAGCCAGAGGTAGGCACTAACGGCAAAGGCGTGGAGTTCGACATTAAGAA
TCTGGACCTTGTGAAGGGTTACCTGCAACGGAACTCGGCCATCGCAGACACGTTT
CTTTTAGAGGAGTTTGTATCCGGAGAAGACCTTCGCCTGCAGATTATTGGTCGCAA
GATAGTTGCGGCATGTGTGCGGAAGCCCGCATCAGTAATCGGCGACGGTCAATAC
ACCTTGTCCAAATTAATTGAAAAGCGGCAAGCCGAGGTGCAGGCACAAAACCCGG
CCAACCGCTTGGAAATAGATGAACAATCTAAAGAGCTGATCCGGCTGAGTAACTAT
AGCTTCGCAGACGTAATTCCAGAAAATACAGAGATTAGACTTAAGAAAATCGCAAA
CATGGGACAAGGCGCCAAGGCGATCGATGTGACTGACGAAATAGATCCGGCTCTG
CAACATTGGGTTAACCGTATCAGTACTGCCTTGCAAGCACCGTATTTTGCTTTAGAC
ATTATAAGCAACGATCCAAGTAATGAGAAGGAATGTGTAGCTCTGGAATTGAACTT
GCGTGCTGAATGGATGCACCATACCTTCTCGGAGAAGCGAACTCATGACCTGGCG
AAGACGGTTATCGATGAGCTTTTCGGCCCTGAGAAGTGA

6xHis-TgiBFla protein sequence
NCBI Identifier: MCG8576821.1

MGSSHHHHHHSSGLVPRGSHMPKEDKVVEGTAQHQLIVGEAQKRGFEVEEKACSEG
KRLTILKKGNHQEKIYSGIPESWVNDNTAEICDQKQLTKQLFQEIEIPTPISFRFQLAEDL
DEEVFRTGKNYICKPEVGTNGKGVEFDIKNLDLVKGYLQRNSAIADTFLLEEFVSGEDL
RLQIIGRKIVAACVRKPASVIGDGQYTLSKLIEKRQAEVQAQNPANRLEIDEQSKELIRLS
NYSFADVIPENTEIRLKKIANMGQGAKAIDVTDEIDPALQHWVNRISTALQAPYFALDIIS
NDPSNEKECVALELNLRAEWMHHTFSEKRTHDLAKTVIDELFGPEK

6xHis-*tgiB*San CDS (from Sandaracinaceae bacterium, synthesized, codon optimized)

ATGGGCAGCAGCCATCATCATCATCATCACAGCAGCGGCCTGGTGCCGCGCGGC
AGCCATATGAGCAGTGCCCACGGGGAACAAGTTGACCACGGACGGGACCATCCA
ACAAACGAACCGTCAACAACTCAGCATGACCCTCACCAACACGAGTTAATGCGTGC
GGCGGCCAGACTGGGTGTTACCCATTGCGTGGTAGCTGGTGCGCCGGATACGGT
GGCTTATGCGTGGGGTGAGCACGAGGTTCGCGTACGCAAAGGGCGTGTGTTCCC
GAGTCTGACACGCGCTGCGGAAGCCTTATGTGACCATAAACAACGTTGTAAGGAT
GCTCTCGACGCAATCGATGTTCGGACACCTGCTGGTCTCCCATTCTGGAGCGATG
ATACGGATGTCGAGCCACGAATGGCTGCATTGCTGGCCGAGCGCGCCCCATTGGT

TTGTAAGCCAGTGGATGGTACTCATGGTGAGGGTGTTGCGTTAGACCTGACCTCG
GCAGCGGCAGTGCTTACACATGCTCAGCGGCACAGCCCTACTCGCAGAGAGTGG
GTCGCCGAGAGCCAACATGGCGGACACGATCTCCGTCTGCAGGCGATAGCTGGA
CAACTGTTCGCTGCCTGTATGCGAGTCCCTGCGGCTGTTGAGGGCGATGGTGTCC
AGACGGTTGCCCAGTTACTCGCTGCGCGTGATGCAGAAGTGCGCGCGCTCAACC
CTCGGAACCAGCTGACTGTGGACGATGAGGTTCGTGCACTCTTACAAGCCCAGGG
CTTAACCTTAACTTCAGTGCCTGGTGCTGGGCTCCACGTTCCGTTGCGTCAAGTAG
CGAACATGGCACAAGGCGCGCGTGCTATCGATGTCACTGACACCCTTCACCCGGG
CTGGCGGACGAATGTGGCGCGCATTGCCAAACACGTTGGCATTGCTGTGTTCGCA
GTCGACGCACGTGTACGGAGCGCGGAAGATCCGCCCGACGCGGGTGTAGTTTTA
GAGTTGAATGCGCGTCCGGAATGGCTGCACCACACTTTCAGTGAAGGCCGCCAAC
ATGATGTCGGAGCCGCATTATTAAGAGCATGTCTCCCAGGACTGTCTTGA

6xHis-TgiB$_{\mathrm{San}}$ protein sequence

NCBI Identifier: MCB9659647.1

MGSSHHHHHHSSGLVPRGSHMSSAHGEQVDHGRDHPTNEPSTTQHDPHQHELMRA
AARLGVTHCVVAGAPDTVAYAWGEHEVRVRKGRVFPSLTRAAEALCDHKQRCKDAL
DAIDVRTPAGLPFWSDDTDVEPRMAALLAERAPLVCKPVDGTHGEGVALDLTSAAAVL
THAQRHSPTRREWVAESQHGGHDLRLQAIAGQLFAACMRVPAAVEGDGVQTVAQLL
AARDAEVRALNPRNQLTVDDEVRALLQAQGLTLTSVPGAGLHVPLRQVANMAQGARA
IDVTDTLHPGWRTNVARIAKHVGIAVFAVDARVRSAEDPPDAGVVLELNARPEWLHHT
FSEGRQHDVGAALLRACLPGLS

6xHis-*tgiB*$_{\mathrm{Myx2}}$ CDS (from Myxococcota bacterium, synthesized, codon optimized)

ATGGGCAGCAGCCATCATCATCATCATCACAGCAGCGGCCTGGTGCCGCGCGGC
AGCCATATGGACCCACATCAACGGGCATTGGTCAAAGCAGCTCCTCGCCTTGGAG
TCACCGCTACTGTCGAACCCGAGTTAGAAGAGCACCCGTTCGTTCCTGCCCCCGC
AGACGTCGTCACCTACCGCCGCAACGACGCTAGTGCGAGAGTGATACATGGAAGA
ATCTTCGCGCAGTTATCGCCCGAAACTGATGCCCTGTGTGCCGACAAGGCCCGTG
CTAAACGCTTCTTAGAGGCCCTCGGGTTGCCGACTCCTGCCGACCTGCTGGTTCC
TCGTGATACCCCGCACAAATCGTGTGCAGCCATGGTCGAGCCGTTTCTGCACGAT
GCACTTGCTCGCGCCTTCGTAGTGAAGCCCCTTGACGGAACGAACGGGGAAGGT
GTGTACATGGACCTTACCTCTAACGCTCAAGTGATGCGTCGGGTTGTGGGGTTGTT
GGAGCGAGGGGAAGACGTACTGATAGAGGAACAGGCGCCTGGCCAAGACTTACG
TGTGCAAGTGATCGGCAAACAATTAGTCGCGGCCTGCATTCGAGAACCTGCATGT
GTCATTGGCGATGGTACATCAACCATAGCAGAGCTCGCTGTTGAAAAGGACTTGC
GTGTCCAACAAAACAATCCACAGAACCGGTTACTTTTAGACGAGGAGTCTCTTCGA
CTTCTGGCCGGTCAAGATTTGGAACCTACTTCGATTTTAGCACGCGACCAAAAGAT
CTTTGTGAAACGGGTAGCTAACATAGGTCAAGGTGGACTGCCAGTTGACGTTACTG
ATGCCCTGCACCCAGAATACCACGCATGGTGCGCTACTATTGCAGAGGCTTTGGG

ATTAGATATATTCGCGCTTGATTTGATAGCAGACAACGCGCACGACTCTCCTATGA
AGACAAACGCGGTGATCTTGGAGATCAACGCTCAGCCCCAATGGCTTCACCACAC
CTTTTCAGAGGTGCGCACTCACGACATCCCCGCCCTGTTATTCGATGCGCTCCTG
GGCGCGCCGGCCTGA

6xHis-TgiB$_{Myx2}$ protein sequence
NCBI Identifier: MEC9439981.1
MGSSHHHHHHSSGLVPRGSHMDPHQRALVKAAPRLGVTATVEPELEEHPFVPAPAD
VVTYRRNDASARVIHGRIFAQLSPETDALCADKARAKRFLEALGLPTPADLLVPRDTPH
KSCAAMVEPFLHDALARAFVVKPLDGTNGEGVYMDLTSNAQVMRRVVGLLERGEDVL
IEEQAPGQDLRVQVIGKQLVAACIREPACVIGDGTSTIAELAVEKDLRVQQNNPQNRLL
LDEESLRLLAGQDLEPTSILARDQKIFVKRVANIGQGGLPVDVTDALHPEYHAWCATIA
EALGLDIFALDLIADNAHDSPMKTNAVILEINAQPQWLHHTFSEVRTHDIPALLFDALLGA
PA

6xHis-*tgiB-II*$_{Sbu}$ CDS (from *Streptomyces buecherae*, synthesized, codon optimized)
ATGGGCAGCAGCCATCATCATCATCATCACAGCAGCGGCCTGGTGCCGCGCGGC
AGCCATATGGCGCAACCCTTGACAGTGGGAGTTGAAGAGGAGTTCTTTCTGGTCG
ACCCAGACAGTGGTGCTGCCGTCCCAGCCGCCGAGCAAGTGCTTGCAGCGGCTG
AACCTACGACGCGTGTGAAGCTGGAGTTGTCAGCAGCCCTTGTCGAAACGGTGAC
GGAACCACACCGCGAATTGAGACTTCTGGCAGCCGATCTTAGAGGTGCTCGTGCA
GAAGCCGCCGCGGCGGCAGAAGCGGTTGGCTGCGCTGTTTTGCCGTCCGCCACC
GAACTGTTAGCTGGGCCGTGCCATCCGCCAACTGGCGGTGCGCGTAATCGCAGA
ATACATGCGCGCTACGGAGCCTTTGTGGATGGTCGCATGACTTGTGGGTTGCACG
TGCATGTGGGGATGCCAGACCGTGAACGGGCGGTTAGAGTCGGTAACCACTTACG
TCCGTGGCTTCCAGTGTTACATGCTTTGTCTGCGAACTCCCCGTTCTCACAGGGAC
AAGATACTGGTTTCGCTTCATTCCGGAGTATGTGGTGGGCCTACAAGCCCGTCGC
AGGTCCCGCCCCTCTGTTTCGAGACGCAGCGGAATACGATGCTTTGCTTGATCGG
CTGATAGCGTCTGGAGTCATCCTGGATCGCAAGATGGCTTTCTGGTTTGCGCGAC
CGTCAGAGCACGTGCCTACTATTGAGGTTCGAGTCATGGACGTTTGCCCCACAAG
TGACACCGCTGTACTGCTTGCTGGACTGACCCGTGGACTGGTGGCTACCTGTCTC
GCAGAGGTAGAGCGCGACGGTCCCCCGCCAGCAATCTCAGATGTCGTCCTGGCG
GCTGCGCATTGGCAAGCTGCAGCCCACGGTCTTGAAGGTGATGCGGTAGACGTG
GTGACAGCGCTCCCGGTACCGGTTTGGACCCTGGTTGAGCGTCTGGTGAAGCATA
TAGCCCCAGCCTTGAGCGAAGCAGGTGATCTCGATTGGGTCGAGGATGCTCTTTC
TCGGACTAGAGGGAGAGGCTCCTGGGCTGCGCACCAACGTGGCATCGTGCGCGC
GGGCGGTGGCTTGCCTGATGTGGCGGCGCGCTTACGTGGTTTGTTGTTGACTTAG

6xHis-TgiB-II$_{Sbu}$ protein sequence
NCBI Identifier: WP_176162567.1

MGSSHHHHHHSSGLVPRGSHMAQPLTVGVEEEFFLVDPDSGAAVPAAEQVLAAAEP
TTRVKLELSAALVETVTEPHRELRLLAADLRGARAEAAAAAEAVGCAVLPSATELLAGP
CHPPTGGARNRRIHARYGAFVDGRMTCGLHVHVGMPDRERAVRVGNHLRPWLPVLH
ALSANSPFSQGQDTGFASFRSMWWAYKPVAGPAPLFRDAAEYDALLDRLIASGVILDR
KMAFWFARPSEHVPTIEVRVMDVCPTSDTAVLLAGLTRGLVATCLAEVERDGPPPAIS
DVVLAAAHWQAAAHGLEGDAVDVVTALPVPVWTLVERLVKHIAPALSEAGDLDWVED
ALSRTRGRGSWAAHQRGIVRAGGGLPDVAARLRGLLLT

Table S1. Plasmids used or generated in this study.

Plasmid	Purpose	Source
pET15b(+)	Amp^R, Expression vector	Novagen
pET15b(+)_6xHis-$AshB_{Ake}$	Expression of 6xHis-$AshB_{Ake}$	This study
pET28b(+)	Kan^R; Expression vector	Novagen
pET28b(+)_6xHis-$AshA_{Ake}$	Expression of 6xHis-$AshA_{Ake}$	This study
pET28b(+)_6xHis-$CsdA_{Nos}$	Expression of 6xHis-$CsdA_{Nos}$	This study
pET28b(+)_6xHis-$EgtB_{Csu}$	Expression of 6xHis-$EgtB_{Csu}$	Ref[10]
pET28b(+)_6xHis-$OvoA_{Th2}$	Expression of 6xHis-$OvoA_{Th2}$	Ref[11]
pET28b(+)_6xHis-$OvoB_{Eta}$	Expression of 6xHis-$OvoB_{Eta}$	Ref[10]
pET28b(+)_6xHis-$TgiA_{Sva}$	Expression of 6xHis-$TgiA_{Sva}$	This study
pET28b(+)_6xHis-$TgiB_{Sva}$	Expression of 6xHis-$TgiB_{Sva}$	This study
pET28b(+)_6xHis-$TgiAII_{Myx}$	Expression of 6xHis-$TgiBII_{Myx}$	This study
pET28b(+)_6xHis-$TgiB_{Myx}$	Expression of 6xHis-$TgiB_{Myx}$	This study
pET28b(+)_6xHis-$TgiB_{Fla}$	Expression of 6xHis-$TgiB_{Fla}$	This study
pET28b(+)_6xHis-$TgiB_{San}$	Expression of 6xHis-$TgiB_{San}$	This study
pET28b(+)_6xHis-$TgiB_{Myx2}$	Expression of 6xHis-$TgiB_{Myx2}$	This study
pET28b(+)_6xHis-$TgiBII_{Sbu}$	Expression of 6xHis-$TgiBII_{Sbu}$	This study
pGro7	Cm^R; GroEL/GroES Chaperone	Takara Bio
pIB139	Apr^R, Integration into *Streptomyces* genomes	Deng Lab
pIB139_ashABC	Heterologous expression of *ash* cluster	This study

Table S2. Bacterial strains used or generated in this study.

Strain	Purpose	Source
Amycolatopsis decaplanina NRRL B-24209	Contains *ash* cluster	NRRL
Amycolatopsis keratiniphila subsp. *keratiniphila* NRRL B-24117	Contains *ash* cluster	NRRL
Amycolatopsis keratiniphila subsp. *nogabecina* NRRL B-24206	Contains *ash* cluster	NRRL
Amycolatopsis roodepoortensis NRRL B-24959	Control for *ash* cluster	NRRL
Streptomyces varsoviensis DSM 40346	Contains *tgi* cluster	DSMZ
Streptomyces albus J1074 *attB::pIB139-ashABC*	Heterologous expression of actinothioneine	This study
Streptomyces albus J1074 *attB::pIB139*	Control for heterologous expression	Deng Lab
E. coli DH5α	Host strain for cloning	NEB
E. coli BL21(DE3)	Host strain for protein expression	NEB
E. coli BL21(DE3) + pGro7 + pET28b(+)_6xHis-AshA$_{Ake}$ + pET15b(+)_6xHis-AshB$_{Ake}$	Heterologous expression of actinothioneine	This study
E. coli BL21(DE3) + pGro7 + pET28b(+) + pET15b(+)	Control for *E. coli* heterologous expression	This study
E. coli ET12567	Host strain for *Streptomyces* genetics	Deng Lab

Table S3. NMR assignments for actinothioneine. NMR spectra were recorded in D_2O. The numbering scheme is shown in the diagram above the table. Full spectra are shown in **Figure S8.**

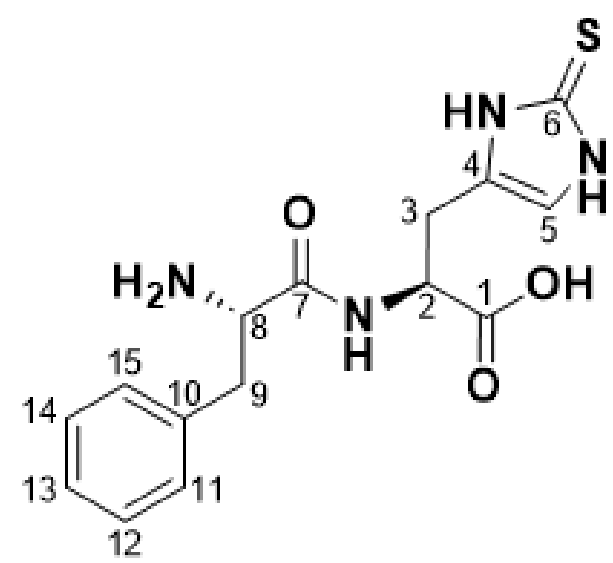

Pos.	δH (m, J (Hz))	δC
1	–	175.9
2	4.25 (dd, 8.3, 4.9)	54.5
3	2.87 (dd, 15.5, 8.3)<br>2.99 (dd, 15.5, 4.9)	27.2
4	–	127.0
5	6.60 (s)	113.9
6	–	154.7
7	–	169.4
8	4.14 (dd, 7.0, 7.0)	54.6
9	3.10 (dd, 14.2, 7.4)<br>3.15 (dd, 14.2, 6.7)	37.2
10	–	134.1
11	7.21 (m)	129.3
12	7.34 (m)	129.1
13	7.31 (m)	127.8
14	7.34 (m)	129.1
15	7.21 (m)	129.3

Table S4. Primers used in this study

Primer	Sequence (5’ -> 3’)	Purpose
Ash-F	GGGGATCTTGATCTGCTCGA	Amplifying *ash* cluster from *A. decaplanina*
Ash-R	CGAACTGCTGACTCGCTACA	

Table S5. Calculated vs. Observed *m/z* values in Heterologous Expression.

Species and Cluster	Derivatized Product	Expected	Observed	Δppm
E. coli + *ash* cluster	2-thiohistidine sulfoxide	291.0758	291.0753	1.7
E. coli + *ash* cluster	mBBr–2-thiohistidine	378.1231	378.1231	<0.1
E. coli + *ash* cluster	mBBr–ASH	525.1915	525.1915	<0.1

Table S6. Crystallographic data processing and refinement statistics for the AshA structure.

**PDB ID**	**9PHY**
**Data Collection**[a]	
Space group	$P\ 2_1\ 2_1\ 2_1$
Unit cell (Å)	a = 50.573, b = 65.071, c = 156.578<br><br>α = β = γ = 90
Wavelength (Å)	0.9202
Resolution range (Å)	42.48 – 1.112 (1.14 – 1.11)
Total observations	2277172 (48242)
Total unique observations	199485 (11600)
$CC_{1/2}$	1 (0.706)
I/σ_1	20.60 (2.16)
Completeness (%)	98.16 (80.80)
R_{merge}	0.05482 (0.5788)
R_{pim}	0.01616 (0.3151)
Redundancy	11.4 (4.2)
**Refinement Statistics**[a]	
Resolution range (Å)	42.48 – 1.11
Reflections (total)	199480 (11605)
Reflections (test)	2000 (116)
Total atoms refined	4649
Solvent	591
R_{work}/R_{free}	0.1752/0.1797
RMSDs	
Bond lengths (Å)/angles (°)	0.006/0.92
Ramachandran plot	
Favored/allowed (%)	98.8/1.2
Mean B values (Å^2)	
Protein	12.56
Ligands	12.01
Solvent	22.91

[a] Values in parentheses refer to the high-resolution shell.

Table S7. Calculated vs. Observed *m/z* values in HR-MS experiments.

Enzyme[s]	[Derivatized] Product	Expected	Observed	Δppm
$AshA_{Ake}$ + CsdA (lyase)	mBBr–2-thioHis	378.1231	378.1231	<0.1
$AshB_{Ake}$	mBBr–actinothioneine	525.1915	525.1915	<0.1
$AshB_{Ake}$	Trp–(mBBr–2-thioHis)	564.2024	564.2025	0.2
$AshB_{Ake}$	Met–(mBBr–2-thioHis)	509.1635	509.1622	2.6
$AshB_{Ake}$	Leu–(mBBr–2-thioHis)	491.2071	491.2058	2.6
$AshB_{Ake}$	Tyr–(mBBr–2-thioHis)	541.1864	541.1847	3.1
$TgiA_{Sva}$ + CsdA (lyase)	mBBr–2-thioHis	378.1231	378.1231	<0.1
$TgiB_{Sva}$	mBBr–thioglutine	507.1656	507.1658	0.4
TgiA-II_{Myx} + CsdA (lyase)	mBBr–2-thioHis	378.1231	378.1231	<0.1
TgiA-II_{San} + CsdA (lyase)	mBBr–2-thioHis	378.1231	378.1231	<0.1
$TgiB_{Fla}$	mBBr–thioglutine	507.1656	507.1629	5.3
$TgiB_{San}$	mBBr–thioglutine	507.1656	507.1656	<0.1
$TgiB_{San}$	Gly–(mBBr–2-thioHis)	435.1445	435.1445	<0.1
$TgiB_{Myx}$	mBBr–thioglutine	507.1656	507.1649	1.4
$TgiB_{Myx}$	Gly–(mBBr–2-thioHis)	435.1445	435.1422	5.3
$TgiB_{Myx2}$	mBBr–thioglutine	507.1656	507.1647	1.8
$TgiB_{Myx2}$	Gly–(mBBr–2-thioHis)	435.1445	435.1442	0.7
TgiB-II_{Sbu}	mBBr–thioglutine	507.1656	507.1636	3.9

Table S8. NMR assignments for thioglutine derivatized with mBBr. NMR spectra were recorded in D_2O. The numbering scheme is shown in the diagram above the table. Full spectra are shown in **Figure S11.**

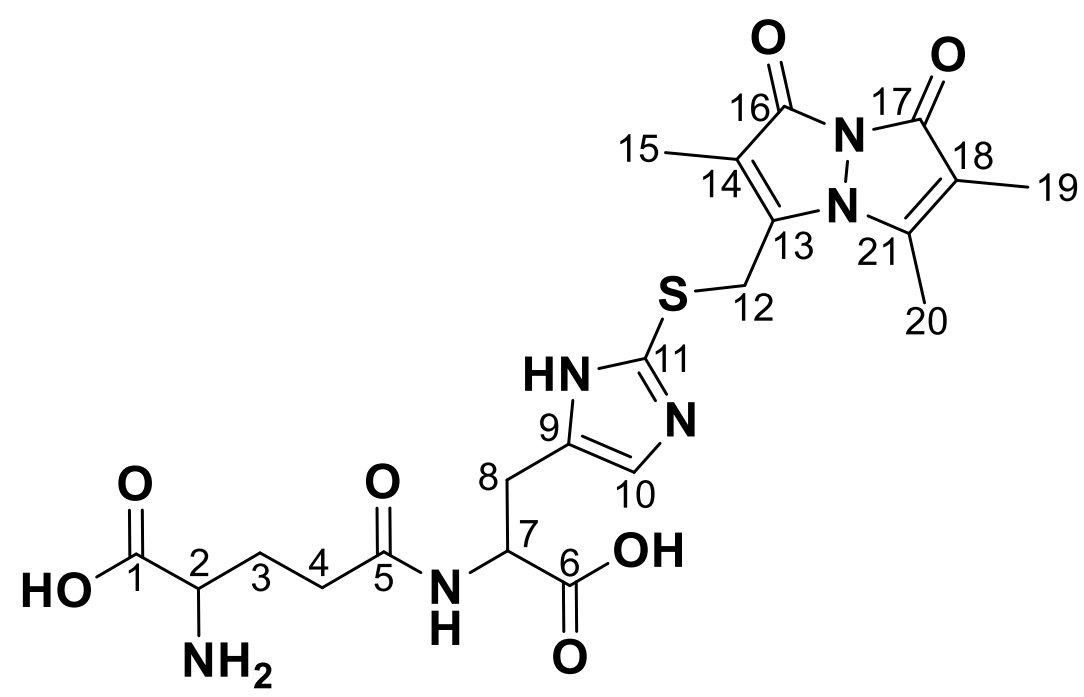

Pos.	δH (m, J (Hz))	δC
1	–	173.9
2	3.63 (t, 6.5)	54.3
3	1.97 (m)	26.2
4	2.32 (m)	31.7
5	–	174.0
6	–	176.9
7	4.29 (dd, 9.2, 4.2)	54.7
8	3.05 (dd, 15.2, 4.2)<br>2.86 (dd, 15.2, 9.2)	28.4
9	–	135.7
10	7.09 (s)	121.4
11	–	133.9
12	4.06 (s)	28.8
13	–	146.6
14	–	113.9
15	1.20	4.9
16	–	162.2
17	–	163.1
18	–	111.4
19	1.70 (s)	5.6
20	2.32 (s)	11.1
21	–	149.7

Figure S1. Biosynthesis of thio/selenoimidazoles. (A) Biosynthesis of ergothioneine. (B) Biosynthesis of the dipeptides actinothioneine and thioglutine. (C) Biosynthesis of the selenoimidazoles selenoneine and ovoselenol. (D) Biosynthesis of ovothiol and *N*α-acetyl-5-thiohistidine.

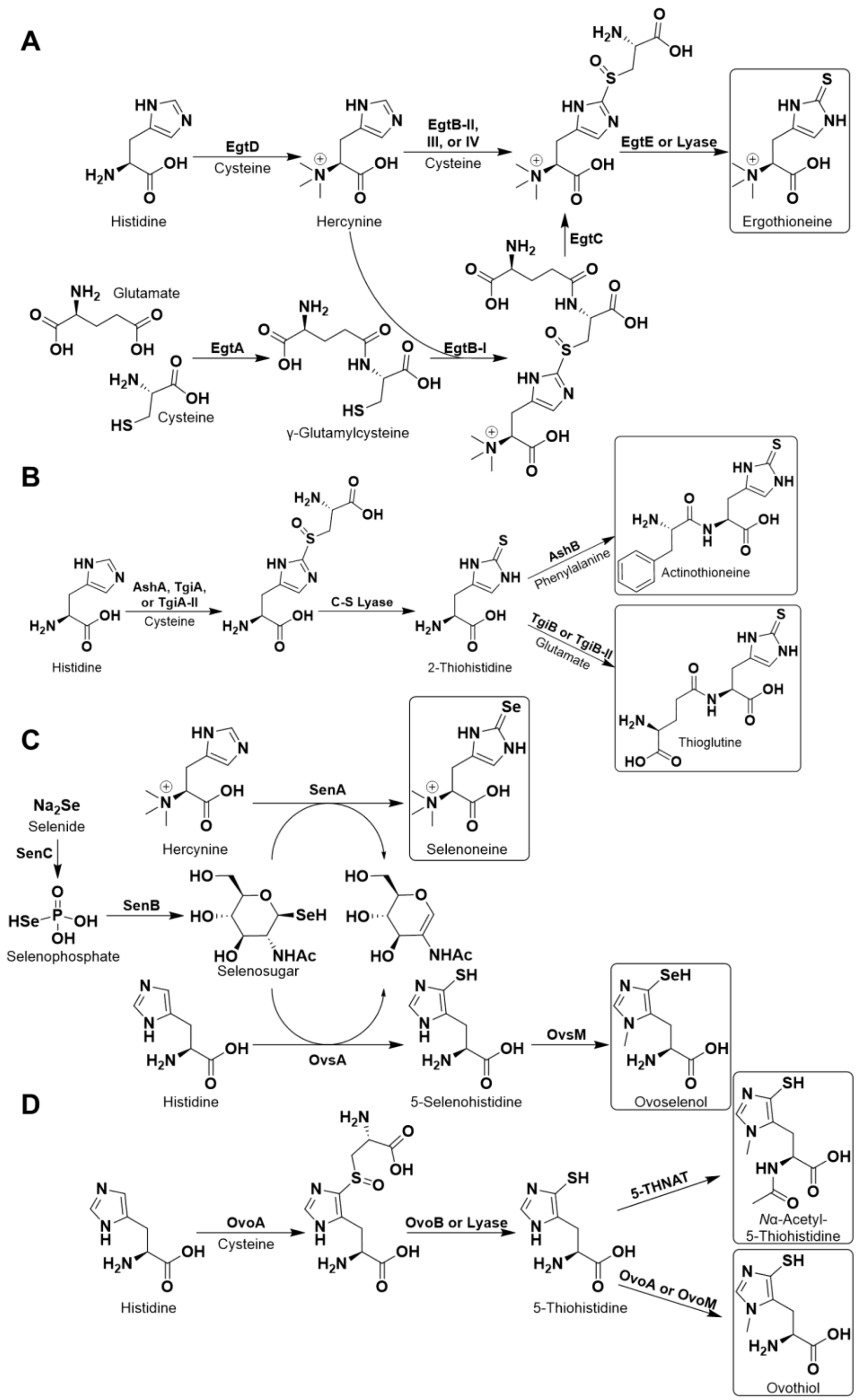

Figure S2. *Ash* clusters in *Amycolatopsis* spp. reveal cluster boundaries. Shown are genome regions selected from a BLAST search of $AshA_{Ake}$ and its surrounding genes. *A. lurida* and *A. roodepoortensis*, two highly-related strains without the cluster, indicate only three genes are involved in actinothioneine synthesis and export. The presence of the methyltransferase without the other three biosynthetic modalities in *Amycolatopsis* sp. BJA-103 implies the methyltransferase is not involved in this pathway.

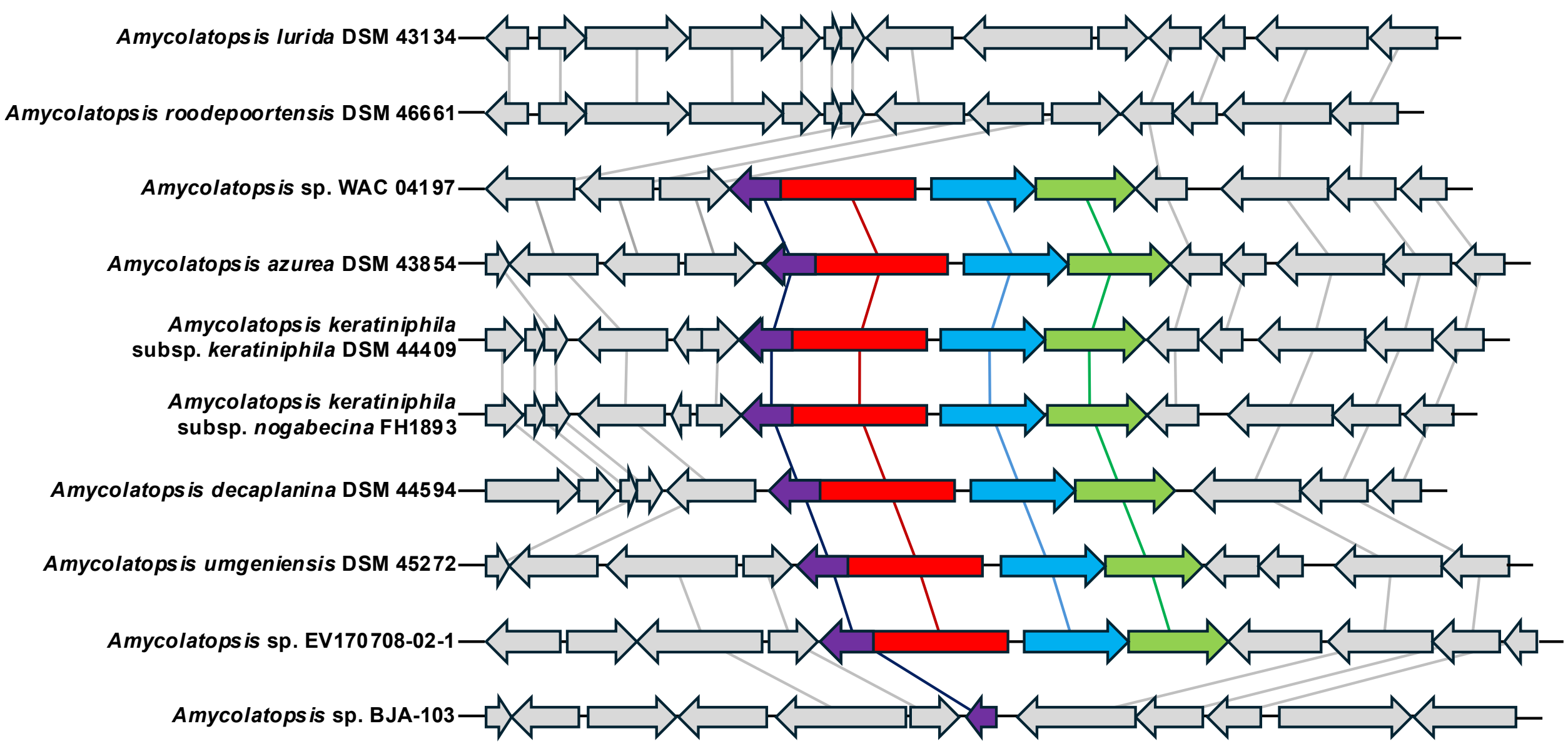

Figure S3. NMR spectral analysis of NHISS-mediated synthesis of histidine-based sulfoxides. Regioselectivity of NHISS enzymes when presented with histidine and cysteine as determined by the downfield ^{1}H-NMR shifts. The peaks at 7.0 and 7.7 ppm correspond to the ^{1}H-shifts of the unmodified imidazole sidechain of histidine (highlighted in green). The peaks at 7.9 ppm and 7.24 ppm correspond to the sole sidechain-^{1}H of histidine-5-sulfoxide (highlighted in pink) and histidine-2-sulfoxide (highlighted in teal), respectively. $EgtB_{Csu}$[10] and $OvoA_{Th2}$[11] are controls with known regioselectivity. All three new NHISS enzymes studied herein ($AshA_{Ake}$, $TgiA_{Sva}$ and $TgiA\text{-}II_{Myx}$), while derived from OvoA, generate the sulfoxide with a distinct regioselectivity at the 2-carbon of the sidechain imidazole.

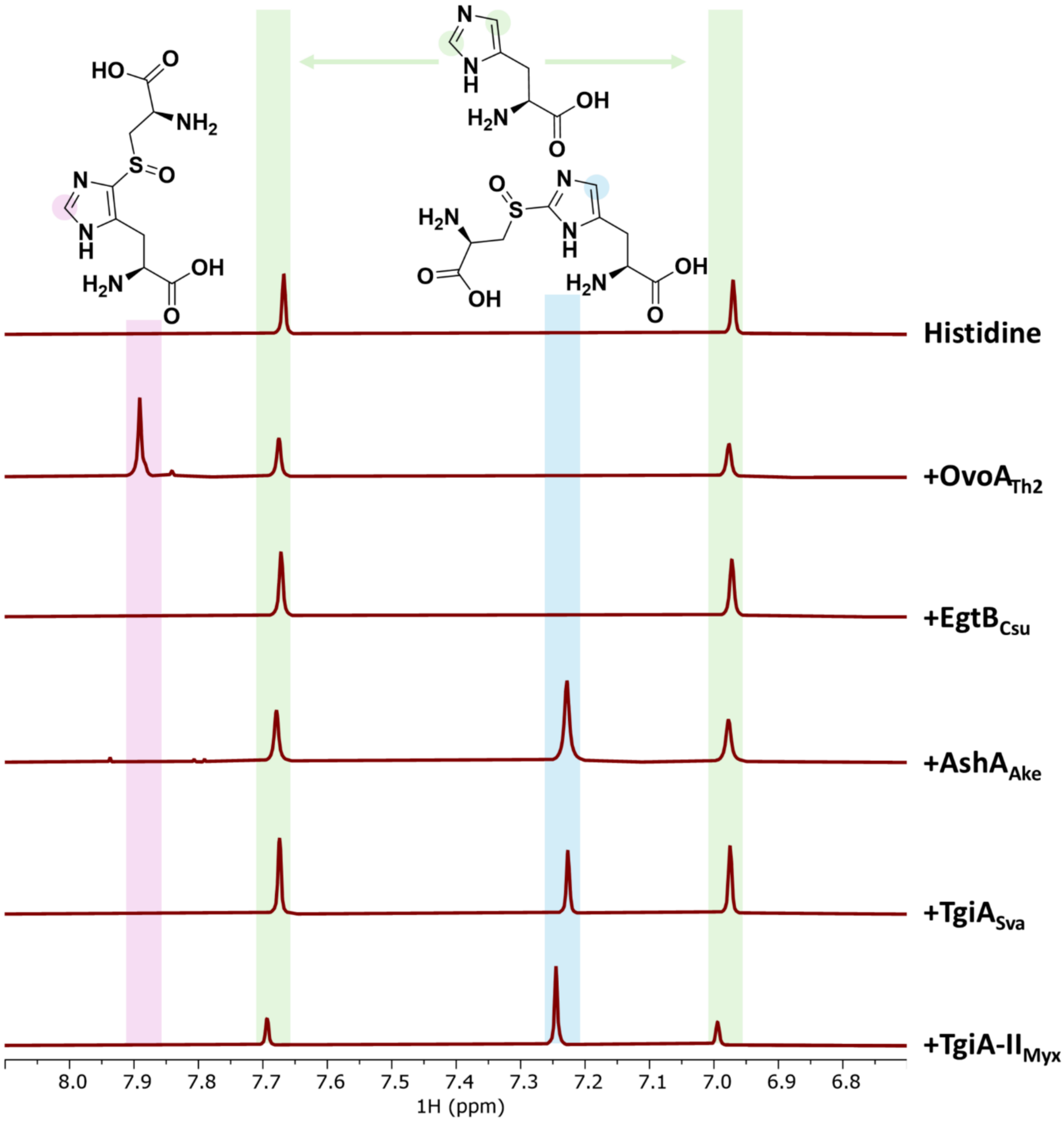

Figure S4. NMR spectral analysis of NHISS sulfoxides. Regioselectivity of NHISS enzymes when presented with hercynine and cysteine, as determined by the downfield ^{1}H-NMR shifts. The peaks at ~6.95 and ~7.65 ppm correspond to the proton shifts of the unmodified imidazole sidechain of hercynine (highlighted in green). The peak at 7.2 ppm corresponds with the sidechain-^{1}H of hercynine-2-sulfoxide (highlighted in teal), while the sidechain-^{1}H of a hercynine-5-sulfoxide would be expected around 7.9, highlighted in pink. $EgtB_{Csu}$[10] and $OvoA_{Th2}$[11] are controls with known regioselectivity. Of the three new NHISS enzymes studied herein, ($AshA_{Ake}$, $TgiA_{Sva}$, and $TgiA\text{-}II_{Myx}$) only $TgiA\text{-}II_{Myx}$ can accept hercynine as substrate to generate the corresponding 2-sulfoxide product, while the other enzymes have strict preference for histidine.

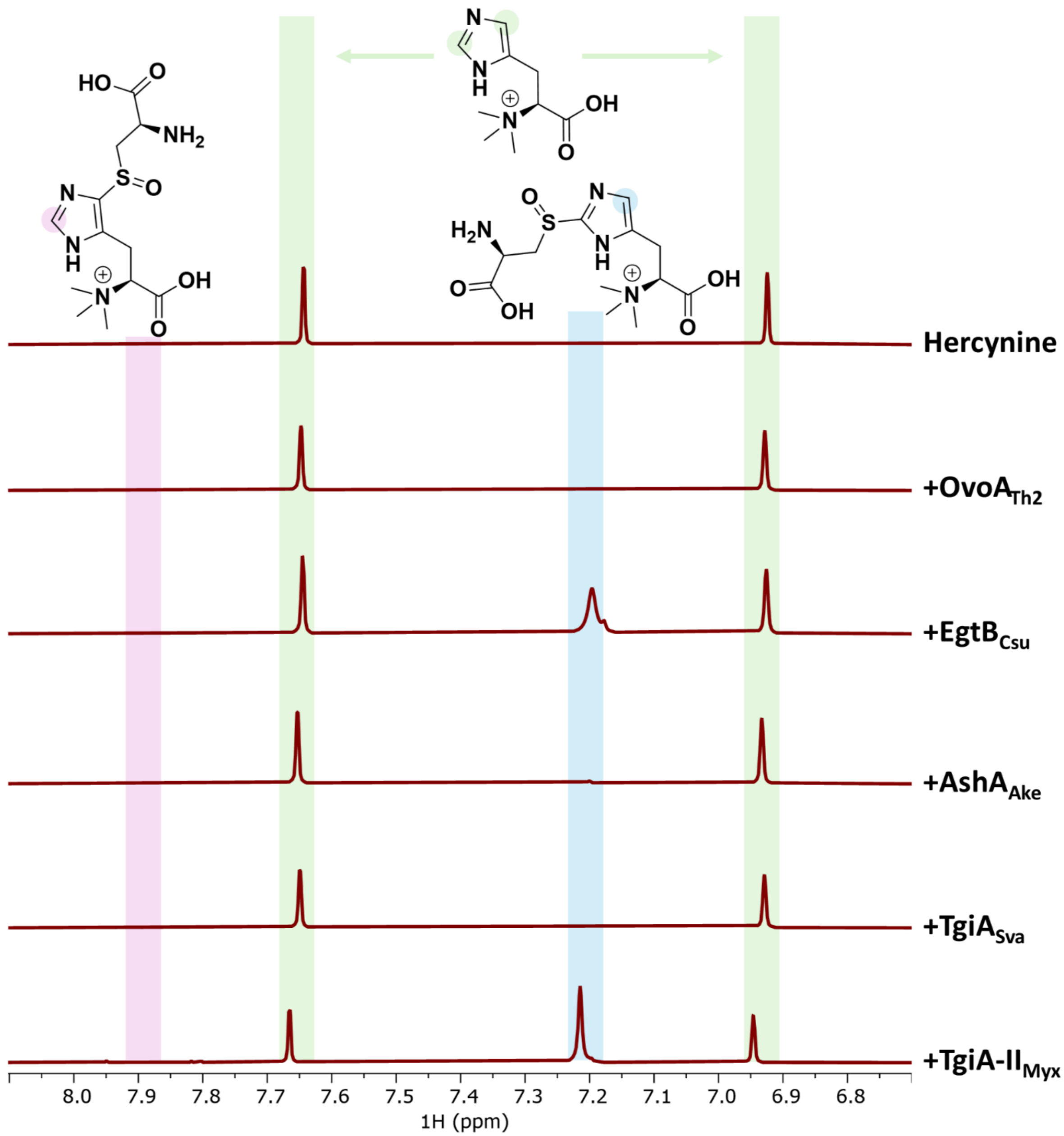

Figure S5. AshB selectivity for leucine over isoleucine. AshB generates a peptide bond between leucine and 2-thiohistidine, as seen by HR-MS. Shown are extracted ion chromatograms for 2-thiohistidine (orange) and the dipeptide product (blue). The control trace contains 2-thiohistine and AshB only in the absence of other amino acids. Traces labeled Isoleucine and Leucine contain the full reaction with the canonical amino acid indicated. Significant dipeptide product is observed with Leu and minimal, if any, dipeptide is seen with Ile, indicating AshB's strong preference for Leu. Extracted $(M+H)^+$ correspond to mBBr-derivatized 2-thiohistidine (378.1231) and the mBBr-derivatized [iso]leucyl–2-thiohistidine dipeptide (491.2071).

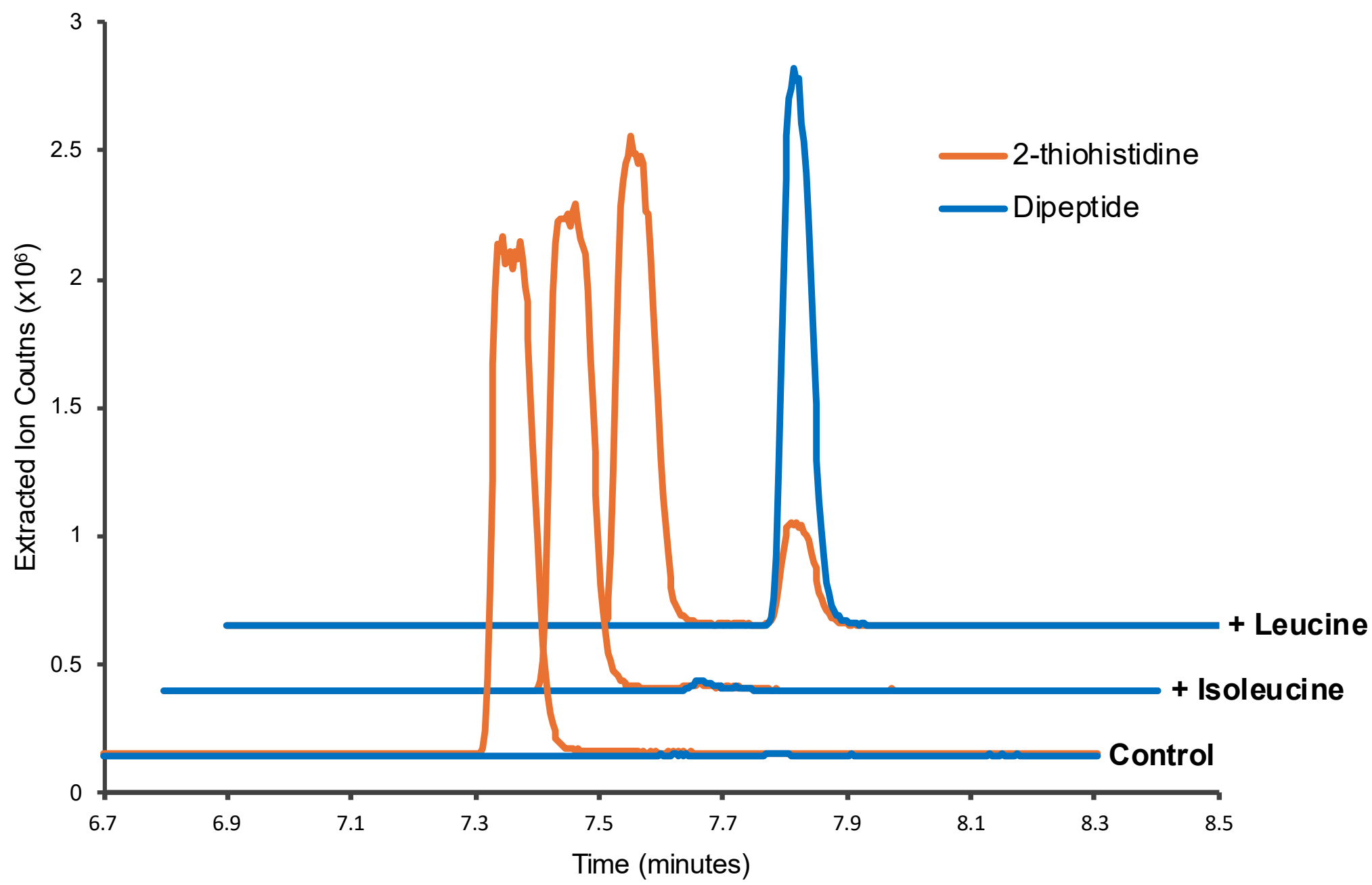

Figure S6. In-source fragmentation shows connectivity of actinothioneine. Shown are extracted ion traces for mBBr-derivatized dipeptide products in the absence (light orange) and presence (dark orange) of AshB as well as the *y*-ion of mBBr–2-thiohistidine due to in-source fragmentation in the absence (pink) and presence (purple) of AshB. In the absence of AshB, no dipeptide is observed as expected (light orange) and only the 2-thiohistidine substrate derivatized with mBBr can be extracted (pink). In presence of AshB, five mBBr–dipeptide products are observed: Met–2-thiohistidine and Tyr–2-thiohistidine (7.55 min), Leu–2-thiohistidine (7.65 min), Phe–2-thiohistidine (7.8 min), and Trp–2-thiohistidine (8.1 min). In each case, the *y*-ion for mBBr–2-thiohistidine is seen at the same retention time, indicating it is the *C*-terminal residue of the dipeptide product.

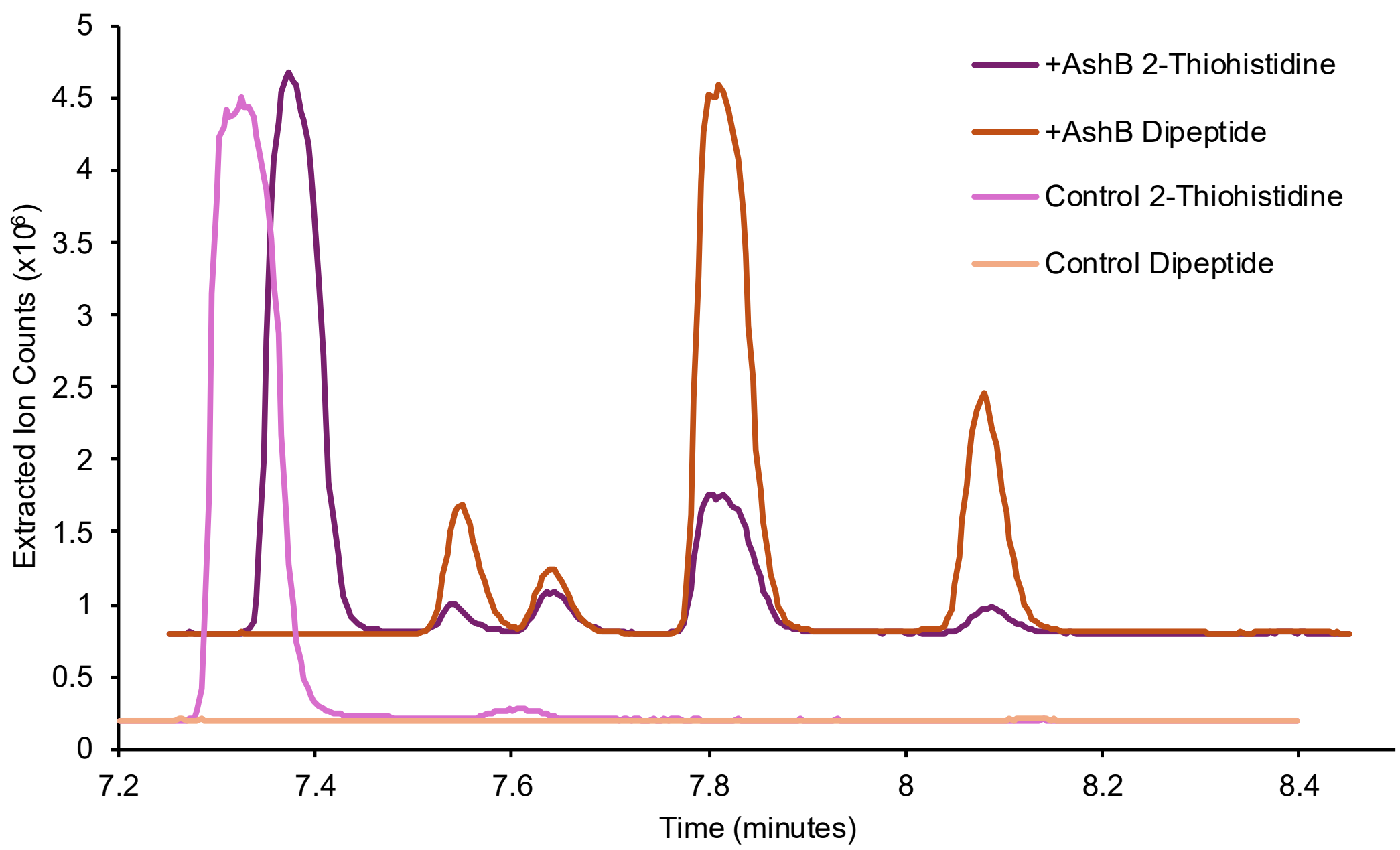

Figure S7. NHISS-associated ATP-grasps do not create dipeptides from 5-thiohistidine. (A) Reaction of AshB$_{Ake}$ with all 20 canonical amino acids and 5-thiohistidine; extracted ion chromatograms are shown for select dipeptides. Only a minimal amount (<0.5% relative to reaction with 2-thiohistidine) of the Phe–5-thiohistidine dipeptide product is observed, with any other dipeptide products, if formed at all, being below the limit of detection. (B) Analogous experiment as in panel A with TgiB$_{Sva}$ incubated with 5-thiohistidine. No dipeptide products are observed in this case. (C) Analogous experiment as in panel A with additional TgiB enzymes studied herein. Each enzyme was incubated with 5-thiohistidine. No dipeptide products are observed. The small peaks correspond to the 2-thiohistidine product, presumably as a contaminant in the 5-thiohistidine sample. The reaction with TgiB$_{Fla}$ and 2-thiohistidine from **Figure 6** (labeled "2 +TgiB$_{Fla}$") is included for comparison. (D) Analogous experiment as in panel A with TgiB-II$_{Sbu}$ incubated with 5-thiohistidine. The small peaks correspond with the 2-thiohistidine product due to trace 2-thiohistidine contamination. TgiB$_{San}$ is included as a control with known reactivity.

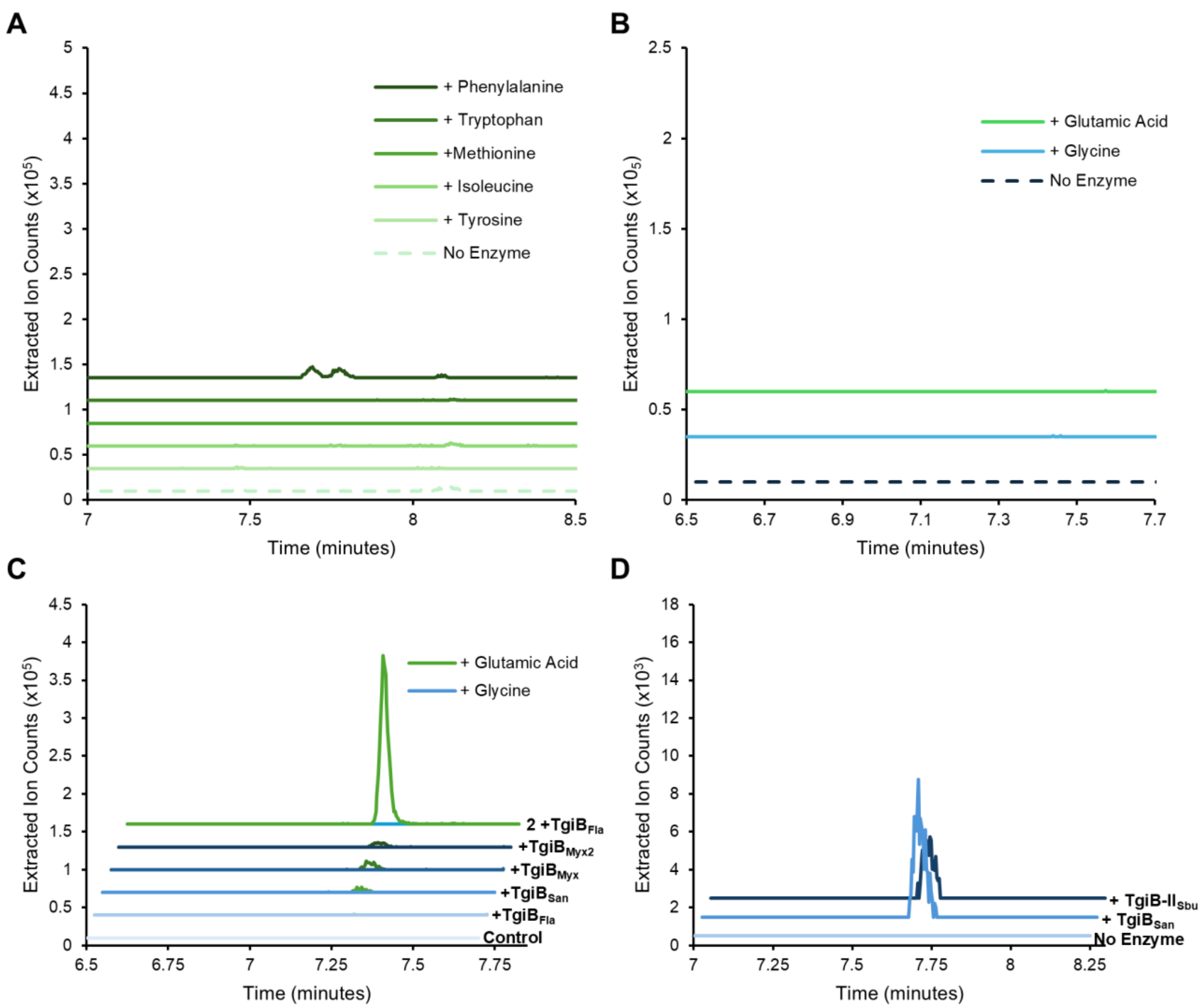

Figure S8. NMR spectra of Actinothioneine. Spectra were recorded in D_2O at 500 MHz. Shown in order are (A) ^1H, (B) ^1H-^1H COSY, (C) ^1H-^{13}C HSQC, and (D) ^1H-^{13}C HMBC spectra.

(A) ^1H spectrum of actinothioneine in D_2O at 500 MHz.

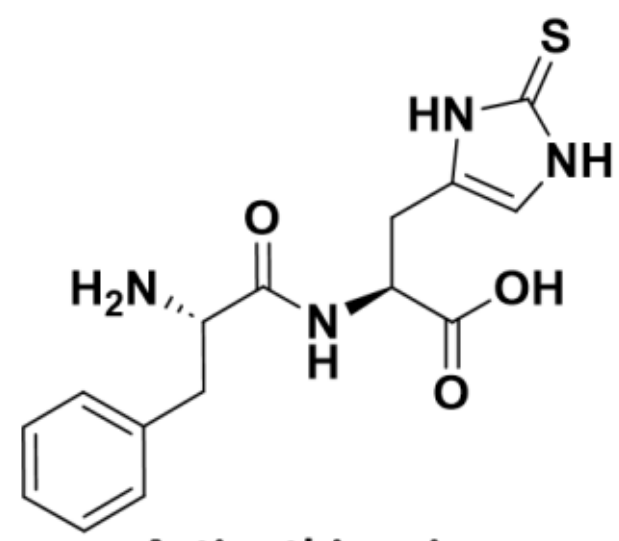

Actinothioneine

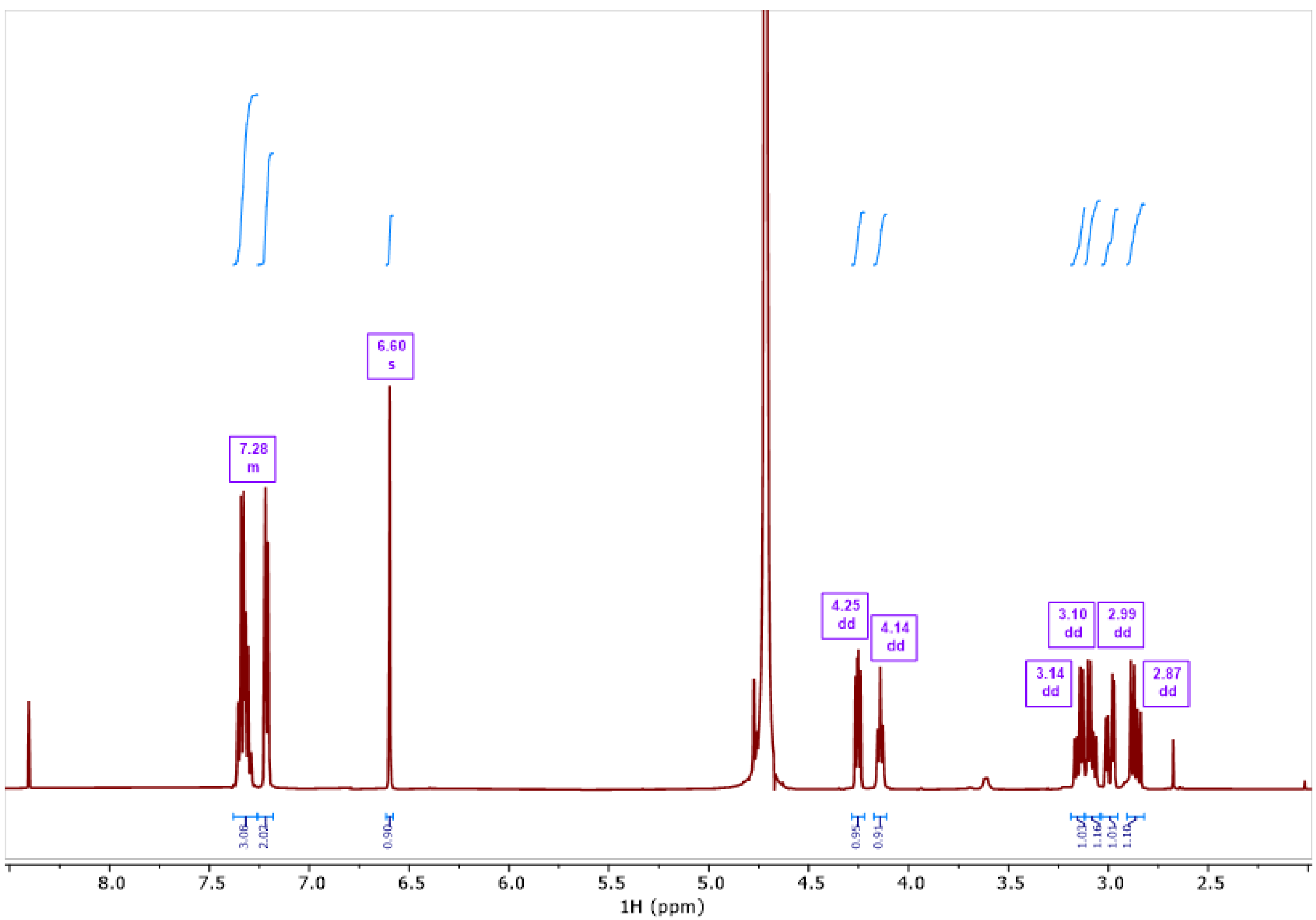

(B) ^{1}H-^{1}H COSY spectrum of actinothioneine in D_2O at 500 MHz.

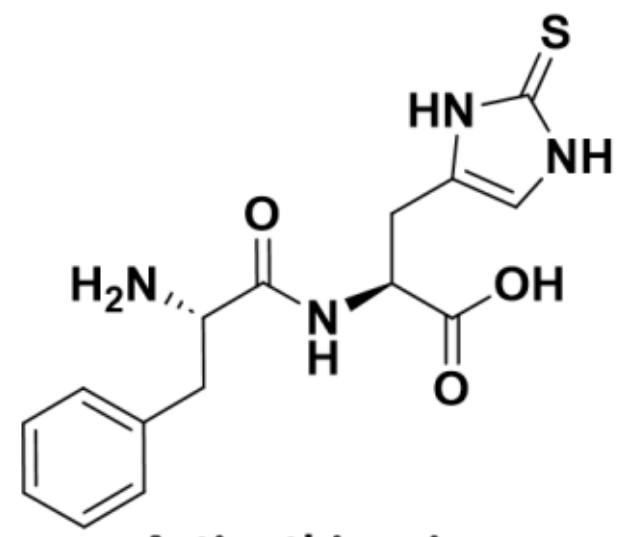

Actinothioneine

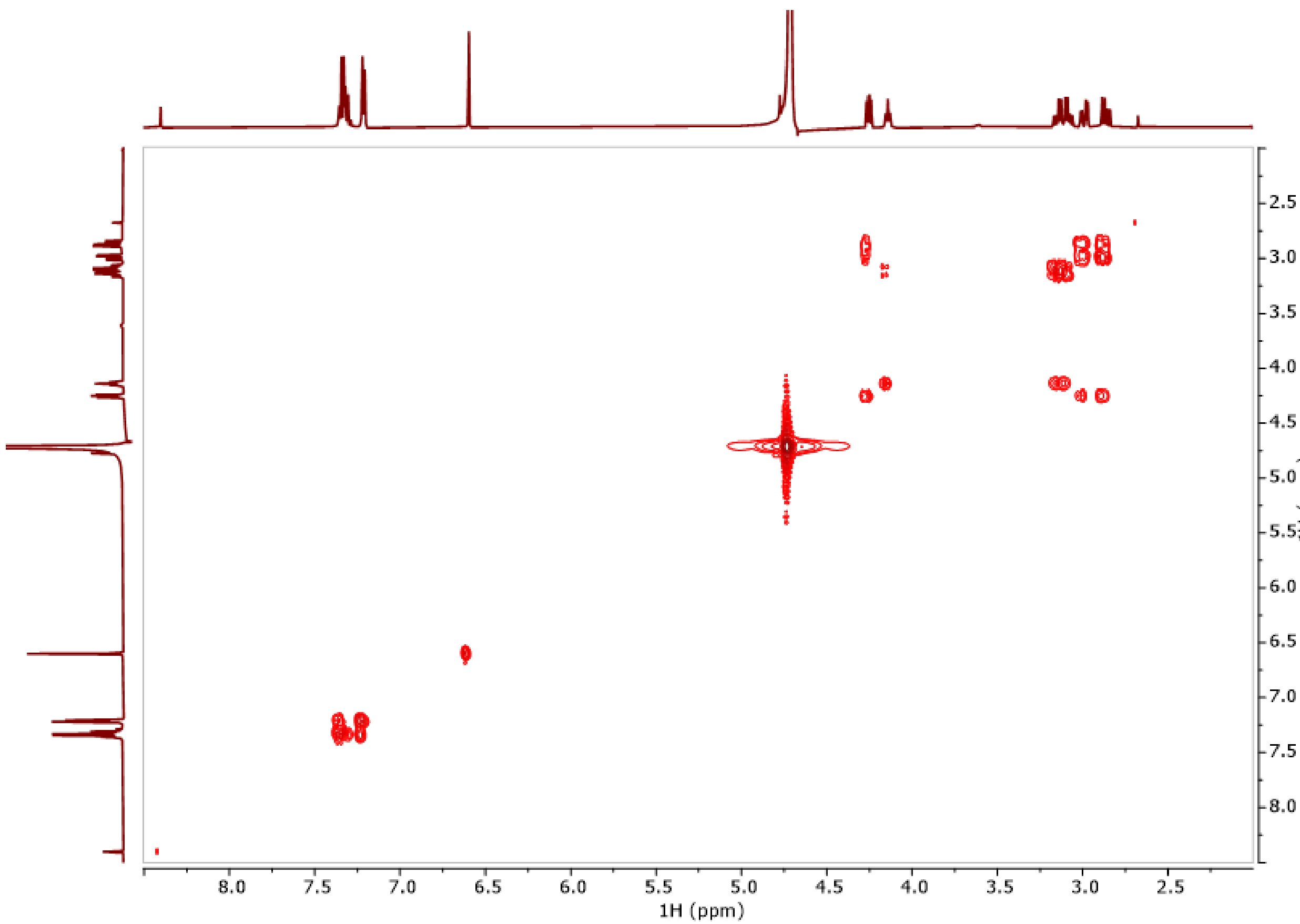

(C) ^{1}H-^{13}C HSQC spectrum of actinothioneine in D_2O at 500 MHz.

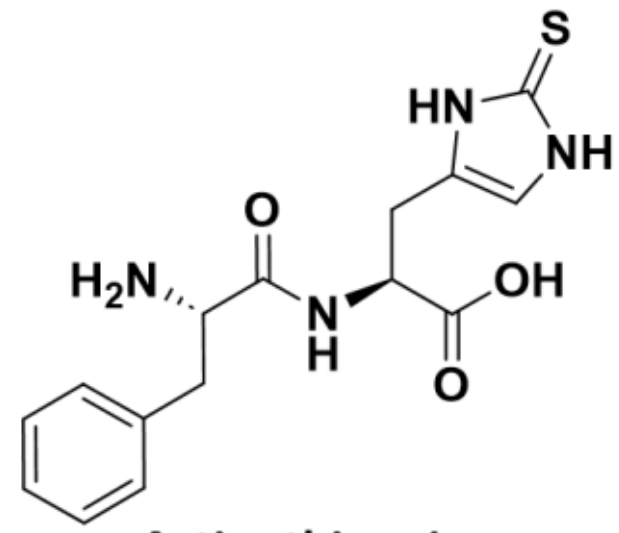

Actinothioneine

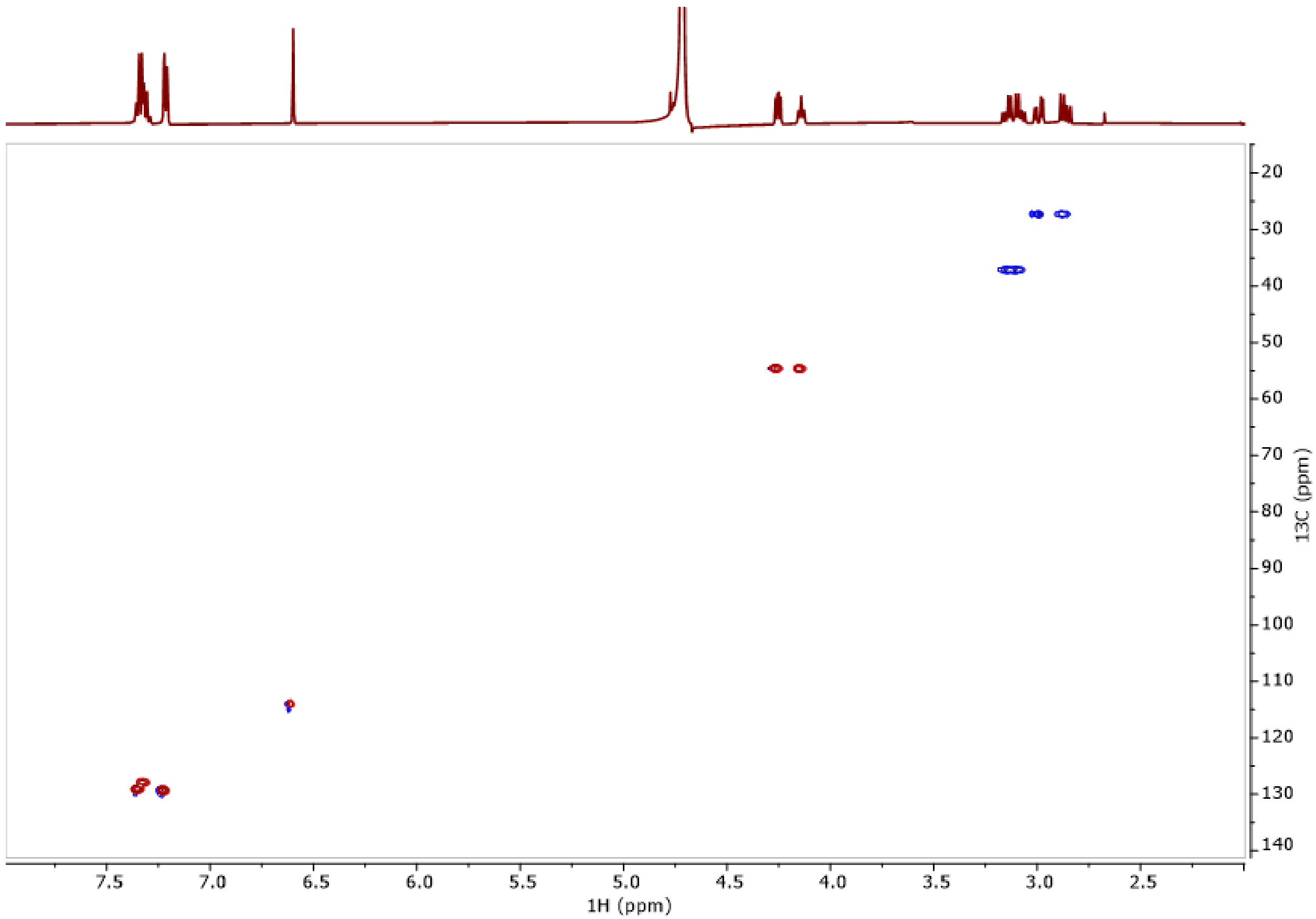

(D) ^{1}H-^{13}C HMBC spectrum of actinothioneine in D_2O at 500 MHz.

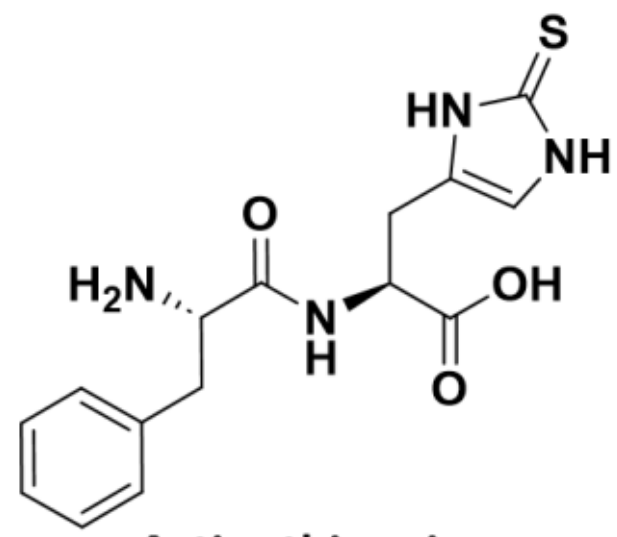

Actinothioneine

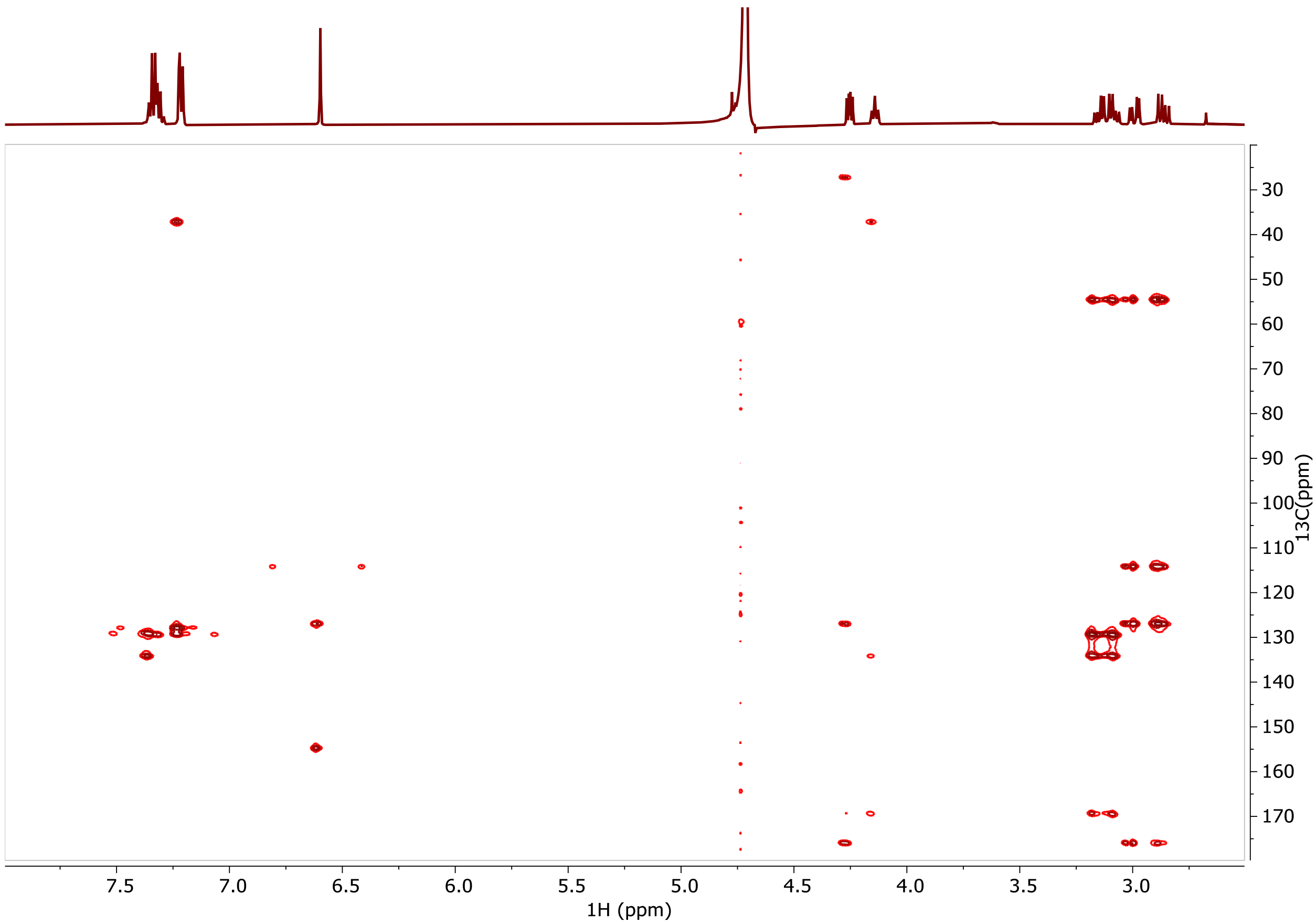

Figure S9. Heterologous expression of the actinothioneine biosynthetic genes in *E. coli*. (A) Extracted ion chromatogram for compounds within the actinothioneine biosynthetic pathway after expression of *ash* cluster in *E. coli*. No product is observed the empty plasmid control; neither is the 2-thiohistidine sulfoxide. However, both mBBr-derivatized 2-thiohistidine (dark purple) and mBBr-derivatized actinothioneine (maroon, 7.85 min) can be detected in the production strain. (B) Extracted ion chromatograms for all dipeptides upon expression of the ash cluster in *E. coli.* Only actinothioneine (Phe–2-thiohistidine) is biosynthesized under these conditions. (C) Co-injection of enzymatic standard of mBBr-derivatized actinothioneine and the *E. coli* synthesized product show that they are identical.

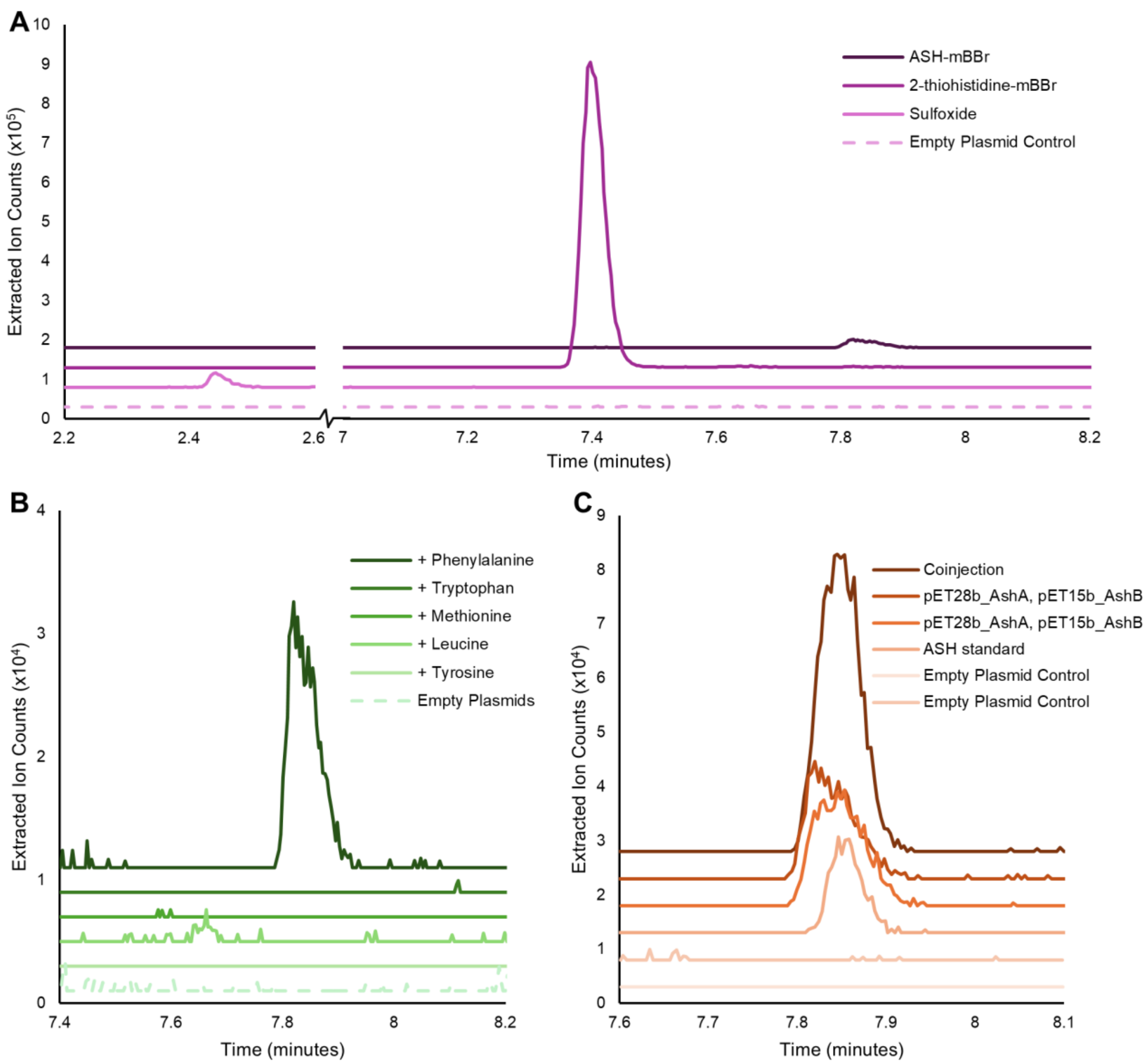

Figure S10. Active site features of AshA. (A) Composite omit map (Fo-Fc) for histidine bound to the AshA active site, contoured at 1.0 sigma. (B) Direct and water-mediated hydrogen bonds between both histidine ligands. Cysteine from OvoA (PDB ID 8KHQ) is shown in cyan. In the absence of cysteine, a second histidine (His2) coordinates to the AshA active site.

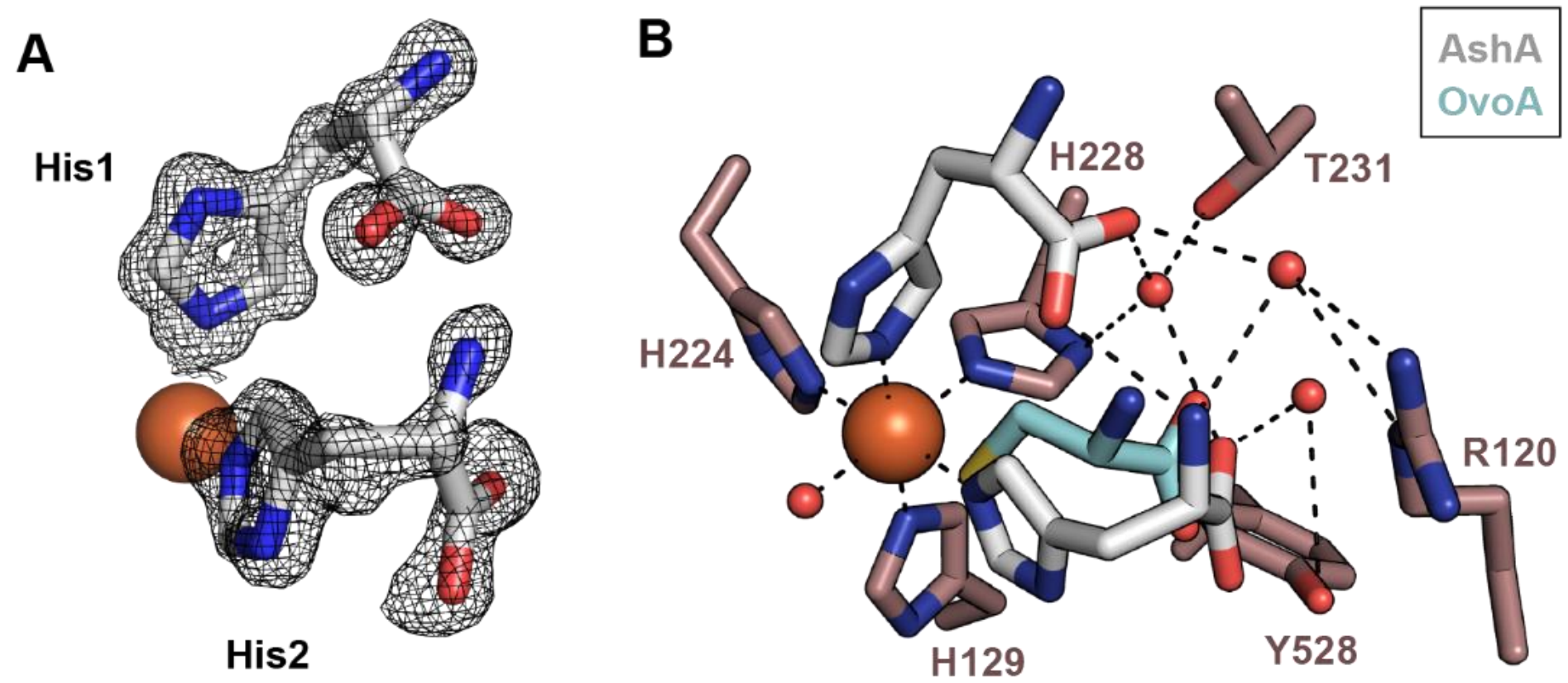

Figure S11. NMR of mBBr-derivatized thioglutine. Spectra were recorded in D_2O at 800 MHz. Shown, in order, are (A) ^{1}H, (B) ^{1}H-^{1}H NOESY, (C) ^{1}H-^{1}H COSY, (D) ^{1}H-^{13}C HSQC, and (E) ^{1}H-^{13}C HMBC spectra.

(A) ^{1}H spectrum of mBBr-derivatized thioglutine in D_2O at 800 MHz.

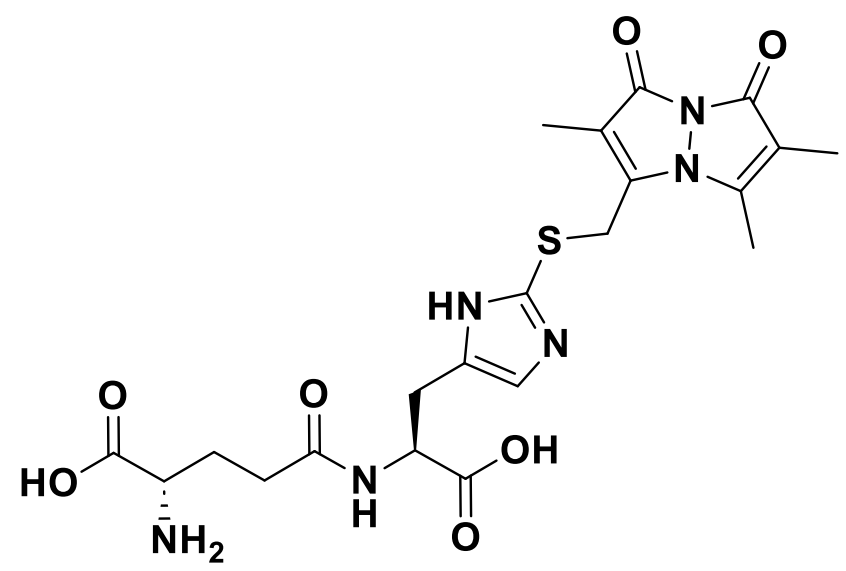

mBBr–Thioglutine

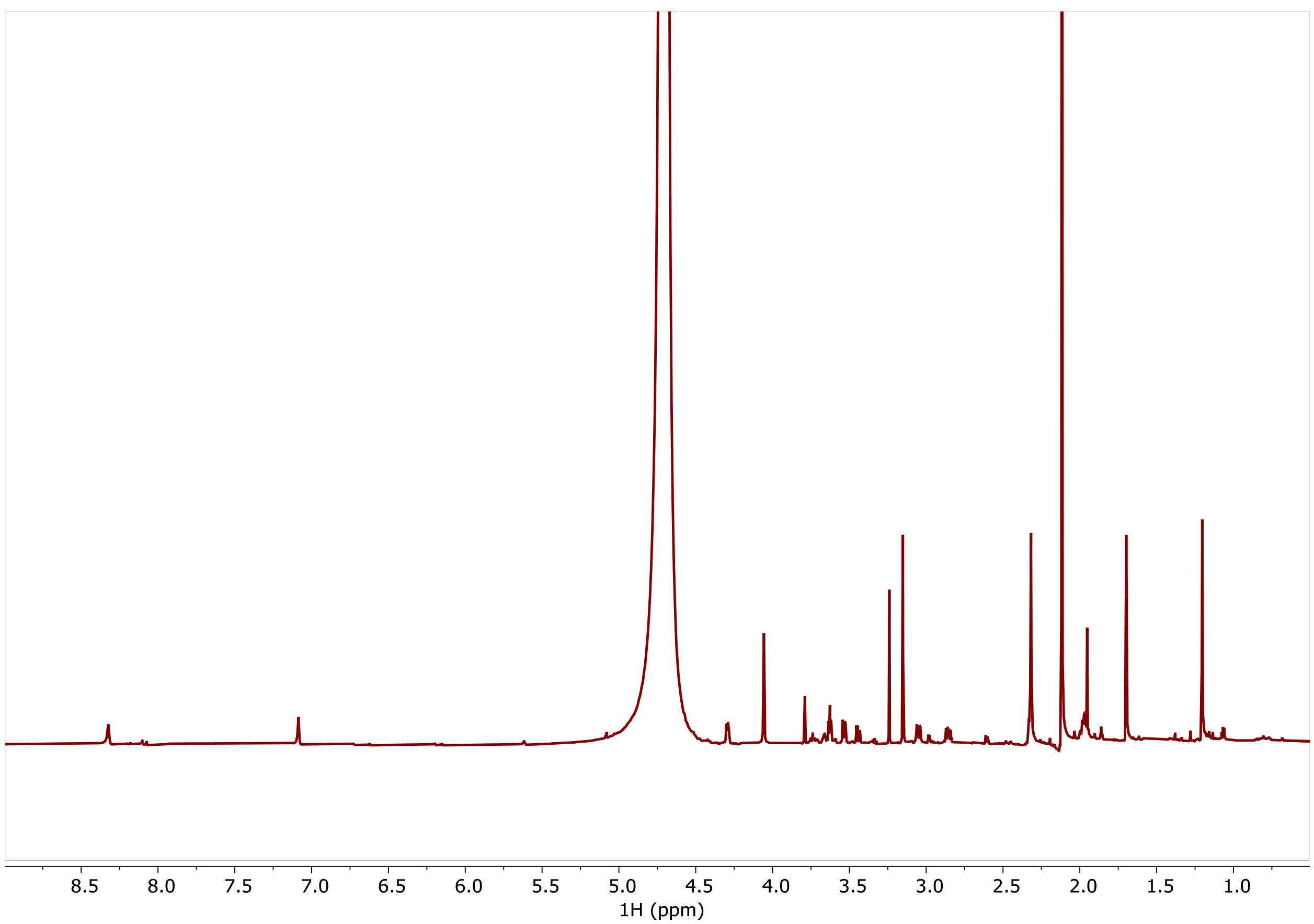

(B) ^{1}H-^{1}H NOESY spectrum of mBBr-derivatized thioglutine in D_2O at 800 MHz.

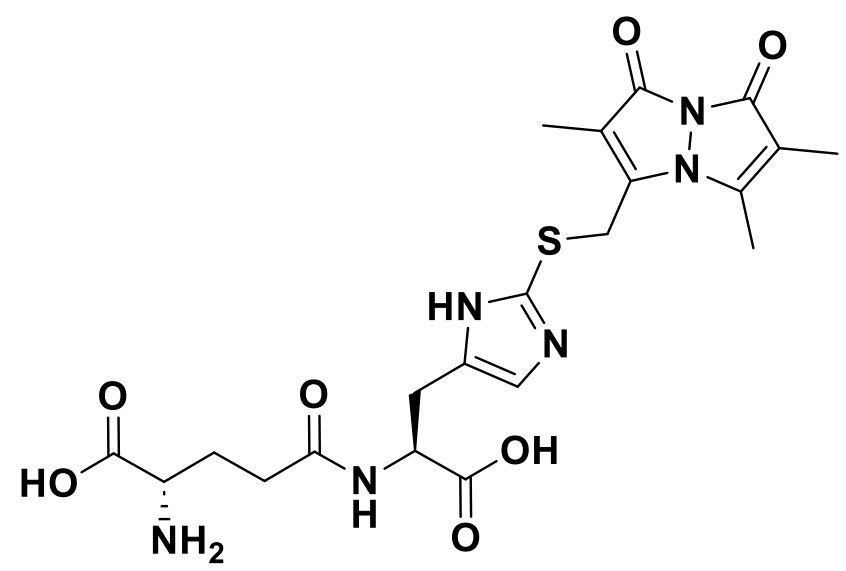

mBBr–Thioglutine

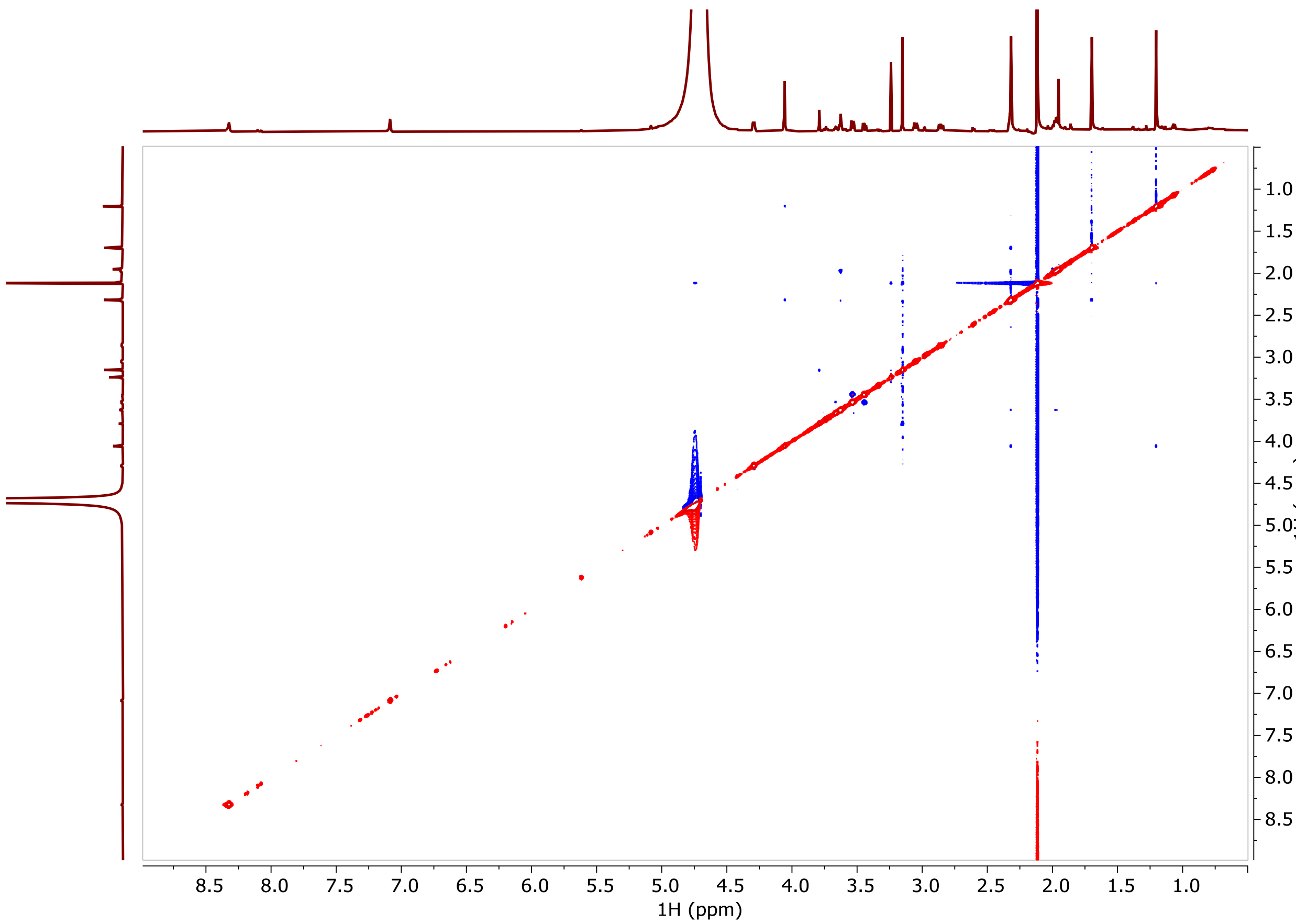

(C) ^{1}H-^{1}H COSY spectrum of mBBr-derivatized thioglutine in D_2O at 800 MHz.

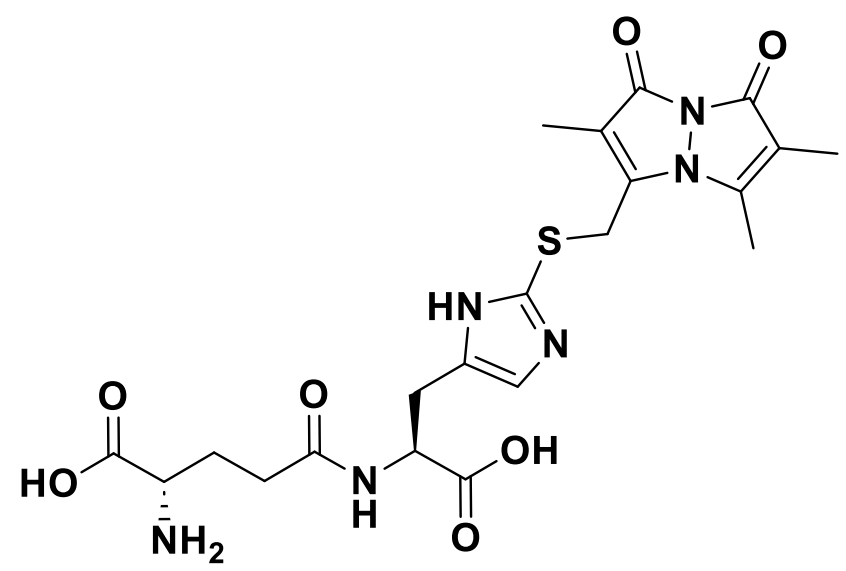

mBBr–Thioglutine

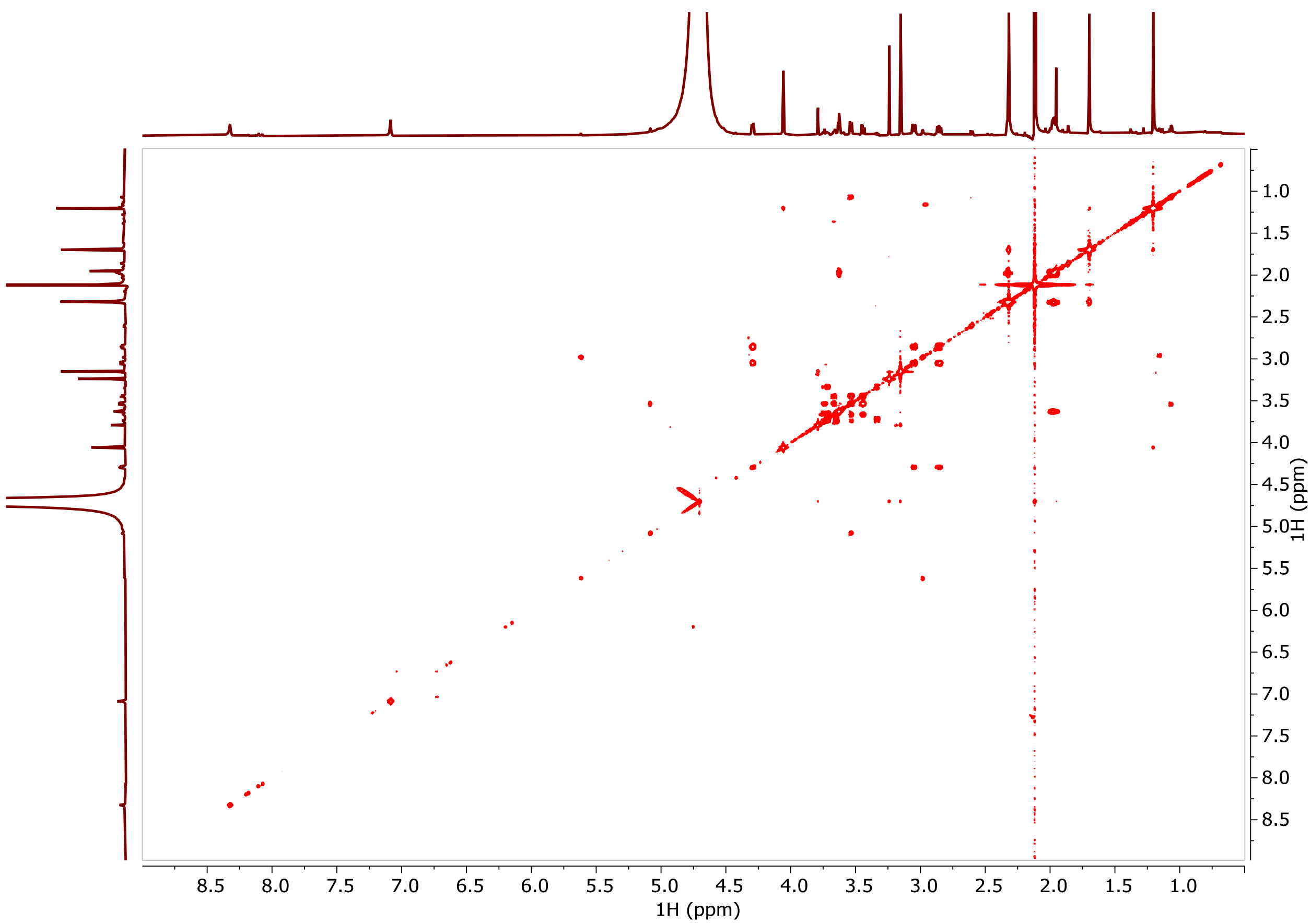

(D) ^{1}H-^{13}C HSQC spectrum of mBBr-derivatized thioglutine in D_2O at 800 MHz.

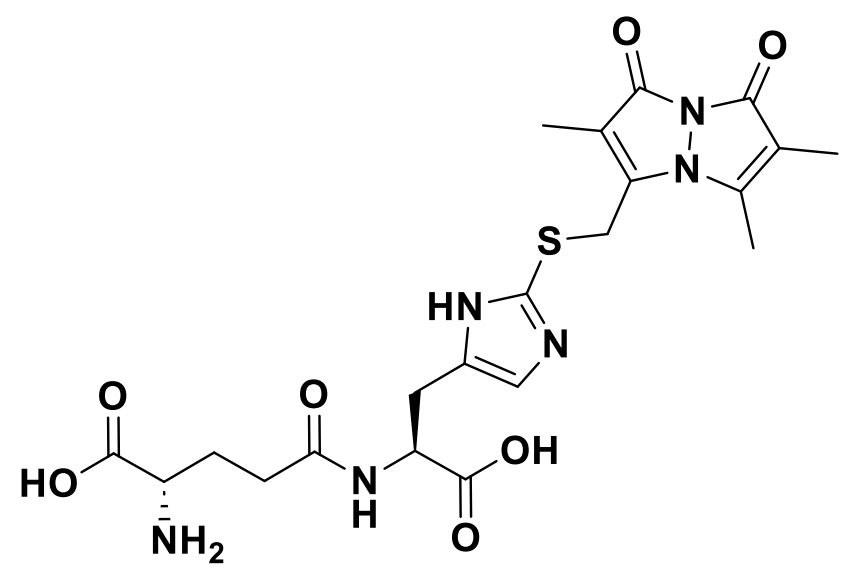

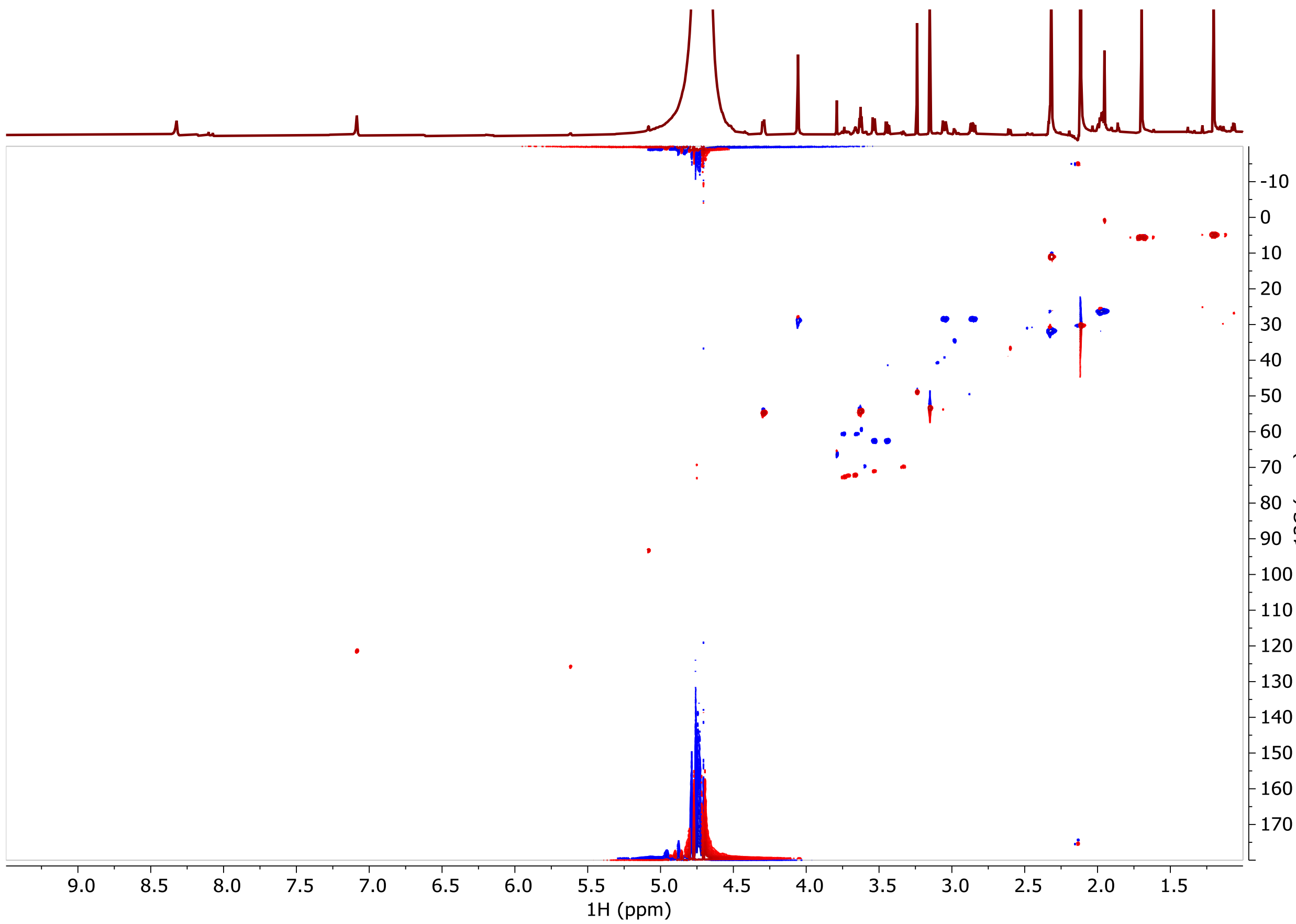

(E) ^{1}H-^{13}C HMBC spectrum of mBBr-derivatized thioglutine in D_2O at 800 MHz.

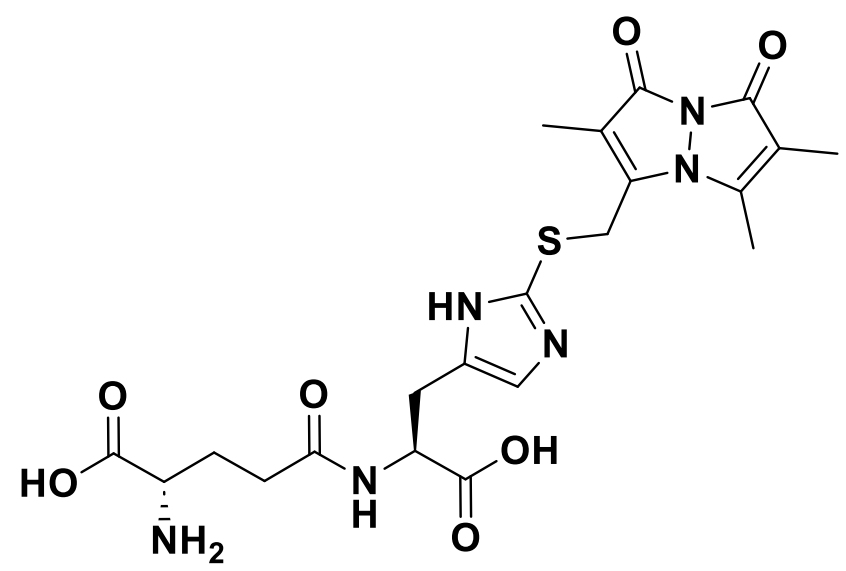

mBBr–Thioglutine

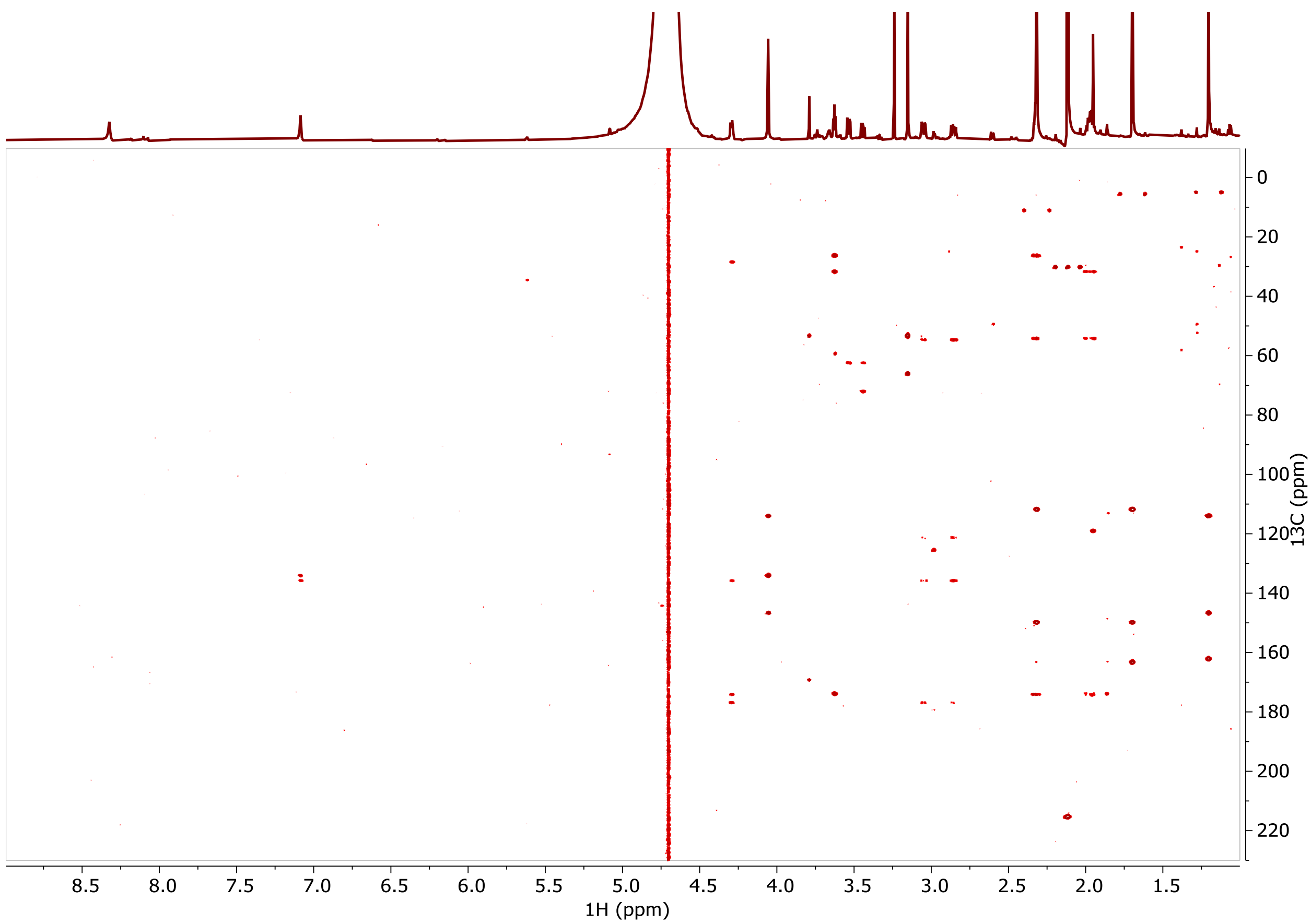

Figure S12. OvoA-like branch of the NHISS enzyme family. (A) Cladogram of all enzymes in the OvoA-like branch. Non-OvoA enzymes are highlighted. The section of the cladogram that is the focus of panel B is highlighted by an arc. (B) A zoomed-in view of the marked branch of the phylogenetic tree as noted in panel A. All newly-reported enzyme families are bolded. (C) Table of all large or divergent subfamilies in the OvoA-like branch with the most common composite motif and an example species with that composite motif. All confirmed or putative iron-binding residues are highlighted in green, cysteine-binding in red, and catalytic tyrosines in yellow. Histidine/hercynine binding residues are highlighted in blue. The only classes with confirmed binding residues are OvoA[11], EgtB-IV[10], and AshA.

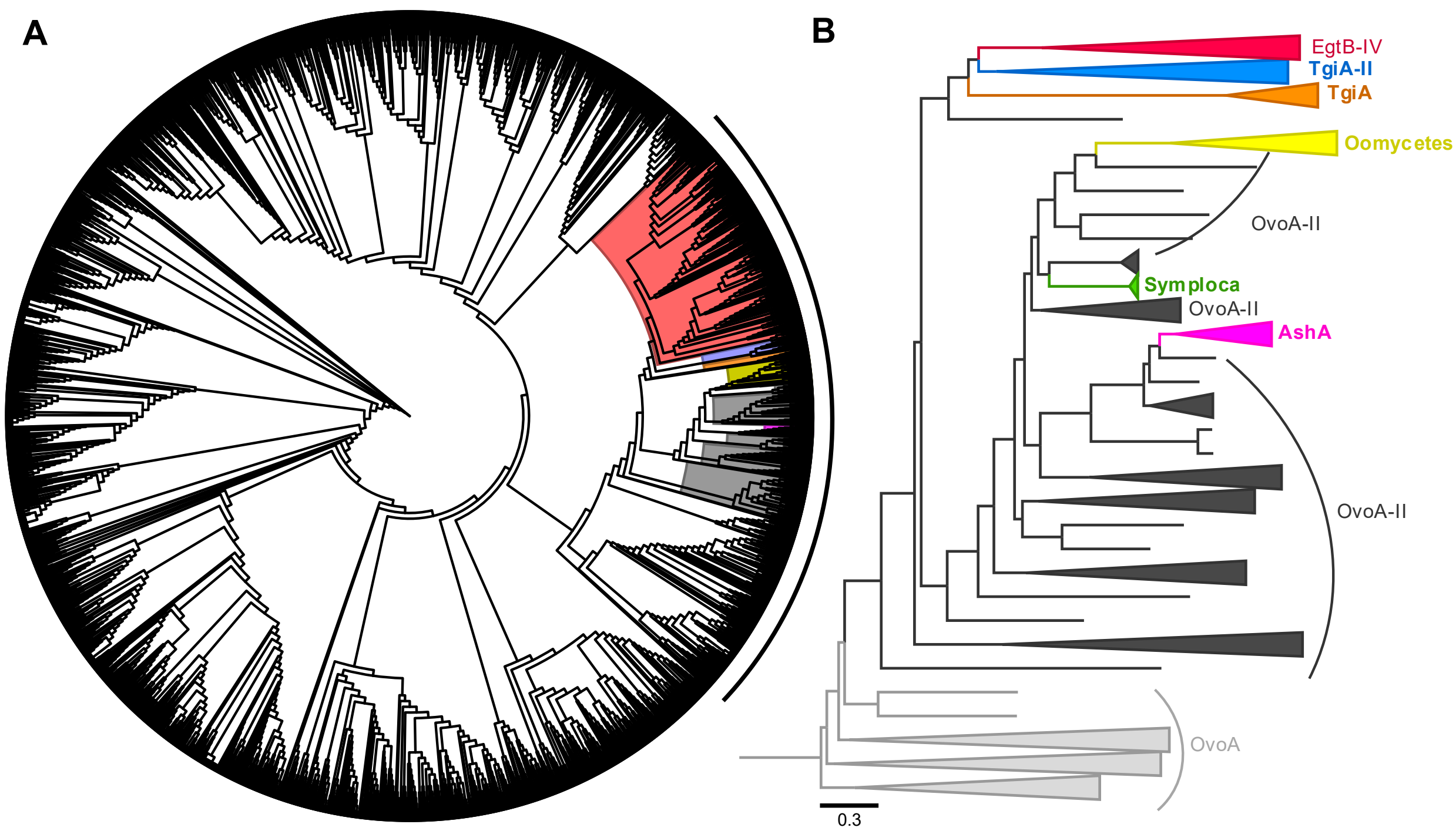

C

NHISS Subfamily	# of members	Most Common Composite Motif	Example (NCBI accession, strain)
OvoA	4375	RHYH--DSHIHYFYAFF	SPF54216.1, *H. thermophila*
OvoA-II	183	RHYH--DRHIHYFFHFF	WP_237218172.1, *R. aromaticivorans*
EgtB-IV	466	RNYH--DTHIHYQNWFF	WP_190564739.1, *A. sphaerica*
AshA	15	RHYH--DSHIHYFHFYF	WP_063272911.1, *A. keratiniphila*
TgiA	16	RLYH--SDHIHYFDFMM	WP_078645114.1, *S. varsoviensis*
TgiA-II	27	RNYH--DLHIHYFLWFF	MCR9287756.1, Bacteroidota bacterium
Oomycetes	54	RHYH--DRHIHYFYHFF	KAG6583167.1, *P. cinnamomi*
Symploca	3	RYYH--DPHIHYFFNIF	NER49683.1, *Symploca* sp.

Figure S13. $TgiB\text{-}II_{Sbu}$ generates thioglutine. Reaction of $TgiB\text{-}II_{Sbu}$ with all 20 canonical amino acids and 2-thiohistidine; the extracted ion chromatogram is shown for the mass of mBBr–thioglutine. No other masses corresponding to dipeptide products are observed. The analogous reaction with $TgiB_{San}$ is included as a positive control for retention time comparison.

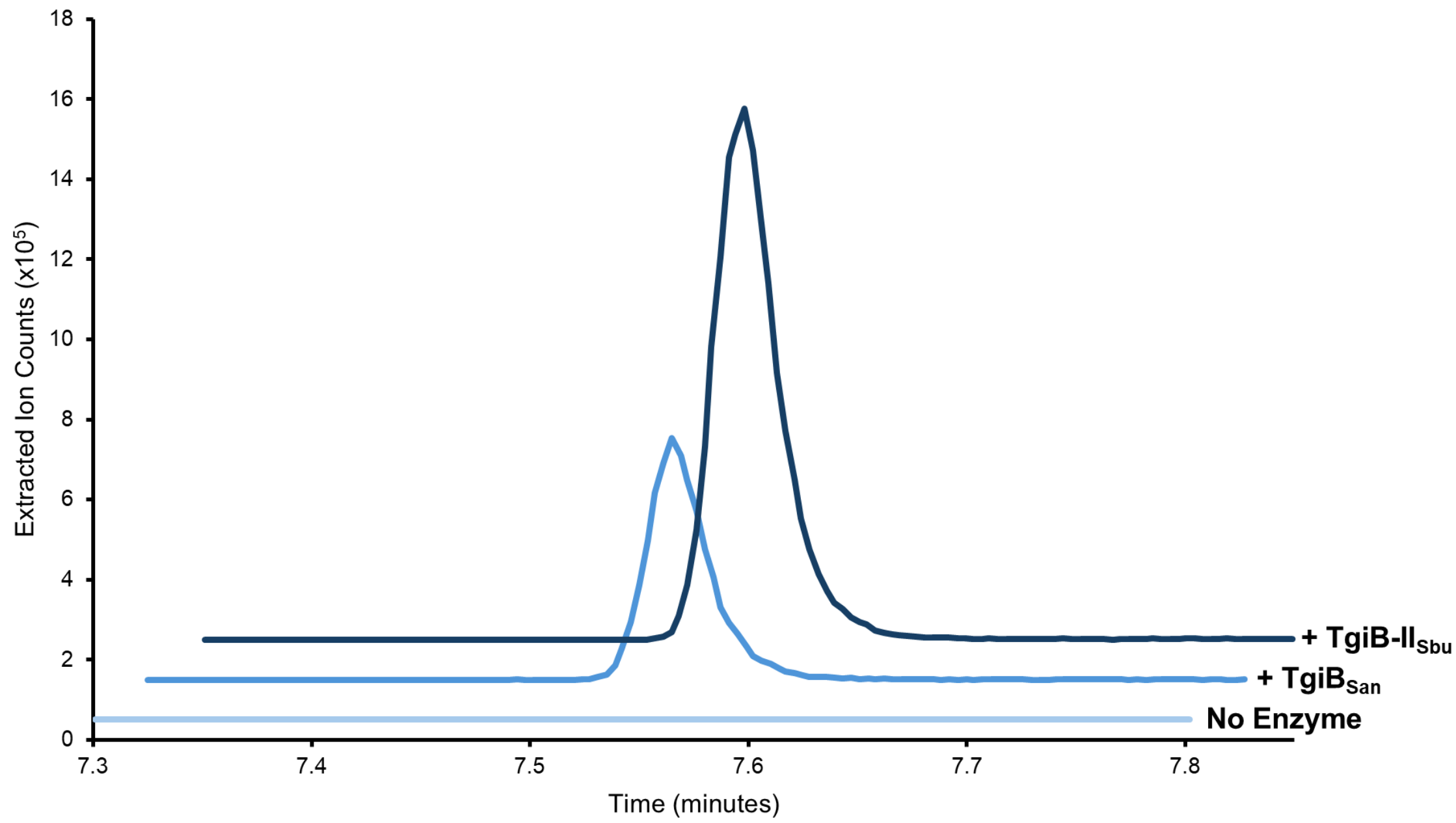

Supplementary References

(1) Katoh, K.; Standley, D. M. MAFFT Multiple Sequence Alignment Software Version 7: Improvements in Performance and Usability. *Mol. Biol. Evol.* **2013**, *30*, 772–780.

(2) Price, M. N.; Dehal, P. S.; Arkin, A. P. FastTree 2 - Approximately Maximum-Likelihood Trees for Large Alignments. *PLoS One* **2010**, *5*, e9490.

(3) Kabsch, W. *XDS*. *Acta Crystallogr. D Biol. Crystallogr.* **2010**, *66*, 125–132.

(4) Evans, P. R.; Murshudov, G. N. How Good Are My Data and What Is the Resolution? *Acta Crystallogr. D Biol. Crystallogr.* **2013**, *69*, 1204–1214.

(5) Jumper, J.; Evans, R.; Pritzel, A.; Green, T.; Figurnov, M.; Ronneberger, O.; Tunyasuvunakool, K.; Bates, R.; Žídek, A.; Potapenko, A.; Bridgland, A.; Meyer, C.; Kohl, S. A. A.; Ballard, A. J.; Cowie, A.; Romera-Paredes, B.; Nikolov, S.; Jain, R.; Adler, J.; Back, T.; Petersen, S.; Reiman, D.; Clancy, E.; Zielinski, M.; Steinegger, M.; Pacholska, M.; Berghammer, T.; Bodenstein, S.; Silver, D.; Vinyals, O.; Senior, A. W.; Kavukcuoglu, K.; Kohli, P.; Hassabis, D. Highly Accurate Protein Structure Prediction with AlphaFold. *Nature* **2021**, *596*, 583–589.

(6) Emsley, P.; Lohkamp, B.; Scott, W. G.; Cowtan, K. Features and Development of Coot. *Acta Crystallogr. D Biol. Crystallogr.* **2010**, *66*, 486–501.

(7) Adams, P. D.; Afonine, P. V.; Bunkóczi, G.; Chen, V. B.; Davis, I. W.; Echols, N.; Headd, J. J.; Hung, L. W.; Kapral, G. J.; Grosse-Kunstleve, R. W.; McCoy, A. J.; Moriarty, N. W.; Oeffner, R.; Read, R. J.; Richardson, D. C.; Richardson, J. S.; Terwilliger, T. C.; Zwart, P. H. PHENIX: A Comprehensive Python-Based System for Macromolecular Structure Solution. *Acta Crystallogr. D Biol. Crystallogr.* **2010**, *66*, 213–221.

(8) Williams, C. J.; Headd, J. J.; Moriarty, N. W.; Prisant, M. G.; Videau, L. L.; Deis, L. N.; Verma, V.; Keedy, D. A.; Hintze, B. J.; Chen, V. B.; Jain, S.; Lewis, S. M.; Arendall, W. B.; Snoeyink, J.; Adams, P. D.; Lovell, S. C.; Richardson, J. S.; Richardson, D. C. MolProbity: More and Better Reference Data for Improved All-Atom Structure Validation. *Protein Science* **2018**, *27*, 293–315.

(9) Schrodinger, LLC. The PyMOL Molecular Graphics System, Version 2.5.5. 2023. **2023**.

(10) Ireland, K. A.; Kayrouz, C. M.; Abbott, M. L.; Seyedsayamdost, M. R.; Davis, K. M. Structural Insights into the Convergent Evolution of Sulfoxide Synthase EgtB-IV, an Ergothioneine-Biosynthetic Homolog of Ovothiol Synthase OvoA. *Structure* **2024**, *32*, 2013–2022.

(11) Wang, X.; Hu, S.; Wang, J.; Zhang, T.; Ye, K.; Wen, A.; Zhu, G.; Vegas, A.; Zhang, L.; Yan, W.; Liu, X.; Liu, P. Biochemical and Structural Characterization of OvoATh2: A Mononuclear Nonheme Iron Enzyme from *Hydrogenimonas thermophila* for Ovothiol Biosynthesis. *ACS Catal.* **2023**, *13*, 15417–15426.
